# A Cationic NHC‐Supported Borole

**DOI:** 10.1002/chem.202001916

**Published:** 2020-08-13

**Authors:** Tobias Heitkemper, Christian P. Sindlinger

**Affiliations:** ^1^ Institut für Anorganische Chemie Georg-August-Universität Göttingen Tammannstr. 4 37077 Göttingen Germany

**Keywords:** borenium cation, boroles, CO-complex, maingroup heterocycles

## Abstract

This work describes the synthesis and characterization of a highly reactive cationic borole. Halide abstraction with Li{Al[OC(CF_3_)_3_]_4_} from the NHC‐chloroborole adduct yields the first stable NHC‐supported 1‐(^Me^NHC)‐2,5‐(SiMe_3_)_2_‐3,4‐(Ph*)_2_‐borole cation. Electronically, it features both a five‐membered cyclic conjugated 4 π‐electron system and a cationic charge and thus resembles the yet elusive cyclopentadienyl cation. The borole cation was characterized crystallographically, spectroscopically (NMR, UV/Vis), by cyclovoltammetry, microanalysis and mass‐spectrometry and its electronic structure was probed computationally. The cation reacts with tolane and reversibly binds carbon monoxide. Direct comparison with the structurally related, yet neutral, 1‐mesityl borole reveals strong Lewis acidity, reduced HOMO–LUMO gaps, and increased *anti*‐aromatic character.

## Graphical abstract

**Think positive**! Halide abstraction from an NHC‐adduct to a haloborole allowed for the synthesis of the first cationic borole featuring a three‐coordinate boron‐atom embedded in a cyclic 4π electron system. This cation reveals high boron‐centered Lewis‐acidity and a very small reduction potential. CO readily and reversibly binds to the boron‐center to form a rare carbonyl complex with considerably low stretching frequency that indicates backbonding.

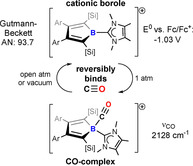

The cyclopentadienyl cation [C_5_H_5_]^+^ often serves as a textbook case for *anti*‐aromaticity according to Breslow's 4 π‐electron extension of Hückel's theory on aromaticity.^1^ While some homoleptic derivatives of cyclopentadienyl cation [C_5_R_5_]^+^ (R=H or Cl) have triplet ground states and thus are aromatic according to Baird's rule,^2^ particularly heteroleptic derivatives of reduced symmetry [(ClC)_4_CR′] (R′=[H^+^], BF_3_) with singlet ground state are considered *anti*‐aromatic.^3^ However, all these derivatives are elusive and have only been generated, “isolated” and characterized in frozen solvent, solid SbF_5_ or inert noble‐gas matrices and the synthesis of (singlet) cyclopentadienyl cations remains challenging.3b Free, neutral boroles ((RC)_4_BR′) are isoelectronic to cyclopentadienyl cations and thus they reveal high reactivity associated with their (weak) *anti*‐aromatic nature.^4^ Yet, some free boroles are synthetically accessible and isolable granting a broader set of analytical techniques to probe these species (Scheme 1).4b, 5 It has been postulated that by tuning the frontier orbital situation of cyclopentadienyl cations to the one in boroles through variation of inductively active substituents in [(RC)_4_CR′]^+^, stable derivatives may be accessible.^6^ The opposite approach to stable electronic mimics of cyclopentadienyl cations would be to electronically disguise a boron atom in boroles as a carbon atom. In our attempts to do so, we were able to isolate a borole‐derived borenium cation with a three‐coordinate boron atom that still features cyclic conjugation of the four‐electron π‐system. It thus represents both a cationic, borole‐derived electronic mimic to cyclopentadienyl cation as well as an extension of a series of formal single electron additions to (PhC)_4_BR′ (R′=Aryl or NHC) (Scheme 1).^7^

**Scheme 1:**
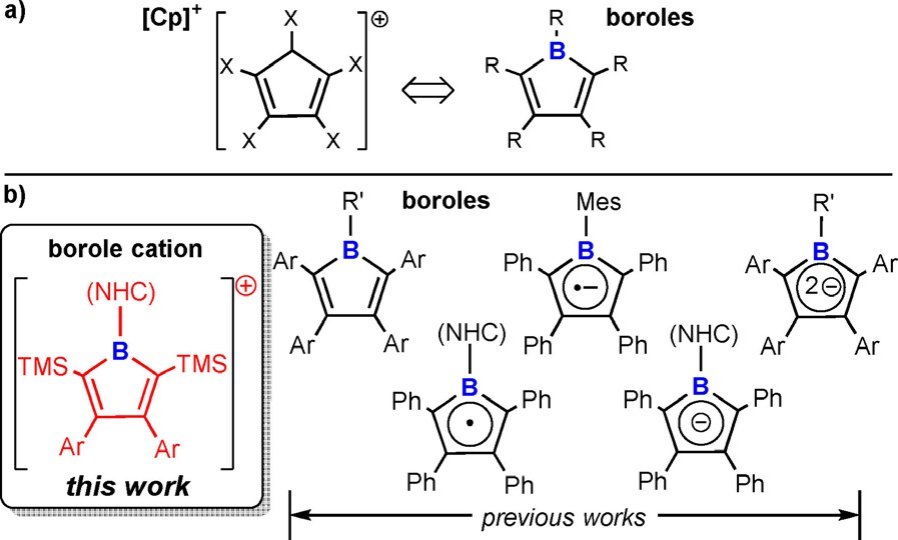
a) Electronic analogy of singlet cyclopentadienyl cations and boroles. b) One‐electron‐addition series of known neutral and charged borole derivatives and the borole cation. Only one mesomeric structure is shown for each.

A borole‐derived boronium cation with a tetra‐coordinated boron atom and thus without any cyclic delocalization of the π‐system was observed by Braunschweig, when two molecules of 4‐methylpyridine replace a chloride in (PhC)_4_BCl.^8^ Donor‐supported 9‐borafluorene cations have been described by Nöth and most recently by Gilliard.^9^ However the central (C_4_B) 4 π‐system in borafluorenes is conjugated with benzene moieties and delocalized, significantly reducing the *anti*‐aromatic character and reactivity.^10^ Therefore borafluorenes are not suitably comparable to actual free boroles.

We have recently found access to a thermally stable chloroborole **A‐Cl**.^11^ Addition of 1 equiv of 1,3‐(R)_2_‐4,5‐dimethylimidazol‐2‐ylidenes (R=Me, ^Me^NHC; R=*i*Pr, ^*i*Pr^NHC; NHC=*N*‐heterocyclic carbene) to solutions of **A‐Cl**, gives crystalline colourless NHC‐adducts **1 a** and **1 b** in ca. 70 % yield (Scheme 2). The ^1^H NMR spectrum of **1 a,b** reveals four signals sets for the four alkyl groups of the NHC in line with no rotation of the NHC around the B−C bond within the NMR timescale. The ^11^B NMR resonance shifts from *δ*_11B_=70.8 ppm (**A‐Cl**) to *δ*_11B_=0.5 (**1 a**) and 1.3 (**1 b**) ppm fully in line with a tetracoordinate boron atom. The crystal structure of **1 a** is documented in the Supporting Information.

**Scheme 2:**
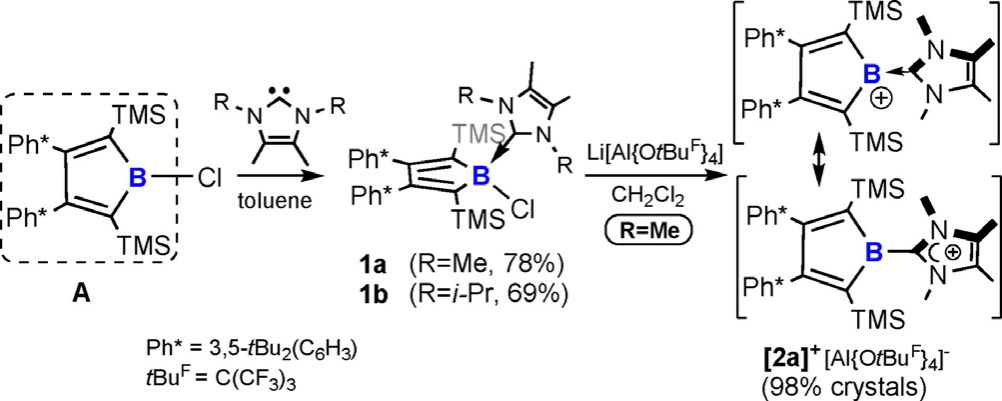
Synthesis of precursors **1 a,b** and borole cation **[2 a]^+^**.

A few examples of NHC‐adducts to haloboroles have previously been reported,7a, 12 however usually applying bulky *N*‐aryl imidazole‐2‐ylidenes. Braunschweig and co‐workers obtained a borole monoanion with B‐centred nucleophilicity by reducing such an adduct of (PhC)_4_BCl.7a

When solutions of **1 a** in dichloromethane are treated with Na[B(Xyl^F^)_4_] (Xyl^F^=3,5‐(CF_3_)_2_(C_6_H_3_)), immediate quantitative consumption of **1 a** yields dark yellow‐green solutions and sodium chloride precipitates. NMR‐spectroscopic examination of these samples kept below −15 °C reveal clean formation of a single new product with one signal set for the *N*‐Me groups of the NHC, but the new compound decomposes swiftly at room temperature. Dehalogenation with Krossing's superbly inert and weakly coordinating anion salt Li[Al{OC(CF_3_)_3_}_4_]^13^ in CH_2_Cl_2_ leads to the same immediate formation of a single new compound with no sign of decomposition. Similar dehalogenation approaches previously allowed for the synthesis of, for example, highly reactive borinium cations.^14^ Single‐crystal diffraction confirmed the formation of the cationic borole [**2 a**]^**+**^ (Figure 1) being an unprecedented type of NHC‐supported borenium cations.^15^ Compound [**2 a**][Al{OC(CF_3_)_3_}_4_] can be isolated as a dark yellow‐green crystalline material quantitatively (98 %) and is stable in solution for several days at room temperature but is extremely sensitive to oxygen and water and immediately decomposes in ambient atmosphere.

**Figure 1:**
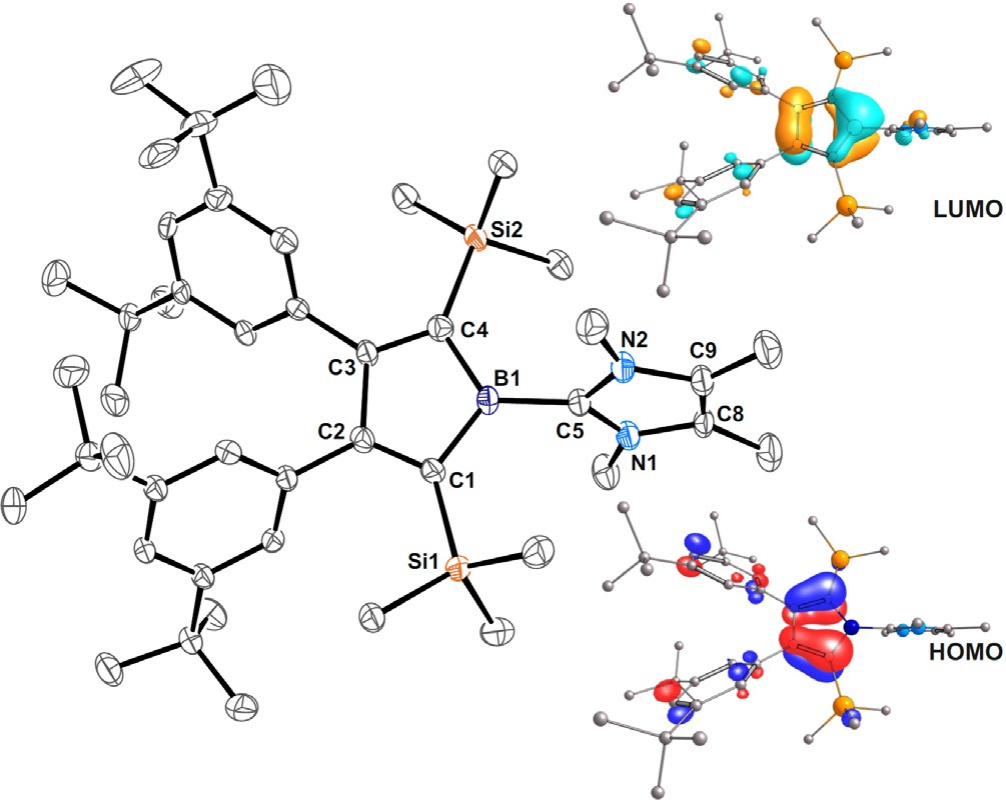
ORTEP of the solid state structure of borole cation **[2 a]^+^** and depictions of the frontier orbitals (isovalue 0.05 a.u.). Hydrogen atoms, the [Al{OC(CF_3_)_3_}_4_] anion, lattice CH_2_Cl_2_ and disorder in *t*Bu‐groups are omitted for the sake of clarity. Anisotropic displacement parameters are drawn at 50 % probability. Selected bond lengths [Å]: B1−C5 1.588(8), C5−N1 1.332(7), C5−N2 1.347(5), see also Table 1.

Within the C_4_B cycle, the structure features two localized double bonds as to be expected for boroles and Jahn–Teller distorted singlet cyclopentadienyl cations. A fairly large torsion angle of 70(2)° between the NHC‐plane and the C_4_B‐plane is observed. Likely due to steric reasons, the two silyl groups both are mildly tilted out above and below the C_4_B plane. The structural features of [**2 a**]^**+**^ are virtually identical to its most related neutral derivative featuring a boron‐bound mesityl (Mes; 2,4,6‐Me_3_(C_6_H_2_)) group (**A‐Mes**) (Table 1).^11^ ^Me^NHC and Mes feature a similar steric profile. NMR‐spectroscopy reveals for [**2 a**]^**+**^ a ^11^B NMR resonance at *δ*_11B_=73.9 ppm pronouncedly less low‐field shifted than comparable neutral aryl boroles^11^, ^16^ including **A‐Mes** or [R_2_B(NHC)]^+^ borenium cations (Table 1).15d, 15e The ^13^C NMR resonances within the C_4_B‐cycle are found at *δ*_13C_=135.5 ppm (C_α_) and 190.3 ppm (C_β_) with C_β_ being significantly low‐field shifted compared to **A‐Mes**. Particularly the combination of relatively highfield‐shifted ^11^B and low‐field shifted C_β_^13^C resonances are in line with an increased donation of π‐electron density from the butadiene‐system into the energetically very low‐lying empty *p*‐orbital at boron in this cationic borole. This can be rationalized by the contribution of resonance structures of type **III** (Scheme 3), essentially meaning slightly improved conjugation in cationic boroles compared to the free neutral boroles. This spectroscopic insight is corroborated by NBO^17^ and second order perturbation theory that predicts a slightly stronger donation in [**2 a**]^**+**^ (35.2 kJ mol^−1^) than in **A‐Mes** (25.6 kJ mol^−1^). The out‐of‐(C_4_B)‐plane component of the nuclear independent chemical shift (NICS_zz_),^18^ as a measure for *anti*‐aromatic character, is increased compared to **A‐Mes** (Table 1) and nearly identical to those of inductively withdrawing substituted systems as in **A**‐**C_6_F_5_** (NICS_zz_(1)=30.3).^11^

**Table 1:** Structural, spectroscopic and computational characteristics of borole cation **[2 a]^+^** and their comparison to mesityl‐borole **A‐Mes**.

Entry	B−C_α1_, C_α1_−C_β1_, C_β1_−C_β2_ B−C_α2_, C_α2_−C_β2_^[a]^	C_4_B‐Ar torsion ^[b]^	*δ*(^13^C), (C_α_,C_β_)^[c]^	*δ*(^11^B)^[c]^	*λ*_exp_^[d]^, (*λ*_calc_)^[e]^, *ϵ*_λ_^[f]^	HOMO/LUMO/ gap^[g]^	NICS_zz_(0) NICS_zz_(1)^[h]^	*E* ^0[i]^	*δ* ^31^P AN^[j]^
[**2 a**]^+^	1.570(8), 1.351(7), 1.545(7) 1.550(8), 1.359(7)	70(2)	135.5, 190.3	73.9	564, (579), 180	−7.63/−6.38/ 1.25	55.6 30.8	−1.03	83.4 93.7
**A‐Mes^[11]^**	1.590(3), 1.360(4), 1.538(4) 1.594(4), 1.359(3)	85(1)	137.7, 181.0	79.9	≈480, (462), ≈400	−5.17/−3.45/ 1.72	48.5 25.5	−1.91	46.1 11.3

[a] In [Å]; [b] Torsion angle in [°]; [c] in CD_2_Cl_2_ in parts per million, ppm; [d] Absorption bands of lowest energy in nm in CH_2_Cl_2_; [e] TD‐DFT: RIJCOSX‐CAM‐B3LYP\def2‐SVP\\RI‐BP86‐D3BJ\def2‐TZVP (for [**2 a**]^**+**^: CPMC: CH_2_Cl_2_); [f] in L mol^−1^ cm^−1^ (CH_2_Cl_2_); [g] RI‐BP86\def2‐TZVP, gas phase energies in eV; [h] NICS_zz_(0) and NICS_zz_(1) GIAO PBE0\def2‐TZVP; [i] Potential of the first reduction wave in V given versus Fc/Fc^+^ (0.04 m [*n*Bu_4_N][Al{OC(CF_3_)_3_}_4_] in 1,2‐difluorobenzene); [j] Gutmann–Beckett Lewis acidity scale parameters derived from mixtures with Et_3_PO in C_6_D_6_ (**A‐Mes**) or CD_2_Cl_2_ ([**2 a**]^**+**^). Acceptor Numbers AN=2.21×(*δ* ^31^P −41). Calculations were performed using ORCA4.1.^20^

**Scheme 3:**
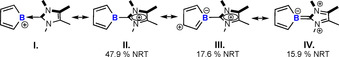
Mesomeric contributions to the electronic structure of a model cationic NHC‐supported borole (HC)_4_B(^Me^NHC) including the dominant contributions according to NRT calculations.^19^ NBO charges: +0.34 [(HC)_4_B]‐fragment, +0.66 [^Me^NHC]‐fragment.

Most insightful for the evaluation of the frontier orbital situation in boroles is the optically detectable C_4_B‐centered π/π* transition (Figure 2).

**Figure 2:**
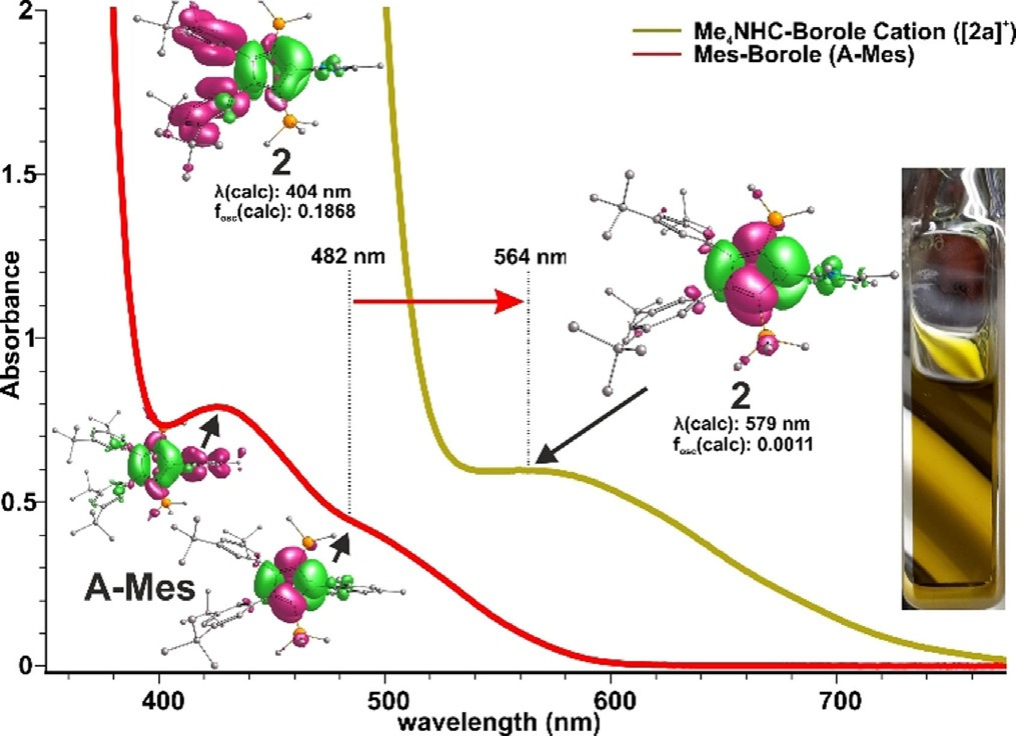
UV/Vis spectra of cationic borole [**2 a**]^+^ and neutral borole **A‐Mes** and difference density plot depictions (green: positive Δ*ρ*, magenta: negative Δ*ρ*, isolevel at 0.001 a.u.) of the computationally reproduced corresponding excitations. (TDDFT: RIJCOSX‐CAM‐B3LYP/def2‐SVP/CH_2_Cl_2_).^20^

While pentaaryl boroles reveal absorptions around 570 nm, respective transitions in neutral 2,5‐disilyl boroles are blue‐shifted to 470 nm due to reduced π‐interaction of the C_4_B nucleus with its arene substituents.5h, 11 Yellow‐greenish solutions of borole cation [**2 a**]^**+**^ reveal a weak absorption band corresponding to this π/π*‐transition at *λ*_max_=564 nm (Figure 2). The yellow colour stems from a much more intense absorption around 390 nm. Compared to **A‐Mes** (*λ*_max_ 482 nm) the cationic borole [**2 a**]^**+**^ thus reveals a considerably redshifted π/π*‐absorption band. This is in excellent agreement with the computationally predicted absorptions (Table 1). The redshift also indicates that upon cationization of the borole, the boron‐centered LUMO is relatively more stabilized than the HOMO leading to a significantly reduced frontier orbital gap, which is also corroborated by accompanying calculations (**A‐Mes**: 1.72 eV, [**2 a**]^+^: 1.25 eV). According to UV/Vis data, the electronic impact of a cationic imidazolium substituent (Δ*λ*_max_ ca. +82 nm vs. **A‐Mes**) also greatly exceeds the impact of the strongly inductively active ‐C_6_F_5_ substituent in **A**‐**C_6_F_5_** (Δ*λ*_max_ ca. +25 nm vs. **A‐Mes**).4m, 11, 21

A low‐lying LUMO is also reflected by cyclovoltammetry. While the neutral borole **A‐Mes** reveals a reversible 1‐electron reduction at −1.91 V (vs. Fc/Fc^+^, 0.04 m [*n*Bu_4_N][Al{OC(CF_3_)_3_}_4_] in 1,2‐difluorobenzene) in a similar range as other neutral boroles (PhC)_4_BMes (−1.69 V) or (PhC)_4_BFc (−1.96 V),7b, 22 the borole cation [**2 a**]^+^ reveals a remarkably early reversible reduction at *E* ^0^=−1.03 V. This reduction even occurs at significantly less negative potentials than those reported for the reduction of [(NHC)BAr_2_]^+^ borenium cations as for example reported by Gabbaϊ or Tamm (*E* ^0^=−1.86 V, Ar=Mes; *E* ^0^=−1.56 V, Ar=*p*‐CF_3_(C_6_H_4_)).15h, 23 These experimental observations further stress, that the cationic charge, though dominantly localized within the imidazolium moiety by resonance structures, significantly affects the borole π‐system.

A further feature is the remarkably enhanced Lewis acidity of the cationic borole [**2 a**]^+^ as assessed by the Gutmann–Becket (GB) method. Addition of OPEt_3_ to solutions of [**2 a**]^+^ leads to immediate decolorization and formation of the adduct [**2 a(OPEt_3_)**]^+^ with a strongly low‐field shifted ^31^P‐NMR resonance of *δ*_31P_=83.4 ppm (CD_2_Cl_2_) and a Gutmann–Beckett acceptor number (AN) of 93.7. Similar free boroles **A‐Ar** including **A**‐**C_6_F_5_** revealed only AN of 73(±1).^11^ These observed AN values for [**2 a**]^+^ are significantly higher than, with respect to the electronegativity of three boron–bound carbon atoms, comparable planar pentacyclic [NHC‐B(Ar)_2_]^+^ (AN 84) or (m)NHC‐(9‐BBN) borenium cations (AN 78–79) reported by Crudden and Stephan.15d, 15e, 15g Other borenium cations were found more Lewis‐acidic according to GB and it was shown that direct comparisons with landmark neutral Lewis acids such as B(C_6_F_5_)_3_ are to be questioned.^24^

The adduct formation is particularly remarkable when compared to mesityl borole **A‐Mes** (AN 11.3) where almost any interaction of OPEt_3_ is ruled out due to the steric shielding of the two methyl groups in *ortho*‐position. This steric protection reduces the reactivity of mesityl boroles. Simply changing the B‐bound residue from a six‐ (**A‐Mes**) to a five‐membered ring ([**2 a**]^+^) drastically alters the accessibility of the reactive C_4_B cycle for other reagents such as diphenylacetylene (Scheme 4). While **A‐Mes** reveals no reaction under the same conditions, [**2 a**]^+^ cleanly forms the colourless Diels–Alder product [**3**]^+^ within a few hours at room temperature.^25^ [**3**]^+^ is characterized spectroscopically and its structural assignment is corroborated computationally (see Supporting Information).

**Scheme 4:**
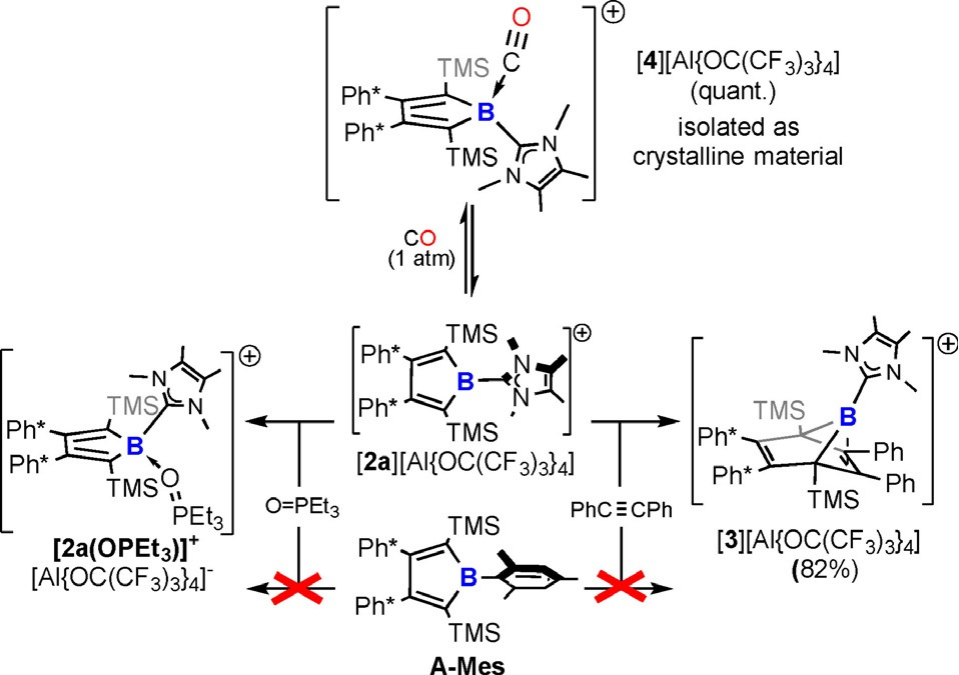
Reactivity of borole cation [**2 a**]^+^ towards diphenylacetylene and reversible formation of a CO‐adduct. Only one mesomeric structure is shown.

A high‐field shifted ^11^B NMR resonance at −11.4 ppm is in line with interactions of the Lewis‐acidic boron atom with an adjacent CC π‐bond as previously observed.^26^ Reactions of alkynes with boroles are known to form a series of products including Diels–Alder adducts, ring expanded borepins, or more complicated rearrangements, depending on the electronics and sterics of substituents.4c, 25a, 26, 27

When [**2 a**]^+^ is exposed to an atmosphere of CO, immediate decolourization and quantitative conversion to cationic CO‐adduct [**4**]^+^ is observed by NMR‐spectroscopy. Under an atmosphere of CO, [**4**]^+^ can be kept at room temperature in solution for a day allowing characterization of this kinetic product [**4**]^+^ before very slow follow‐up reactions are observed. In an (inert) open atmosphere, CO is readily liberated at room temperature and the free cation is rapidly recovered. [**4**]^+^ features a strongly high‐field shifted ^11^B resonance at −18.8 ppm, indicative for tetra‐coordinate boron. Other than previously reported borane carbonyls,4e, 28 the CO stretching frequency in [**4**]^+^ at 2128 cm^−1^ is even a bit lower than in free CO at 2143 cm^−1^. Crystals of [**4**][Al{OC(CF_3_)_3_}_4_] are obtained from solutions in dichloromethane layered with pentane at −40 °C, but they eventually liberate CO and despite great care, only a mediocre dataset was obtained by X‐ray diffraction confirming and corroborating the computational structure (see Supporting Information).

The observed IR stretching frequency in [**4**]^+^ hints at a considerable degree of π‐back donation from the borole π‐system. Such interactions were proposed to cause the lability of the pentaphenyl borole CO adduct.4e An ETS‐NOCV^29^ analysis indeed revealed a strong (443 kJ mol^−1^) σ‐donation from the CO fragment into the boron centered LUMO and a significant π‐backdonation component (109 kJ mol^−1^) (Figure 3).

**Figure 3:**
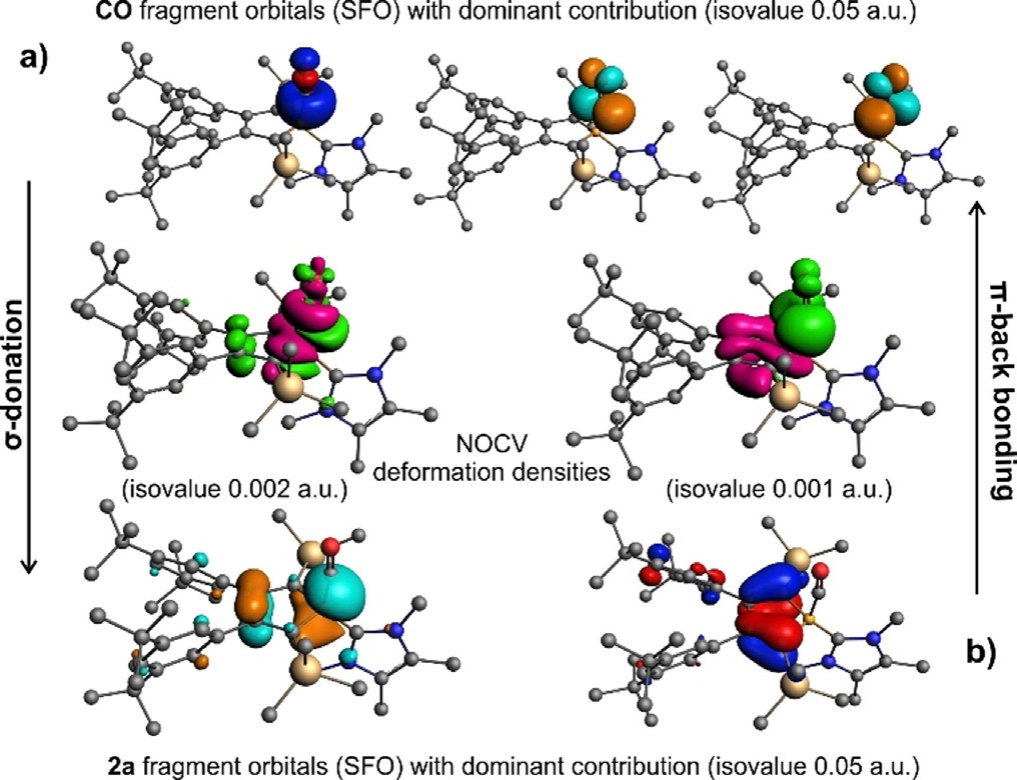
ETS‐NOCV analysis of CO and [**2 a**]^+^ fragments. Occupied SFO (blue/red), unoccupied SFO (orange/turquoise), deformation densities (green: positive, magenta: negative).^30^

The observation of the labile, yet isolable rare organoborane CO complex [**4**]^+^ is noteworthy with regards to CO adducts of neutral boroles.28b, 28c, 31 Piers and co‐workers described the only example of a stable CO‐complex of perfluorinated pentaphenyl borole with a CO stretching frequency at 2199 cm^−1^ indicative of little to no π‐backbonding.4e The non‐fluorinated pentaphenyl borole likely only forms the adduct as an intermediate at very low temperatures and undergoes insertions/rearrangements below −10 °C.4e, 5b A C_4_B based HOMO accessible for π‐interaction was considered to promote further reactivity and electron withdrawing groups favour isolation of the CO‐complex.4e, 32 Erker's related 2,5‐disilylborole even adds the CO to a C_α_ atom and gives, after rearrangements, a ketene derivative.^26^ Despite the low CO‐stretching frequency, the CO‐borole complex [**4**]^+^ more resembles Piers’ (Ph^F^C)_4_BPh^F^‐CO complex. Since the kinetic product [**4**]^+^ can be isolated, the Lewis‐acidic boron atom of the cation seems to bind CO strong enough while the C_4_B π‐system does not provide pathways of sufficiently low‐barriers that would prevent from its preliminary isolation.

Lastly, the application of the small ^Me^NHC for successful isolation of this stable borole cation system seems mandatory since analogous treatment of the sterically more demanding ^*i*Pr^NHC derivative **1 b** with Li[Al{OC(CF_3_)_3_}_4_] putatively leads to initial formation of [**2 b**]^+^ as indicated by intense brown‐yellow colourization and suitable NMR signatures, but even at low temperatures (−40 °C) progressive decomposition of putative [**2 b**]^+^ to several yet unidentified pale yellow to colourless follow‐up products is observed (see Supporting Information).

In summary we presented a stable cationic borole extending the series of true free boroles to cationic systems. The central C_4_B moiety featuring an NHC‐stabilized borenium cation within a cyclic 4 π‐electron system is both isoelectronic to and bears the same overall charge as the elusive cyclopentadienyl cation. However, the antiaromatic character is not drastically increased to levels expected for cyclopentadienyl cations. This work sheds light on the implications of imidazolium substituents on the (opto)‐electronic properties of boroles and highlights the impact of formally cationic boron‐bound substituents, particularly compared to traditional strong electron withdrawing groups such as ‐(C_6_F_5_). The accessibility of the reactive [C_4_B] moiety in the presented examples for small molecules such as CO and acetylenes highlight its exceptional reactivity stemming from a combination of the frontier π‐orbitals and a cationic charge.

## Experimental Section

**Crystallographic data**: Deposition numbers 1982736, 1982737, and 1986857 contain the supplementary crystallographic data for this paper. These data are provided free of charge by the joint Cambridge Crystallographic Data Centre and Fachinformationszentrum Karlsruhe Access Structures service.

## Conflict of interest

The authors declare no conflict of interest.

## Supporting information

As a service to our authors and readers, this journal provides supporting information supplied by the authors. Such materials are peer reviewed and may be re‐organized for online delivery, but are not copy‐edited or typeset. Technical support issues arising from supporting information (other than missing files) should be addressed to the authors.

Supplementary
