# Supplementary material for: A Cationic NHC‐Supported Borole
Source: Chemistry. 2020 Aug 13;26(51):11684–9. doi: 10.1002/chem.202001916 (PMC7540045; doi:10.1002/chem.202001916)
Supplement: Supplementary file 1 — Supplementary [file CHEM-26-11684-s001.pdf]

Chemistry–A European Journal

Supporting Information

A Cationic NHC-Supported Borole

Tobias Heitkemper and Christian P. Sindlinger*[a]

Table of Contents

Experimental Details 3
General Information 3
UVVis and IR spectroscopy 3
Mass spectrometry 3
NMR spectroscopy 3
Gutmann-Beckett Analysis 3
Starting materials and reagents 4
Synthesis and Analytical Data 5
Compound 1a 5
Analytical Data for Compound 1a 5
Crystal structure of Compound 1a 6
Spectra Plots for Compound 1a 7
Compound 1b 10
Analytical Data for Compound 1b 10
Spectra Plots for Compound 1b 11
Compound [2a][Al{OC(CF_3)$_3$}$_4$] 14
Analytical Data for Compound [2a][Al{OC(CF_3)$_3$}$_4$] 14
Spectra Plots for Compound [2a][Al{OC(CF_3)$_3$}$_4$] 15
Spectra Plots for Compound [2a($OPEt_3$)][Al{OC(CF_3)$_3$}$_4$] (Gutmann-Becket Analysis) 19
Compound [3][Al{OC(CF_3)$_3$}$_4$] 22
Analytical Data for Compound [4][Al{OC(CF_3)$_3$}$_4$] 22
Spectra Plots for Compound [3][Al{OC(CF_3)$_3$}$_4$] 23
Compound [4][Al{OC(CF_3)$_3$}$_4$] 27
Analytical Data for Compound [4][Al{OC(CF_3)$_3$}$_4$] 27
Crystal structure of Compound [4][Al{OC(CF_3)$_3$}$_4$] 28
Spectra Plots for Compound [4][Al{OC(CF_3)$_3$}$_4$] 29
Crystallographic Details 32
General Data Acquisition and Processing 32
Crystallographic and Refinement Details 1 32
Crystallographic and Refinement Details [2a][Al{OC(CF_3)$_3$}$_4$] 32
Crystallographic and Refinement Details [4][Al{OC(CF_3)$_3$}$_4$] 32

Tabulated Crystallographic Details 1 33
Cyclovoltammetry 34
General Data Acquisition and Processing 34
Computational Details 36
Structure Optimisation, Frequency Calculation and Thermochemical Approximations 36
Computational Structure of $[4]^{+}$ 36
Computational Structure of $[4]^{+}$ 37
ETS-NOCV 37
Computational assessment of NMR spectroscopic features 37
Computational assessment of UVVis spectroscopic features 39
NICS Profiles 39
XYZ-coordinates of optimised structures 41
Literature 45

Experimental Details

General Information

All manipulations requiring handling under inert conditions were carried out under argon atmosphere using standard Schlenk techniques or an MBraun Glovebox with an Ar atmosphere. Benzene was obtained from an MBraun SPS and stored over molecular sieves, toluene and ether were distilled from sodium. Dichloromethane was distilled from CaH_2. Hexane and pentane were distilled from Na/K alloy. THF was distilled from potassium. Dichloromethane-d_2 was distilled from CaH_2, benzene-d_6 was distilled from potassium, and solvents were degassed and stored in a glove box. All solvents were routinely degassed three times using freeze-pump-thaw cycles.

Elemental analysis was performed by the Analytisches Labor, Institut für Anorganische Chemie, Universität Göttingen

UVVis and IR spectroscopy

UV/Vis spectra were recorded on an Agilent Cary 60 or an Agilent Cary 50 spectrometer using quartz cuvettes fitted with Young-type teflon-valves in dichloromethane. Absorption maxima of **A-Mes** were found identical in pentane and dichloromethane, thus allowing direct comparison. IR spectra were recorded on a CARY630 FTIR spectrometer with an ATR unit for liquid and solid samples fitted in glovebox. Fresh solutions of [**4**][Al{O(C(CF_3)$_3$}$_4$] in dichloromethane were applied and after a few seconds the dichloromethane evaporated and IR spectra revealed only signatures of [**4**][Al{O(C(CF_3)$_3$}$_4$].

Mass spectrometry

Mass spectra were recorded by the Zentrale Analytik within the Faculty of Chemistry, Göttingen applying a Liquid Injection Field Desorption Ionisation-technique on a JEOL accuTOF instrument with an inert-sample application setup under argon atmosphere. The injection capillary was washed several times with dry, distilled and inertly injected toluene before the samples were injected. Samples usually had a concentration of 1 – 2 mmol/L in toluene with a minimum amount of fluorobenzene added to guarantee solubility and were prepared in a glovebox.

NMR spectroscopy

NMR spectra were recorded with either a Bruker Avance III 400 NMR spectrometer equipped with a 5 mm BBFO ATM probe head and operating at 400.13 (^{1}H), 100.61 (^{13}C), 128.38 (^{11}B) and 376.45 MHz (^{19}F) along with a variable temperature set-up or a Bruker Avance Neo 400 NMR spectrometer with a CryoProbeProdigy BB ATM probe head operating at 400.25 MHz (^{1}H). Chemical shifts are reported in δ values in ppm relative to external Me_4Si and, if not otherwise stated, referenced using the chemical shift of the solvent ^{2}H lock resonance frequency and Ξ = 19.867187 for ^{29}Si, Ξ = 32.083974% for ^{11}B, and Ξ = 94.094011 % for ^{19}F.[1] ^{15}N-NMR chemical shifts were obtained from ^{1}H-^{15}N HMBC experiments. ^{1}H and ^{13}C spectra have been referenced on specific values for the respective solvent signal. The proton and carbon signals were assigned where possible via a detailed analysis of ^{1}H, ^{13}C, ^{1}H-^{1}H COSY, ^{1}H-^{1}H NOESY, ^{1}H-^{13}C HSQC, ^{1}H-^{13}C HMBC NMR spectra.

Young-type teflon-valve borosilicate NMR tubes have been used throughout the study.

Gutmann-Beckett Analysis

Assessments of the Lewis-acidity of the presented borole derivatives were studied using the Gutmann-Beckett method, analogously to a previously reported assessment of pentaphenyl borole and our recent report on 2,5-disilylboroles.[2] To allow for unambiguous comparison the Gutmann-Beckett analysis was performed for [**2a**][Al{OC(CF_3)$_3$}$_4$] and **A-Mes** in pure CD_2Cl_2 as well as C_6D_6 with a minimum amount of CD_2Cl_2 to guarantee solubility of [**2a**][Al{OC(CF_3)$_3$}$_4$]. The ^{31}P-NMR shifts obtained for [**2a**][Al{OC(CF_3)$_3$}$_4$] were 83.4 ppm (CD_2Cl_2) and 81.1 ppm (C_6D_6 + CD_2Cl_2); and for **A-Mes** were 50.4 ppm ((CD_2Cl_2) and 46.1 ppm (C_6D_6). The values observed for **A-Mes** basically do not differ from those of pure $OPEt_3$ in the respective solvents. In each case 0.5 equivalents of Et_3P=O (stock solution in C_6D_6 or CD_2Cl_2 respectively) was consecutively added three times to samples

of the boroles or borole-cations with an Eppendorf-Pipette and ^{31}P-NMR chemical shifts were determined after each addition. The observed shifts for the adduct were in each case identical. The acceptor numbers (AN) were calculated according to $2.21\times(\delta_{measured} - 41)$.[3]

Starting materials and reagents

Et_3PO was obtained from ABCR, Germany.

Tolane and $LiAlH_4$ (1 M in Et_2O) was obtained from Sigma Aldrich

Perfluoro-*t*-butanol was obtained from Fluorochem UK.

Chloroborole **A-Cl** and mesitylborole **A-Mes** were prepared as reported previously.[2b]

$Li[Al\{OC(CF_3)_3\}_4]$ was prepared according to the literature.[4]

$[NBu_4][Al\{OC(CF_3)_3\}_4]$ for electrochemical application was prepared according to the literature.[5]

^{Me}NHC and ^{iPr}NHC were prepared according to the literature.[6]

CO was obtained from a cylinder and taken from a reservoir kept at –78°C for a few hours to trap trace contamination of moisture.

Synthesis and Analytical Data

Compound 1a

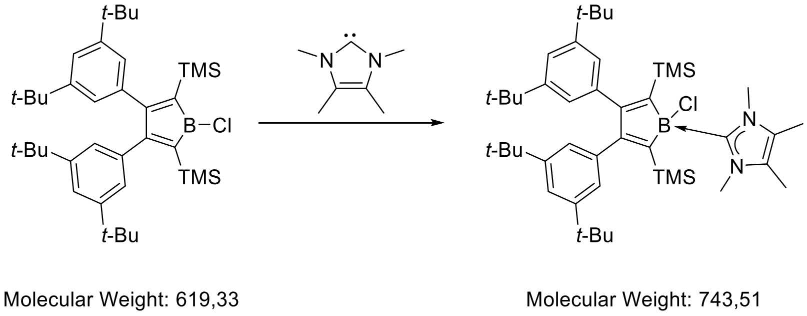

In a glovebox, chloroborole **A-Cl** (399 mg, 0.645 mmol, 1 eq) was suspended in dry, degassed toluene (6 mL). Me_4NHC (80 mg, 0.645 mmol, 1 eq) was added to the solution. No change in colour was observed upon addition of the NHC and after a few minutes the residual solid chloroborole **A-Cl** was completely dissolved. The reaction mixture was stirred at ambient temperature overnight. During the reaction, a small amount of fine red precipitate of yet unknown identity had formed, which was removed by filtration through a syringe filter equipped with a thin plug of glass fiber. The filter was washed with pentane (2 x 0.5 mL) to give a yellow filtrate and a red filter cake. The solvents of the filtrate were removed under reduced pressure to yield a yellow solid. Dissolving this solid in pentane/toluene (2:1) and storage at –40 °C overnight yielded colourless crystals of the title compound. The mother liquor was decanted with a syringe and the crystals were washed with cold pentane (2 x 0.5 mL). After a second crystallization step, the combined crystals were dried in vacuo to yield compound **1a** (372 mg, 0.500 mmol, 78 %) as a colourless to pale yellow solid.

Note: By NMR, the filtrate and mother liquor of the crude reaction mixture only contain compound **1a**. However, these pale yellow solutions appear to be weakly fluorescent and crystallization for purification seems necessary. Samples of crystalised **1a** revealed no fluorescence.

Analytical Data for Compound 1a

NMR:

^{1}H (400.13 MHz, 299 K, C_6D_6, CD_5H at 7.15 ppm): 7.26 (t, $^4J_{HH}$ = 1.86 Hz, 2H, *p-H$_{ar}$*), 7.07 (br, 4H, *o-H$_{ar}$*), 3.91 (s, 3H, NC*H$_3$*), 3.45(s, 3H, NC*H$_3$*), 1.29 (br d, 3H, C=CC*H$_3$*), 1.28 (s, 36H, C(*Me*)$_3$), 1.24 (br d, 3H, C=CC*H$_3$*), 0.12 (s, 18H, Si(*Me*)$_3$).

^{13}C{^{1}H} (100.65 MHz, 298 K, C_6D_6, solvent signal at 128.0 ppm): 166.0 (borole-*C$_{3,4}$*), 161.3 (*C$_{Carbene}$*), 157.9 (borole-*C$_{2,5}$*), 149.1 (*m-C$_{ar}$*), 142.8 (*ipso-C$_{ar}$*), 125.1 (C=*C$_{NHC}$*), 124.2 (C=*C$_{NHC}$*), 123.9 (br, *o-C$_{ar}$*), 119.2 (*p-C$_{ar}$*), 34.7 (*C*(CH$_3$)$_3$), 34.1 (N$_{NHC}$-*C*H$_3$), 31.8 (C(*C*H$_3$)$_3$), 30.3 (N$_{NHC}$-*C*H$_3$), 8.2 (C$_{NHC}$-*C*H$_3$), 7.9 (C$_{NHC}$-*C*H$_3$), 1.2 (Si(*C*H$_3$)$_3$).

^{11}B (128.38 MHz ,299 K, C_6D_6): 0.5.

^{1}H-^{15}N-HMBC (40.56 MHz, 298 K, C_6D_6): –211.4, –207.1.

^{29}Si-INEPT (79.49 MHz, 300 K, C_6D_6): –11.0.

Elemental Analysis: $C_{45}H_{72}BClN_2Si_2$ calcd C 72.69, H 9.76, N 3.77; observed C 73.28, H 9.95, N 3.76.

LIFDI-MS: calcd exact mass: 742.50 m/z; observed m/z: 742.5 $[M]^+$.

Crystal structure of Compound 1a

1a crystallised from concentrated pentane/toluene solutions in a freezer (-40°C).

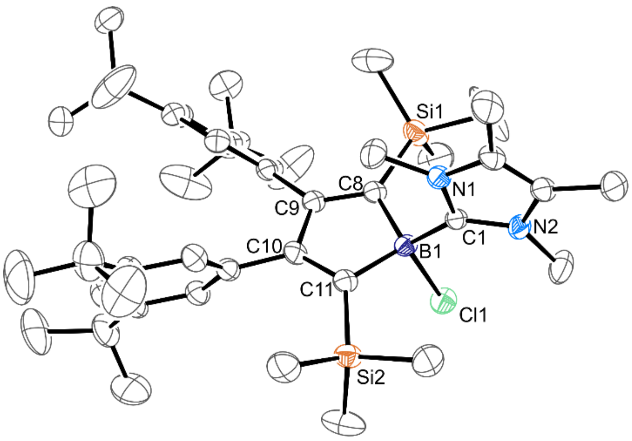

ORTEP plot of the molecular structure of **1a**. Atomic displacement parameters are drawn at 50% probability level. Disorder in *t*Bu groups and strongly disordered pentane lattice molecules are omitted for the sake of clarity. Selected bond length in Å: B1-C8 1.628(4), C8-C9 1.351(3), C9-C10 1.508(3), C10-C11 1.356(4), C11-B1 1.627(3), B1-Cl1 1.931(3), B1-C1 1.637(4).

Spectra Plots for Compound 1a

1H-NMR-spectrum of 1-Cl-2,5-(SiMe3)-3,4-(3',5'-tBu2C6H3)-borole * Me4NHC in C6D6 referenced to C6D5H at 7.15 ppm

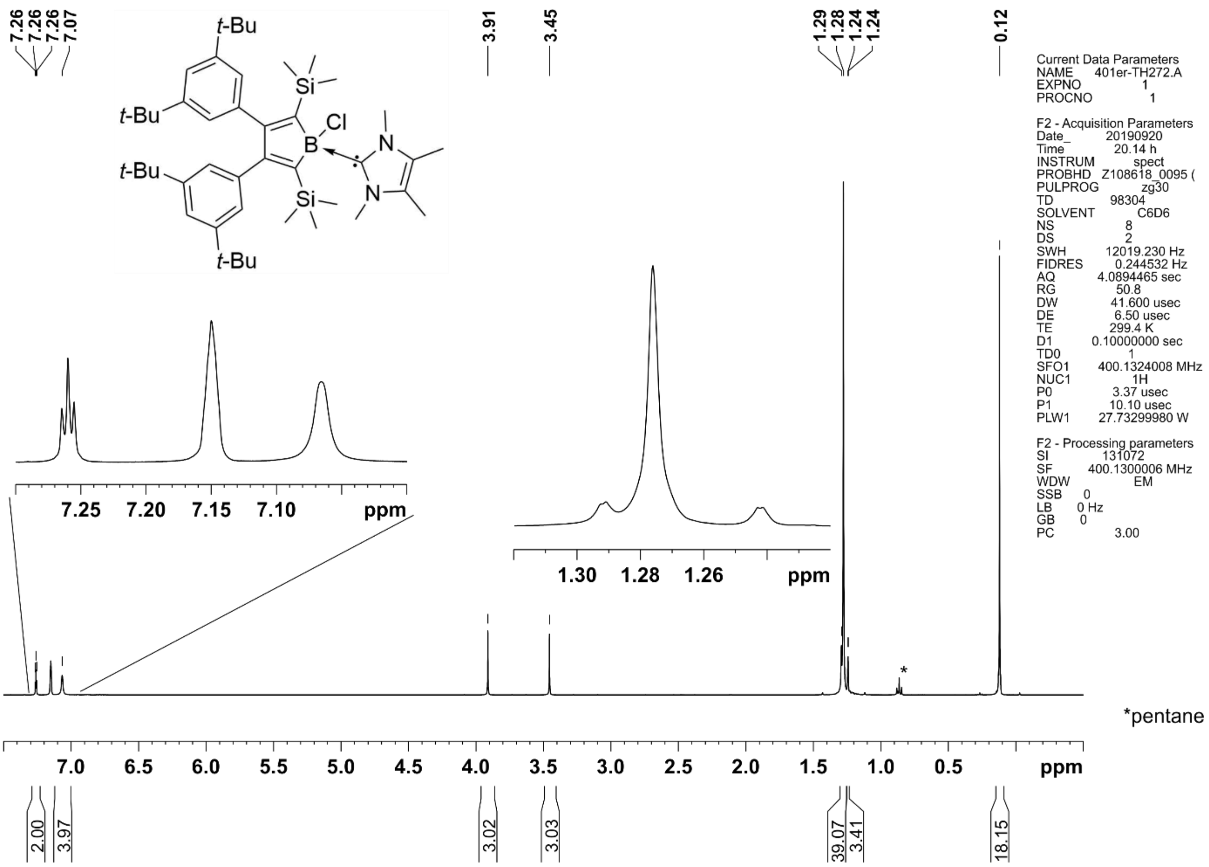

13C{1H}-NMR-spectrum of 1-Cl-2,5-(SiMe3)-3,4-(3',5'-tBu2C6H3)-borole * Me4NHC in C6D6 referenced to C6D6 at 128.0 ppm

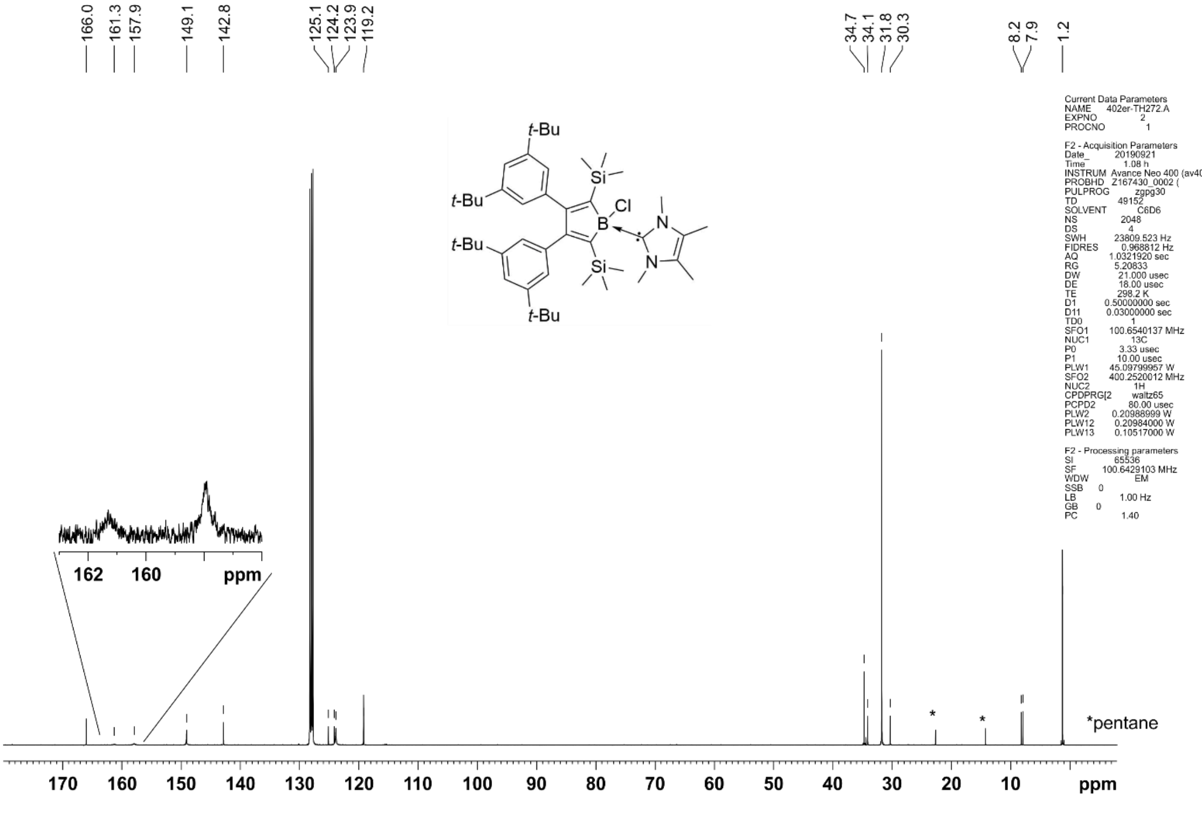

11B-NMR spectrum (background suppressed) of 1-Cl-2,5-(SiMe3)-3,4-(3',5'-tBu2C6H3)-borole * Me4NHC in C6D6

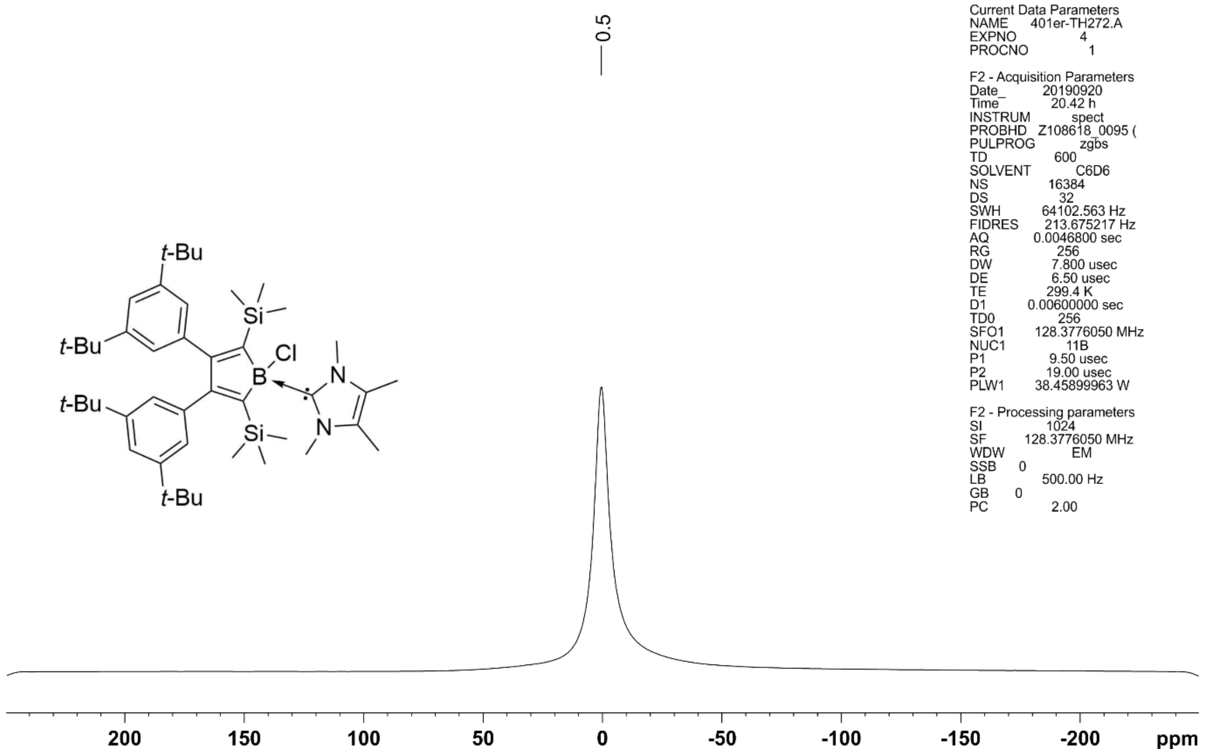

29Si-INEPT-NMR-spectrum of 1-Cl-2,5-(SiMe3)-3,4-(3',5'-tBu2C6H3)-borole * Me4NHC in C6D6

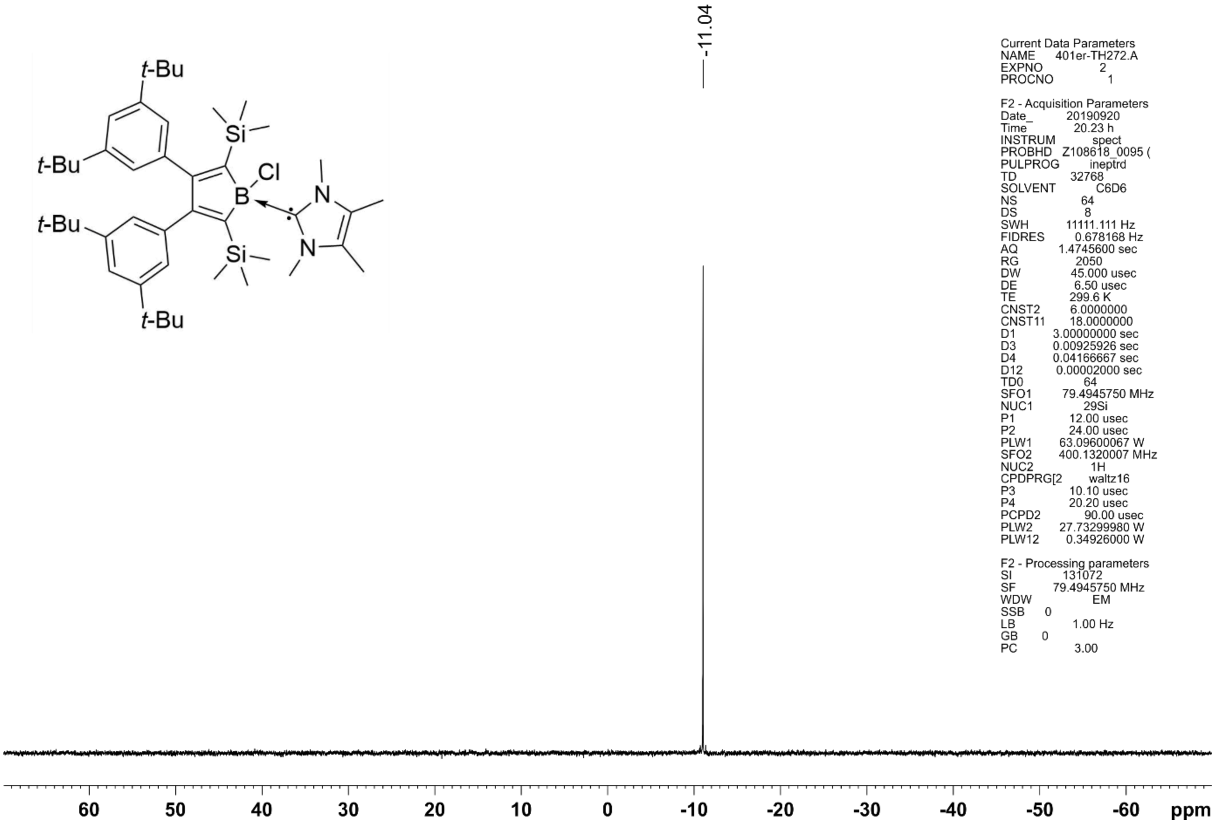

15N-HMBC NMR spectrum of 1-Cl-2,5-(SiMe3)-3,4-(3',5'-tBu2C6H3)-borole * Me4NHC in C6D6

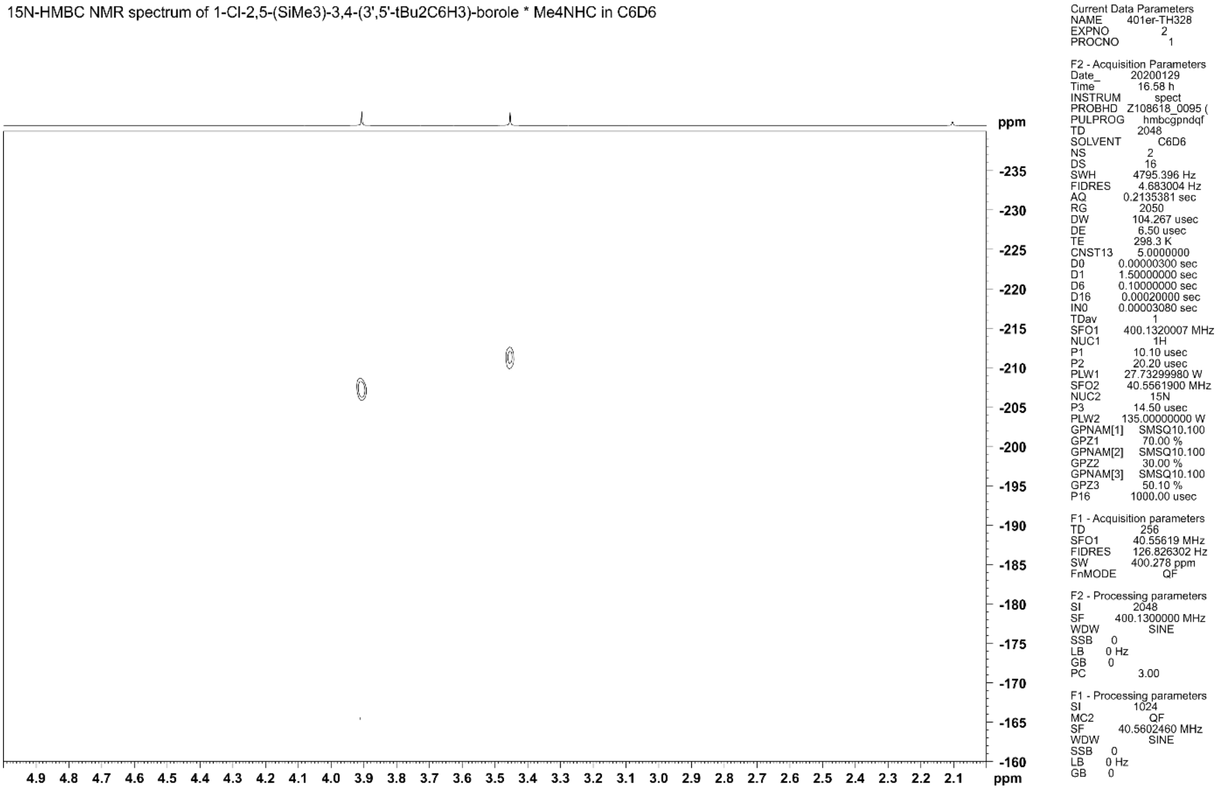

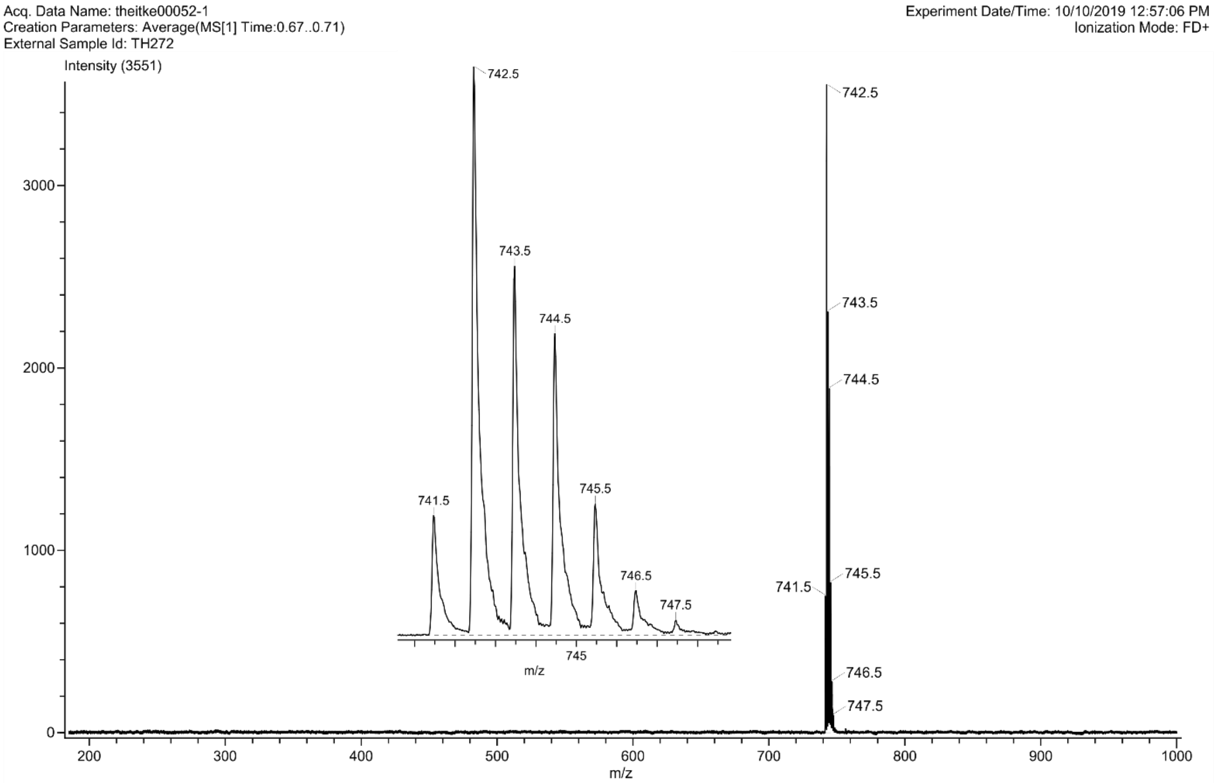

Compound 1b

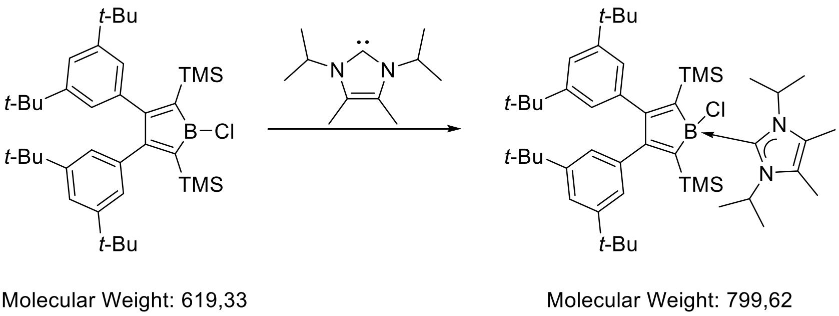

In a glovebox, chloroborole **A-Cl** (102.2 mg, 0.165 mmol, 1 eq) was suspended in dry, degassed pentane (7 mL). ^{iPr}NHC (29.8 mg, 0.165 mmol, 1 eq) was added to the solution. After a few minutes the suspended chloroborole **A-Cl** was completely dissolved and the solution turned yellow. The reaction mixture was stirred at ambient temperature for 2 h and was then stored at –40 °C overnight. Pale yellow crystals grew from this solution, which were isolated by decanting off the mother liquor with a syringe and drying in vacuo. Compound **1b** (91.3 mg, 114 mmol, 69 %) was isolated as a pale yellow solid.

Analytical Data for Compound 1b

NMR:

^{1}**H** (400.13 MHz, 299 K, C_6D_6, CD_5H at 7.15 ppm): 7.27 (sep, $^{3}J_{HH}$ = 7.02 Hz, 1H, C*H*(CH$_3$)$_2$), 7.22 (t, $^{4}J_{HH}$ = 1.86 Hz, 2H, *p-H$_{ar}$*), 7.18–7.00 (br, partially superimposed by solvent signal, 2H, *p-H$_{ar}$*), 7.00–6.77 (br, 2H, *p-H$_{ar}$*), 5.63 (sep, $^{3}J_{HH}$ = 7.02 Hz, 1H, C*H*(CH$_3$)$_2$), 1.62 (s, 3H, C=CC*H$_3$*), 1.61 (s, 3H, C=CC*H$_3$*), 1.38 (d, $^{3}J_{HH}$ = 7.02 Hz, CH(C*H$_3$*)$_2$), 1.34 (d, $^{3}J_{HH}$ = 7.02 Hz, CH(C*H$_3$*)$_2$), 1.26 (broad singulet, 36H, C(*Me*)$_3$), 0.17 (s, 18H, Si(*Me*)$_3$).

^{13}**C{^{1}H}** (100.65 MHz, 298 K, C_6D_6, solvent signal at 128.0 ppm): 165.4 (borole-*C$_{3,4}$*), 160.6 (*C$_{Carbene}$*), 159.9 (borole-*C$_{2,5}$*), 149. 4 (br, *m-C$_{ar}$*), 148.5 (br, *m-C$_{ar}$*), 142.8 (*ipso-C$_{ar}$*), 126.4 (C=*C$_{NHC}$*), 125.5 (C=*C$_{NHC}$*), 124.1 (br, *o-C$_{ar}$*), 119.0 (*p-C$_{ar}$*), 49.0 (*C*H(CH$_3$)$_2$), 48.2 (*C*H(CH$_3$)$_2$), 34.7 (*C*(CH$_3$)$_3$), 31.8 (C(*C*H$_3$)$_3$), 23.0 (CH(*C*H$_3$)$_2$), 21.9 (CH(*C*H$_3$)$_2$), 10.6 (C$_{NHC}$-*C*H$_3$), 10.1 (C$_{NHC}$-*C*H$_3$), 2.0 (Si(*C*H$_3$)$_3$).

^{11}**B** (128.38 MHz ,299 K, C_6D_6): 1.3

^{29}**Si-INEPT** (79.49 MHz, 300 K, C_6D_6): –10.9

Elemental Analysis: $C_{49}H_{80}BClN_2Si_2$ calcd C 73.60, H 10.08, N 3.50; observed C 73.41, H 9.97, N 3.46.

LIFDI-MS: calcd exact mass: 798.56 m/z; observed m/z: 798.5 [M]$^{+}$.

Spectra Plots for Compound 1b

1H-NMR-spectrum of 1-Cl-2,5-(SiMe3)-3,4-(3',5'-tBu2C6H3)-borole * iPr2Me2NHC in C6D6

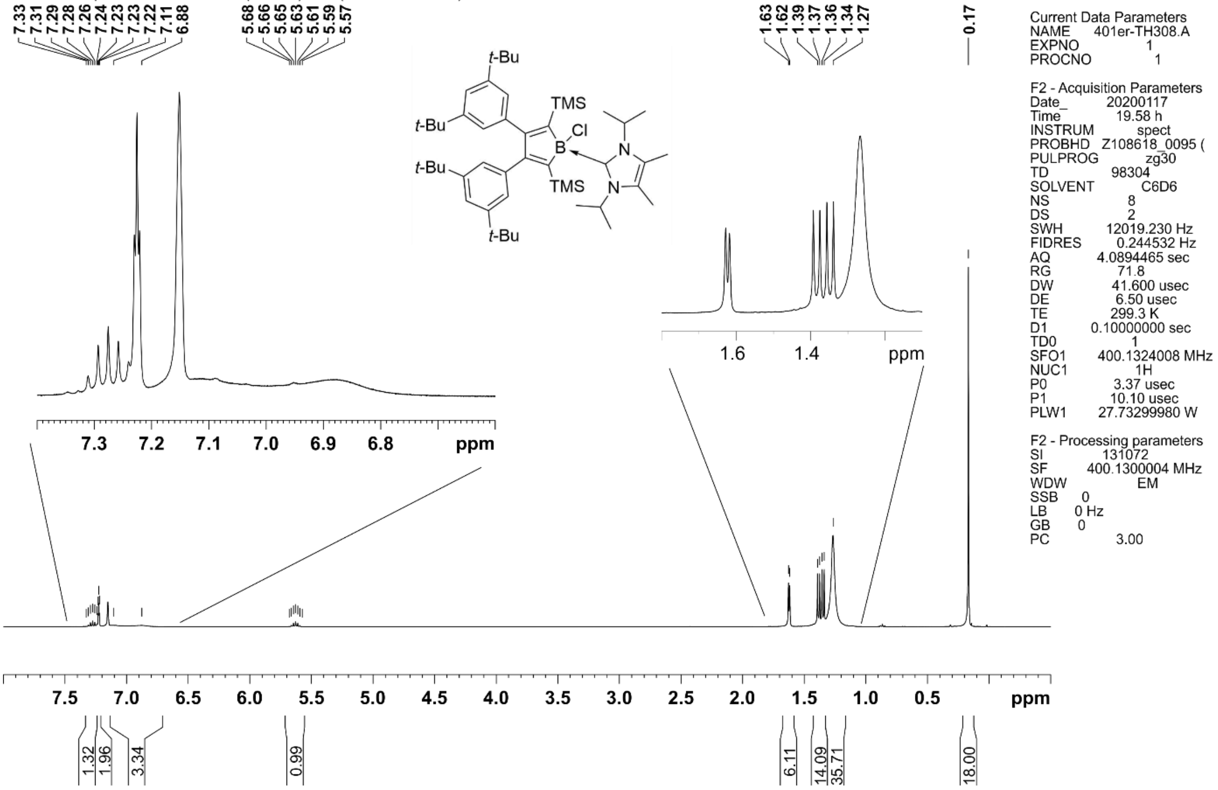

13C{1H}-NMR-spectrum of 1-Cl-2,5-(SiMe3)-3,4-(3',5'-tBu2C6H3)-borole * iPr2Me2NHC in C6D6

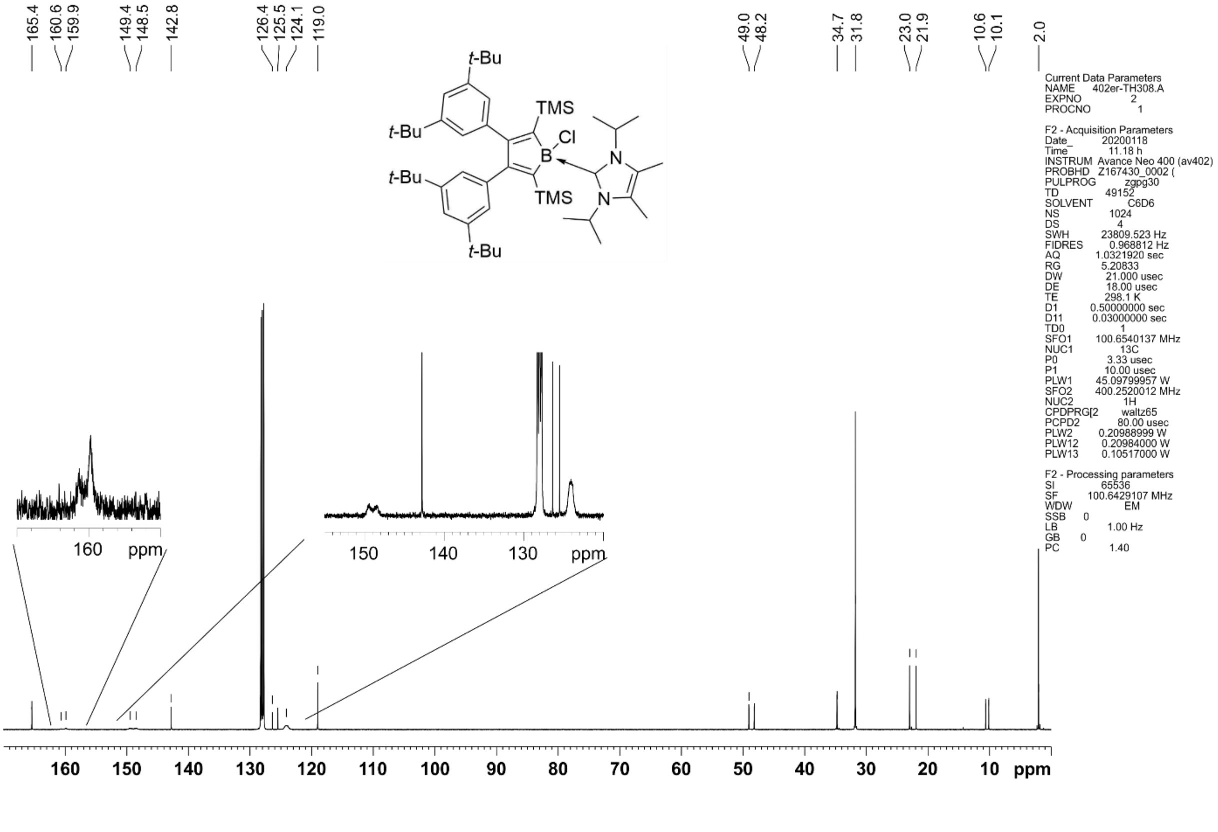

11B-NMR spectrum (background suppressed) of 1-Cl-2,5-(SiMe3)-3,4-(3',5'-tBu2C6H3)-borole * iPr2Me2NHC in C6D6

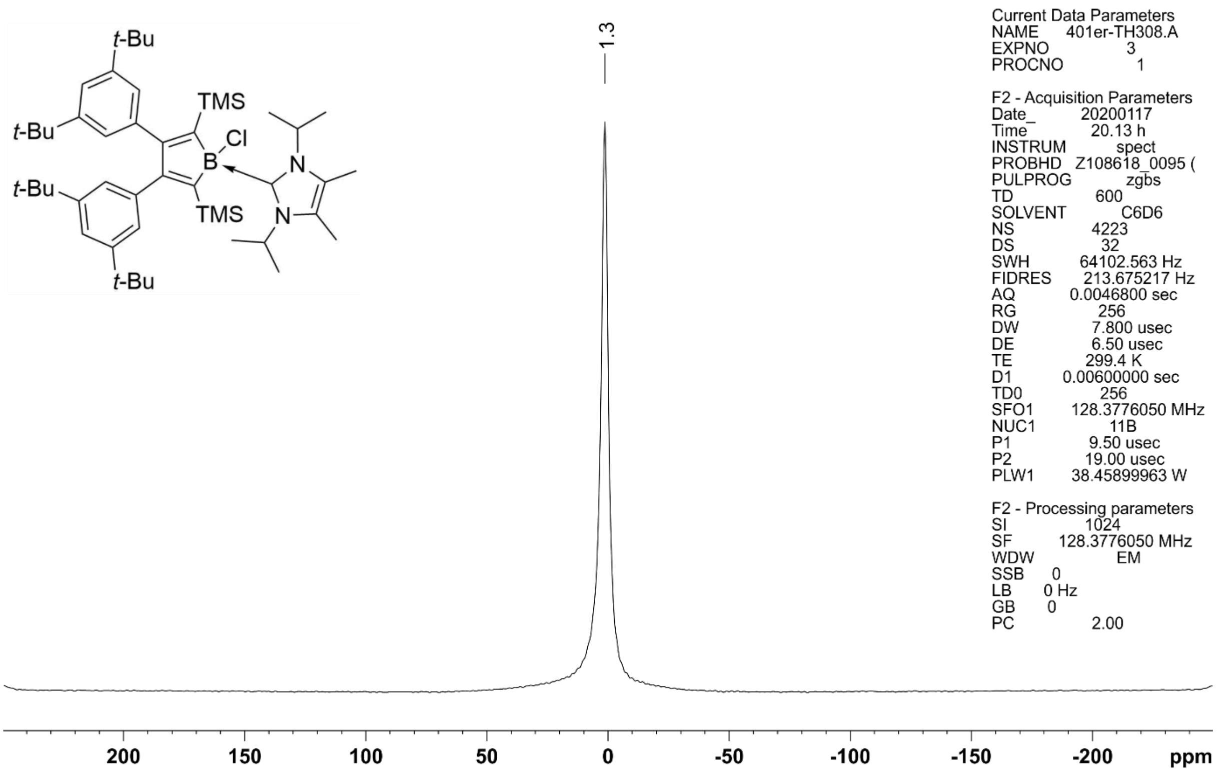

29Si-INEPT-NMR-spectrum of 1-Cl-2,5-(SiMe3)-3,4-(3',5'-tBu2C6H3)-borole * iPr2Me2NHC in C6D6

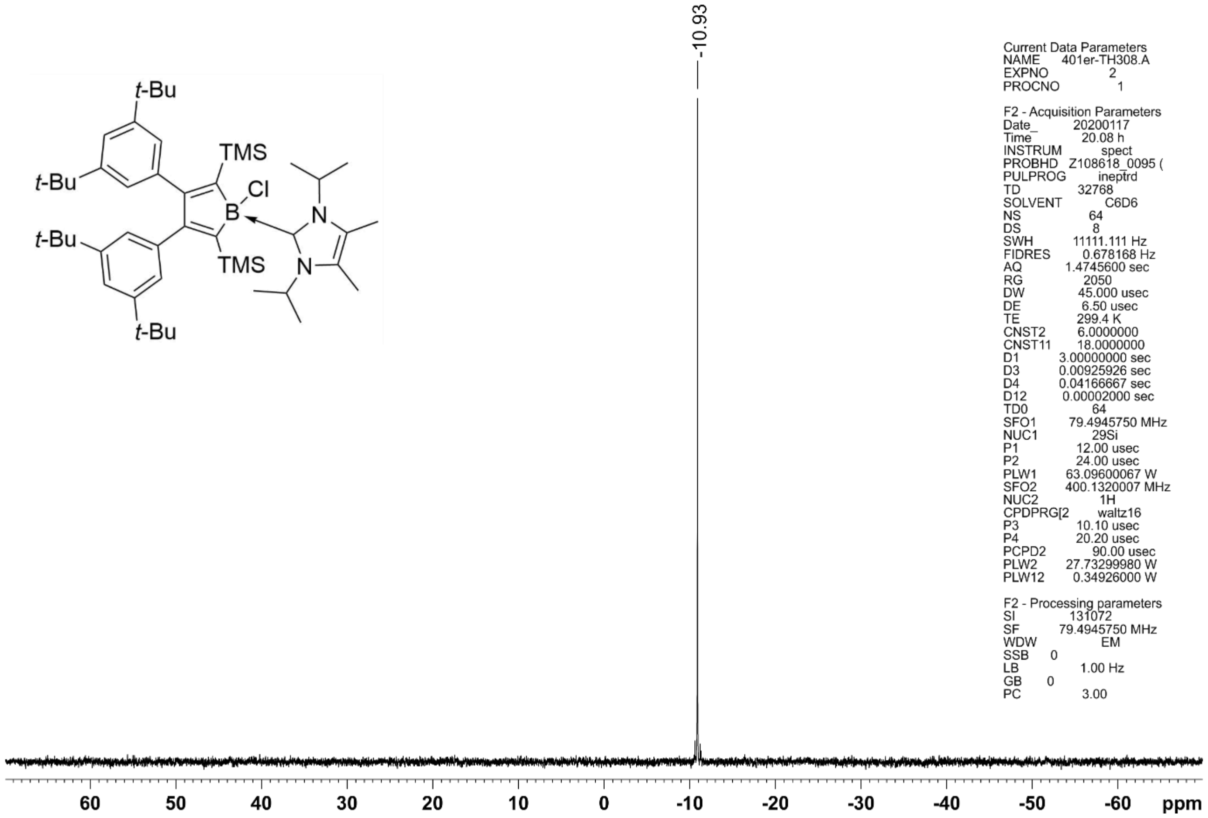

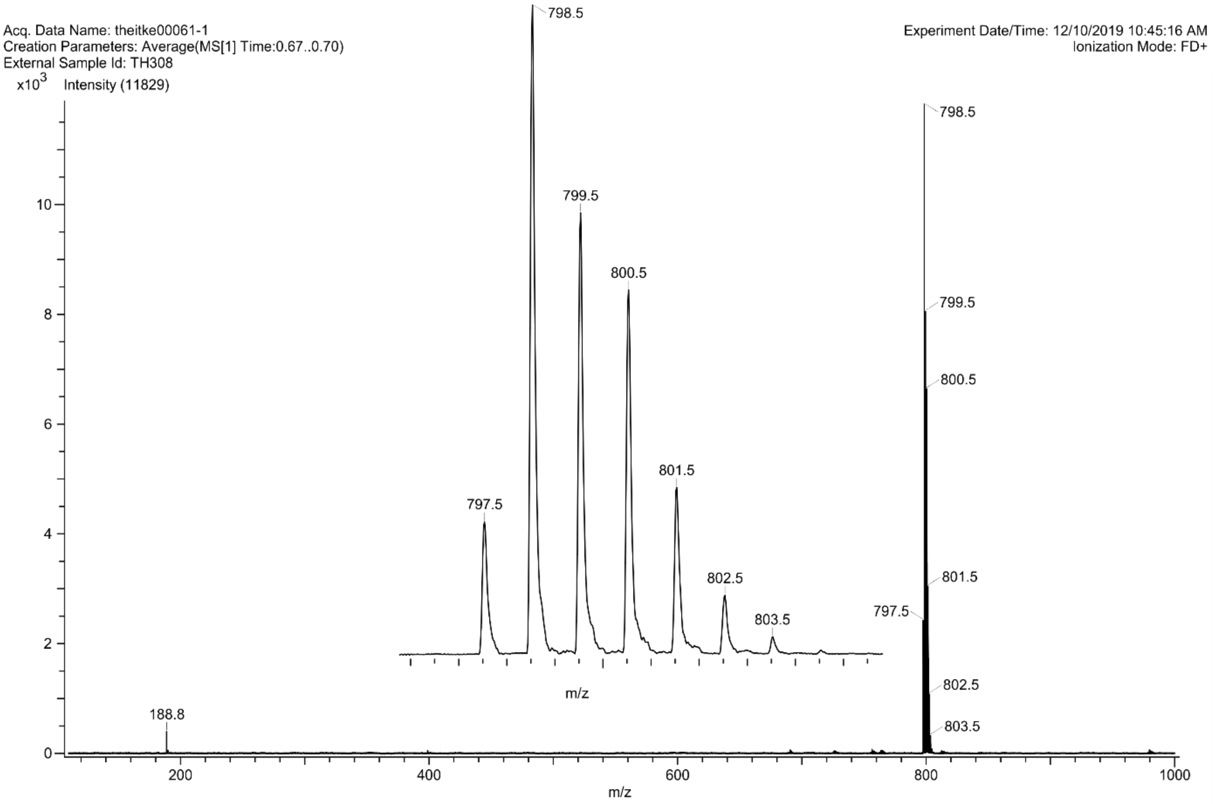
Acq. Data Name: theitke00061-1
Creation Parameters: Average(MS[1] Time:0.67..0.70)
External Sample Id: TH308
Experiment Date/Time: 12/10/2019 10:45:16 AM
Ionization Mode: FD+
x10^3 Intensity (11829)
798.5
799.5
800.5
801.5
797.5
802.5
803.5
188.8
m/z

Compound [2a][Al{OC(CF_3)$_3$}$_4$]

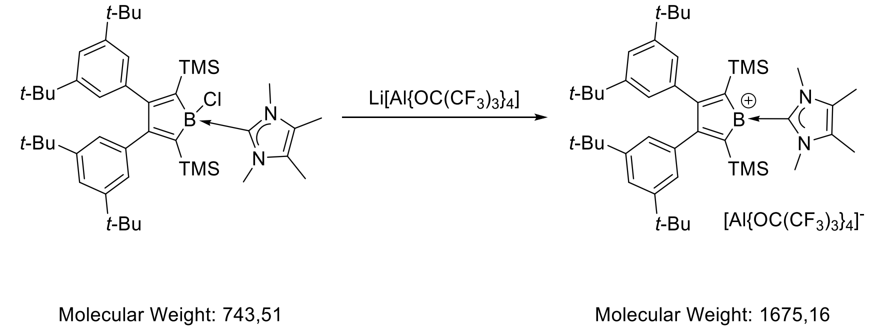

In a glovebox, compound **1a** (100 mg, 0.135 mmol, 1 equiv.) was dissolved in dry, degassed CH_2Cl_2. Li[Al{OC(CF_3)$_3$}$_4$] (131 mg, 0.135 mmol, 1 eq) was added and the solution instantly turned deep yellow. The reaction mixture was stirred for further 3.5 h at ambient temperature and was afterwards filtered through a syringe filter equipped with a thin plug of glass fiber. The filter was washed with CH_2Cl_2 (2 x 0.5 mL) and the solvent was removed under reduced pressure. The resulting dark yellow residue was dissolved in CH_2Cl_2 (1 mL), layered with pentane (3 mL) and stored at –40 °C overnight to yield dark yellow-green crystals. The mother liquor was decanted off and the crystals were washed with cold pentane (2 x 0.5 mL). Drying in vacuo yielded compound **[2a][Al{OC(CF_3)$_3$}$_4$]** (220 mg, 0.131 mmol, 98 %) as a dark yellow powder.

Analytical Data for Compound [2a][Al{OC(CF_3)$_3$}$_4$]

NMR:

^{1}H (400.13 MHz, 298 K, CD_2Cl_2, $CHDCl_2$ at 5.32 ppm): 7.33 (t, $^4J_{HH}$ = 1.80 Hz, 2H, *p-H$_{ar}$*), 6.67 (d, $^4J_{HH}$ = 1.80 Hz, 4H, *o-H$_{ar}$*), 3.77 (s, 6H, N$_{NHC}$-*Me*), 2.34 (s, 6H, C$_{NHC}$-*Me*), 1.14 (s, 36H, C(*Me*)$_3$), –0.31 (s, 18H, Si(*Me*)$_3$).

^{13}C{^{1}H} (100.65 MHz, 298 K, CD_2Cl_2, solvent signal at 53.8 ppm): 190.3 (borole-*C$_{3,4}$*), 150.4 (*C$_{Carbene}$*), 150.3 (*m-C$_{ar}$*), 137.4 (*ipso-C$_{ar}$*), 135.5 (borole-*C$_{2,5}$*), 129.7 (C=*C$_{NHC}$*), 124.1 (*p-C$_{ar}$*), 122.8 (*o-C$_{ar}$*), 121.7 (q, $^4J_{CF}$ = 292.3 Hz, *C*F$_3$), 35.0 (*C*(CH$_3$)$_3$), 34.6 (N$_{NHC}$-*C*H$_3$), 31.4 (C(*C*H$_3$)$_3$), 8.6 (C$_{NHC}$-*C*H$_3$), 0.2 (Si(*C*H$_3$)$_3$). Signals for the quaternary carbon atom of [Al{OC(CF_3)$_3$}$_4$]$^-$ were not observed.

^{11}B (128.38 MHz ,298 K, CD_2Cl_2): 73.9.

^{1}H-^{15}N-HMBC (40.56 MHz, 298 K, CD_2Cl_2): –209.9.

^{19}F{^{1}H} (376.45 MHz, 298 K, CD_2Cl_2): –75.70.

^{29}Si-INEPT (79.49 MHz, 300 K, CD_2Cl_2): –9.3.

Elemental Analysis: $C_{61}H_{72}AlBF_{36}N_2O_4Si_2$ calcd C 43.74, H 4.33, N 1.67; observed C 44.32, H 4.23, N 1.71.

LIFDI-MS (positive mode): calcd exact mass of the cation: 707.53 m/z; observed m/z: 707.6 [M]$^+$.

Spectra Plots for Compound [2a][Al{OC(CF$_3$)$_3$}$_4$]

1H-NMR-spectrum of 1-[Me4NHC]-2,5-[SiMe3]-3,4-(3', 5'-tBu2Ph)-borole Cation with [Al(OC(CF3)3)4] counteranion in CD2Cl2, referenced to CDHCl at 5.32 ppm

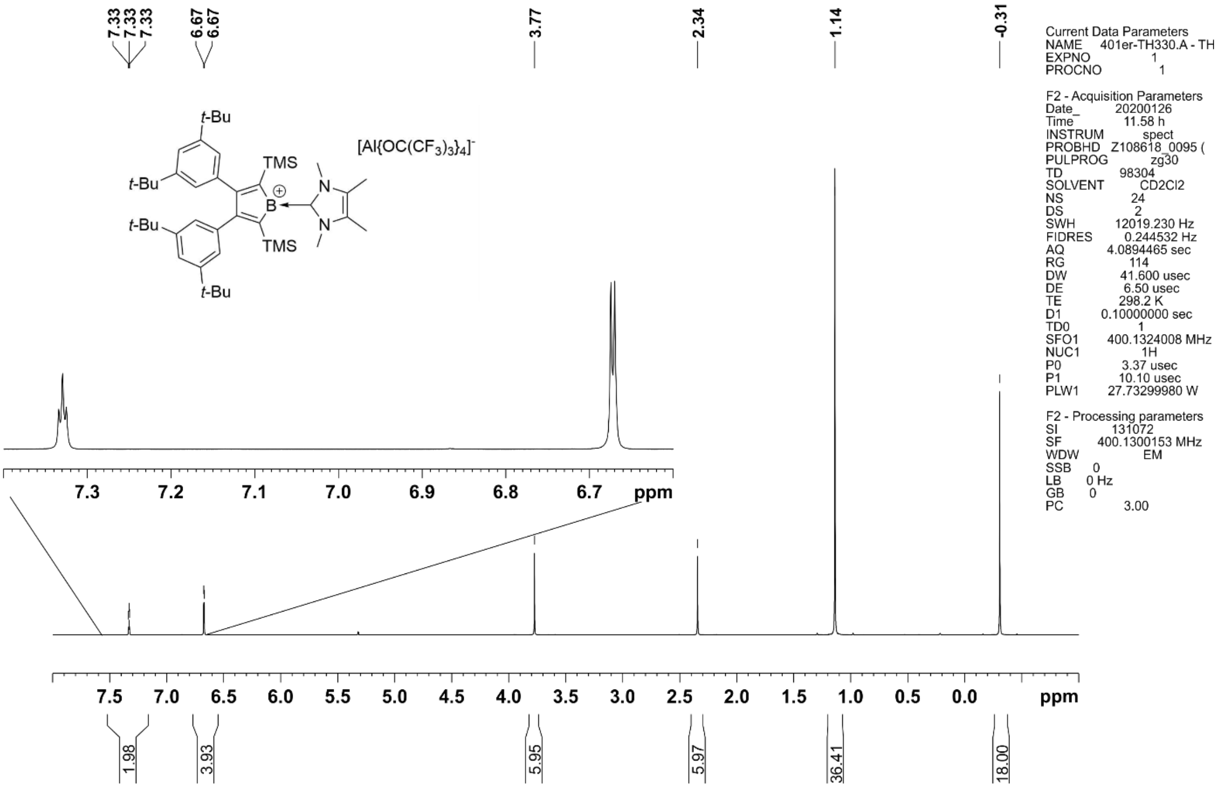

13C{1H}-NMR-spectrum of 1-[Me4NHC]-2,5-[SiMe3]-3,4-(3', 5'-tBu2Ph)-borole Cation with [Al(OC(CF3)3)4] counteranion in CD2Cl2

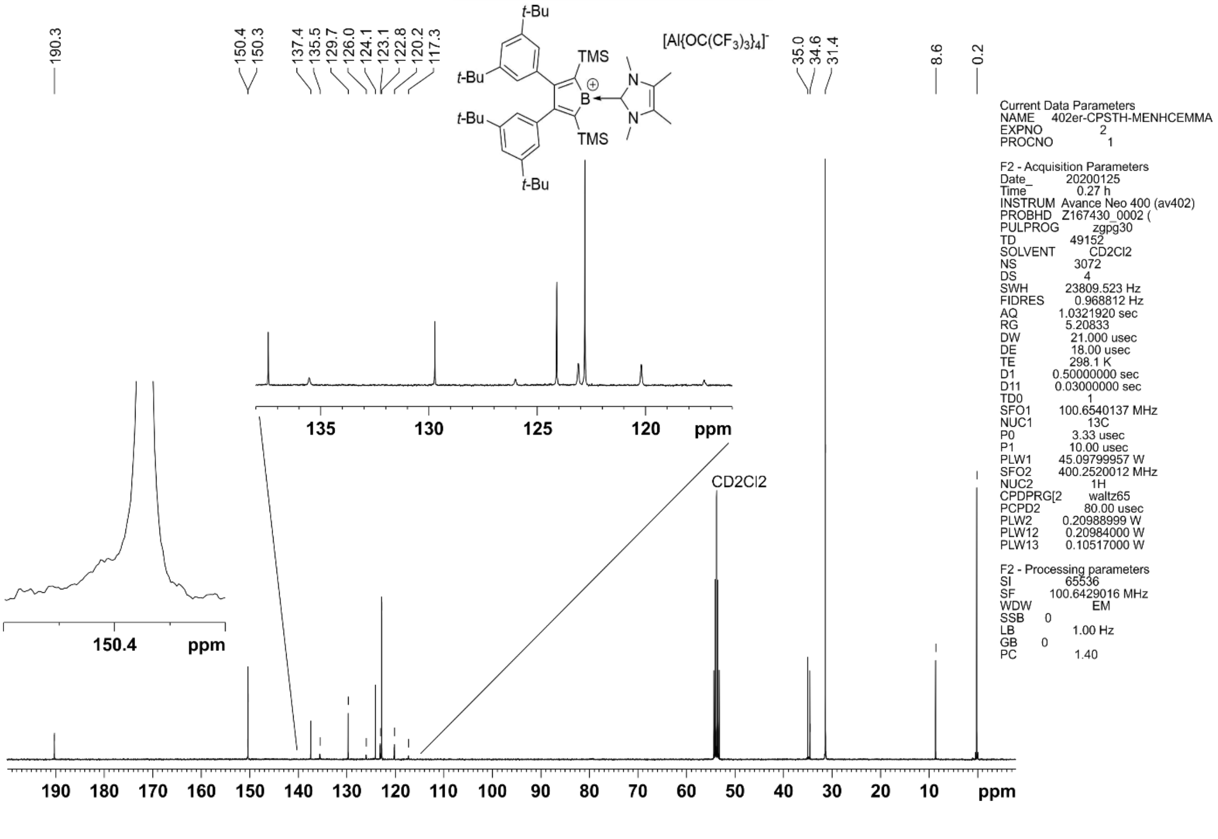

11B-NMR spectrum (background suppressed) of 1-[Me4NHC]-2,5-[SiMe3]-3,4-(3', 5'-tBu2Ph)-borole Cation with [Al(OC(CF3)3)4] counteranion in CD2Cl2

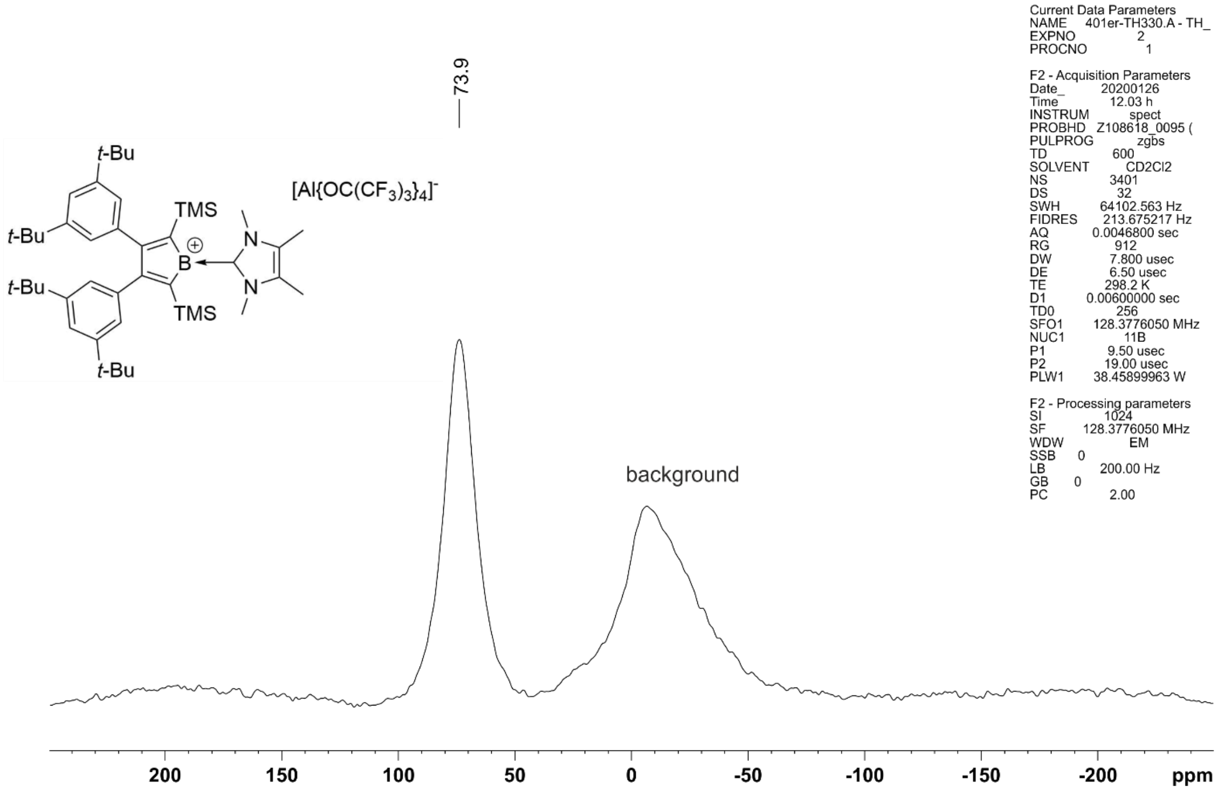

29Si-INEPT-NMR-spectrum of
1-[Me4NHC]-2,5-[SiMe3]-3,4-(3', 5'-tBu2Ph)-borole Cation with [Al(OC(CF3)3)4] counteranion in CD2Cl2

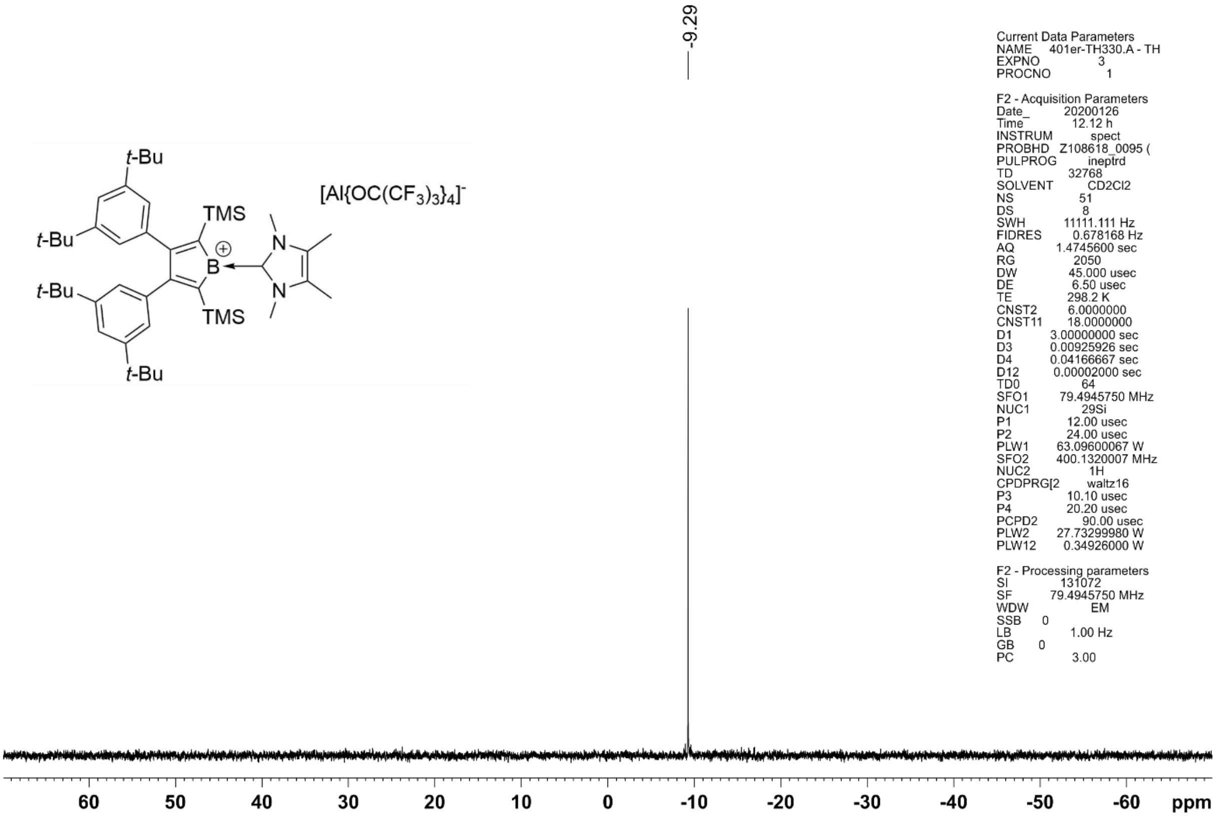

15N-HMBC NMR spectrum of 1-[Me4NHC]-2,5-[SiMe3]-3,4-(3', 5'-tBu2Ph)-borole Cation with [Al(OC(CF3)3)4] counteranion in CD2Cl2

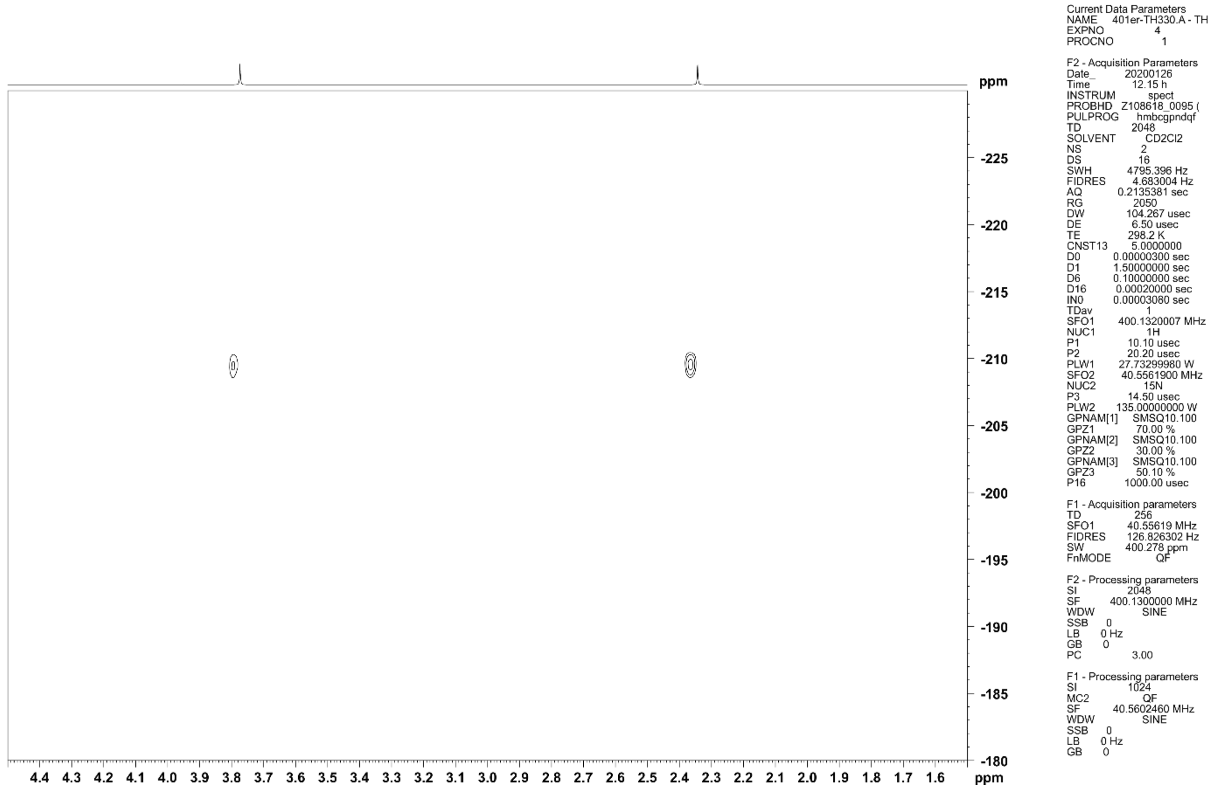

19F{1H}-NMR-spectrum 1-[Me4NHC]-2,5-[SiMe3]-3,4-(3', 5'-tBu2Ph)-borole Cation with [Al(OC(CF3)3)4] counteranion in CD2Cl2

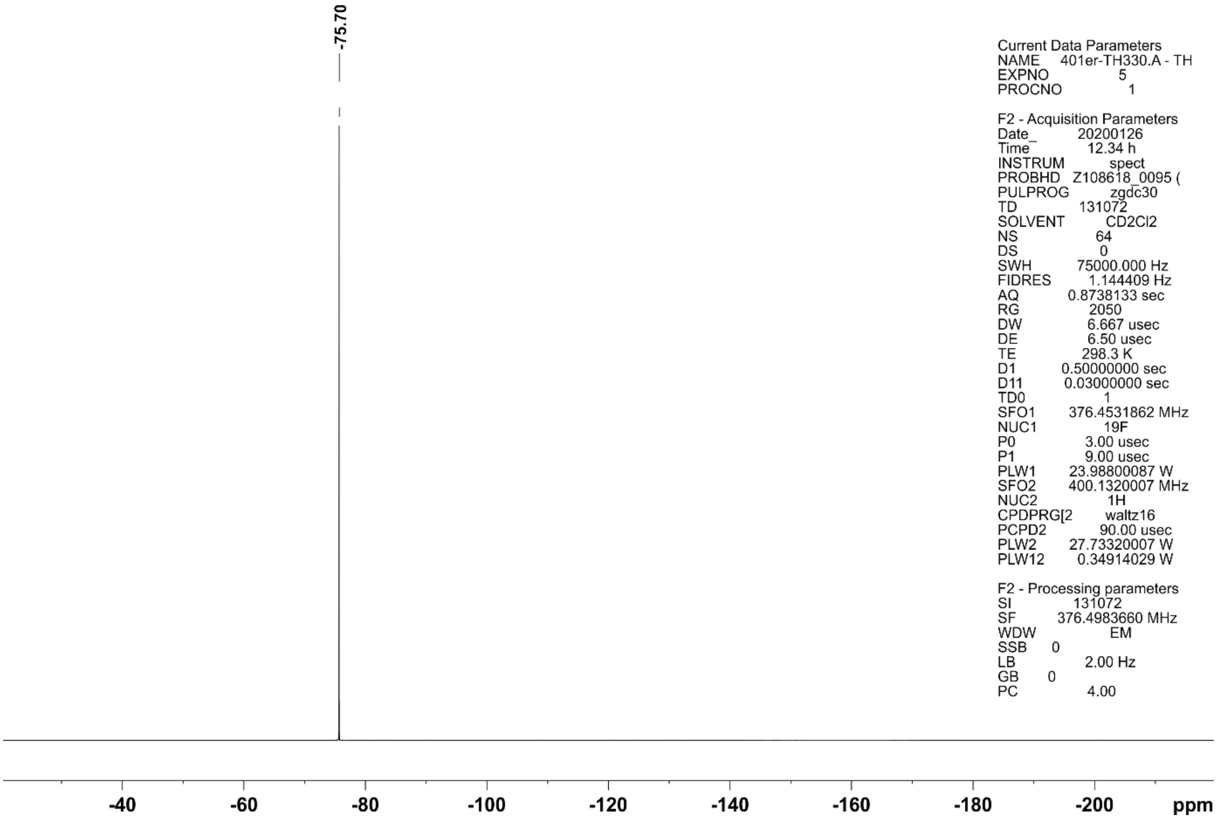

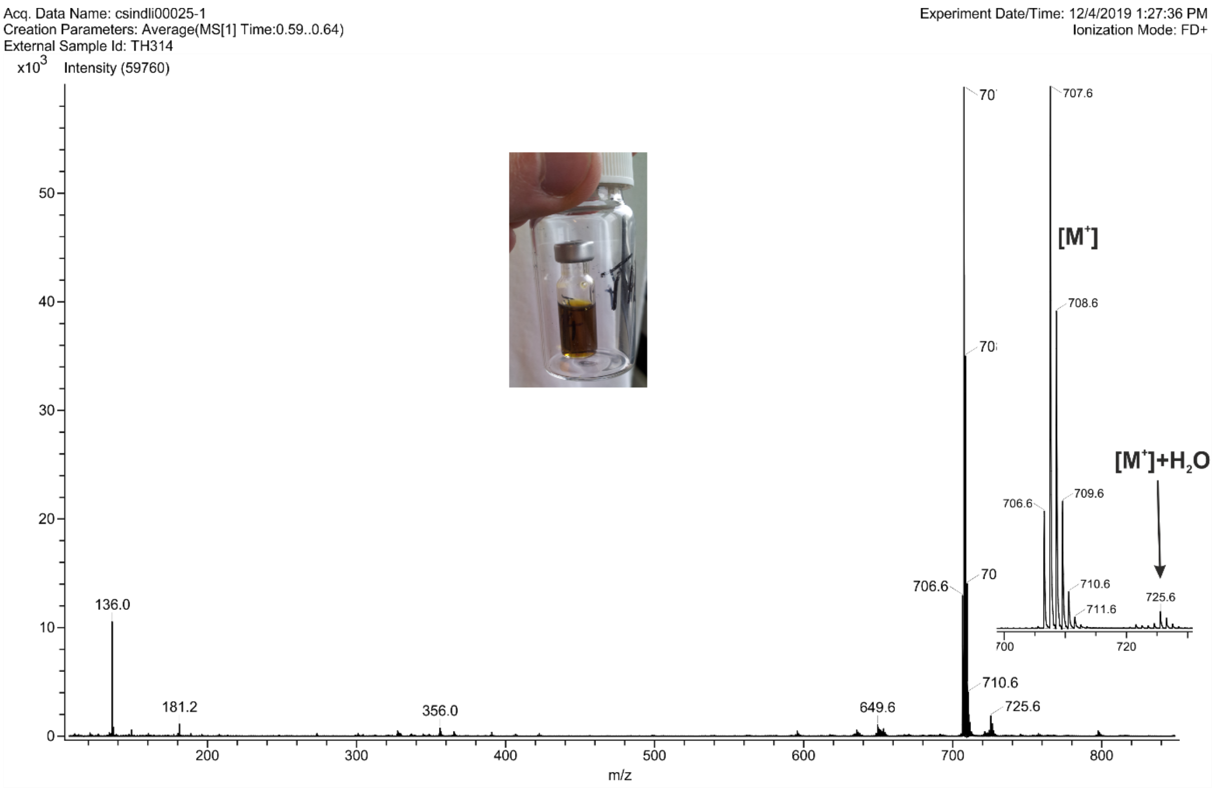
Acq. Data Name: csindli00025-1
Creation Parameters: Average(MS[1] Time:0.59..0.64)
External Sample Id: TH314
Experiment Date/Time: 12/4/2019 1:27:36 PM
Ionization Mode: FD+
x10^3 Intensity (59760)
m/z
136.0
181.2
356.0
649.6
706.6
710.6
725.6
[M+]
[M+]+H2O
707.6
708.6
706.6
709.6
710.6
711.6
725.6

Spectra Plots for Compound [2a(OPEt$_3$)][Al{OC(CF$_3$)$_3$}$_4$] (Gutmann-Becket Analysis)

1H-NMR-spectrum of 1-[Me4NHC]-2,5-[SiMe3]-3,4-(3', 5'-tBu2Ph)-borole * Et3PO with [Al(OC(CF3)3)4] counteranion in CD2Cl2

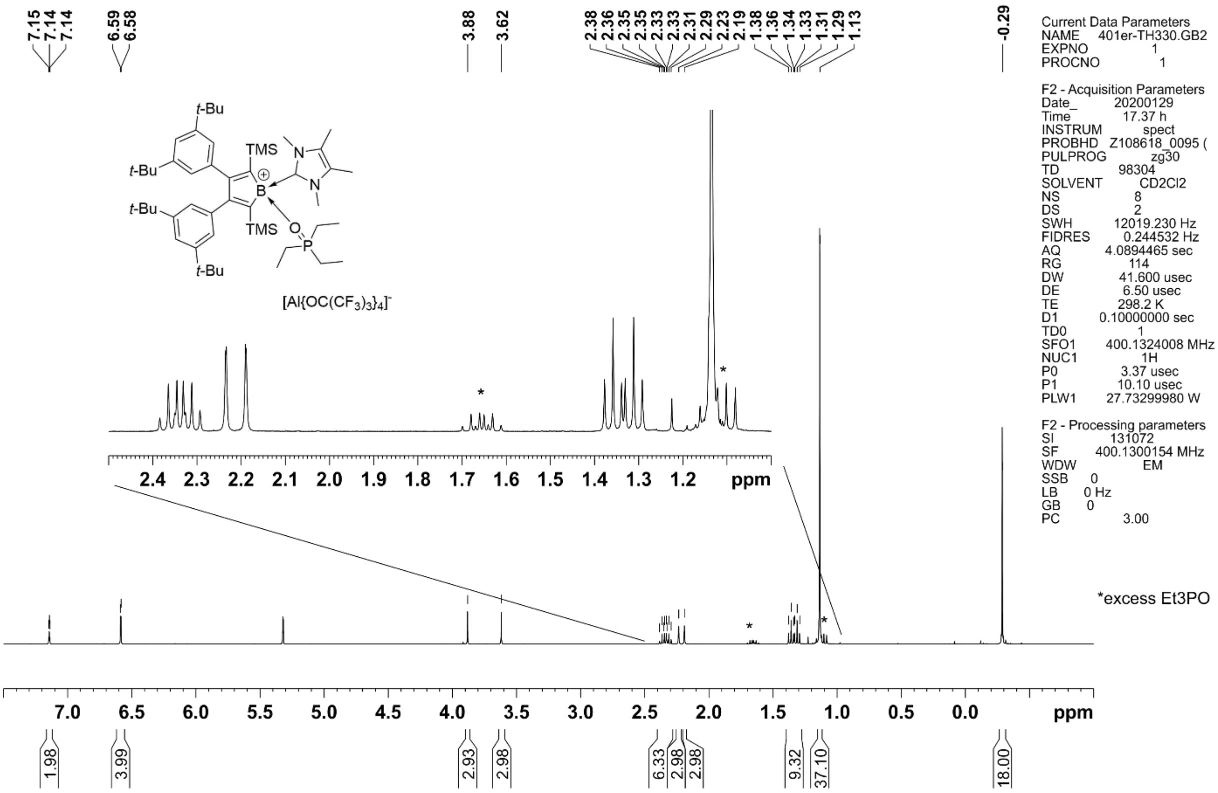

13C{1H}-NMR-spectrum of 1-[Me4NHC]-2,5-[SiMe3]-3,4-(3', 5'-tBu2Ph)-borole * Et3PO with [Al(OC(CF3)3)4] counteranion in CD2Cl2

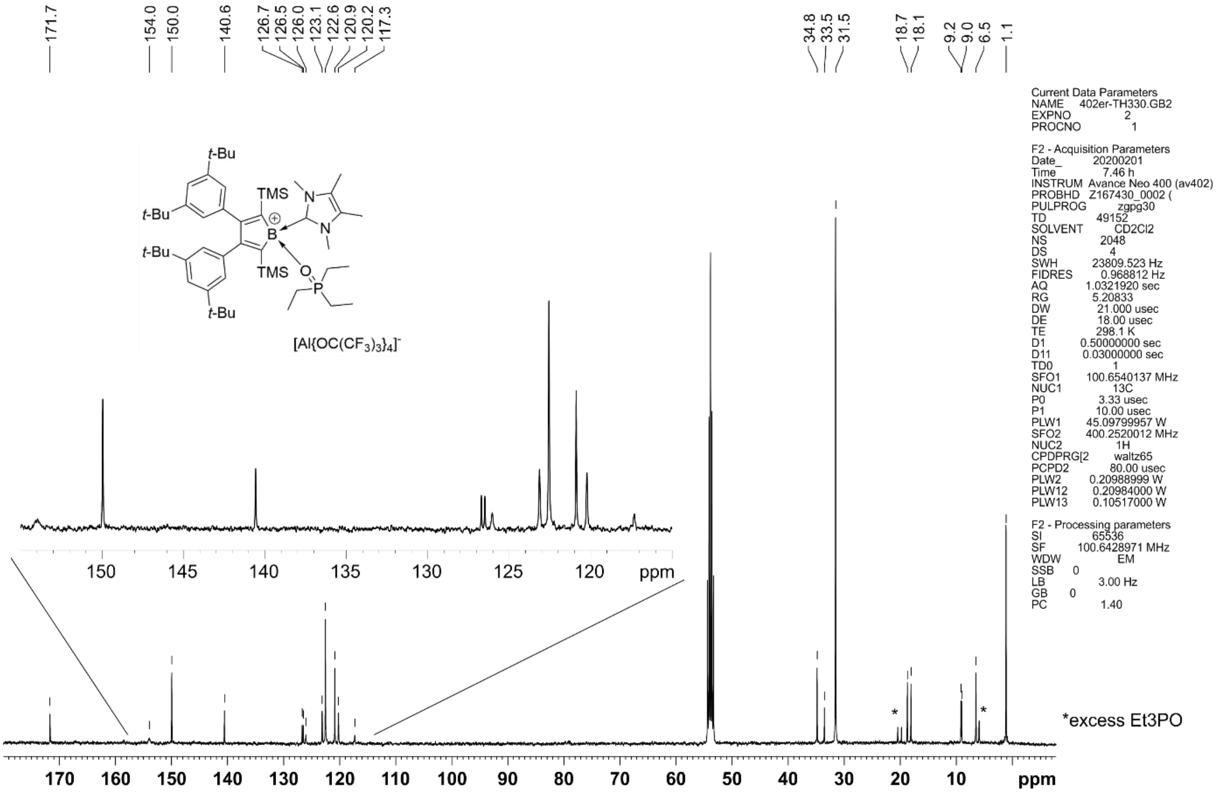

31P{1H}-NMR-spectrum of 1-[Me4NHC]-2,5-[SiMe3]-3,4-(3', 5'-tBu2Ph)-borole * Et3PO with [Al(OC(CF3)3)4] counteranion in CD2Cl2

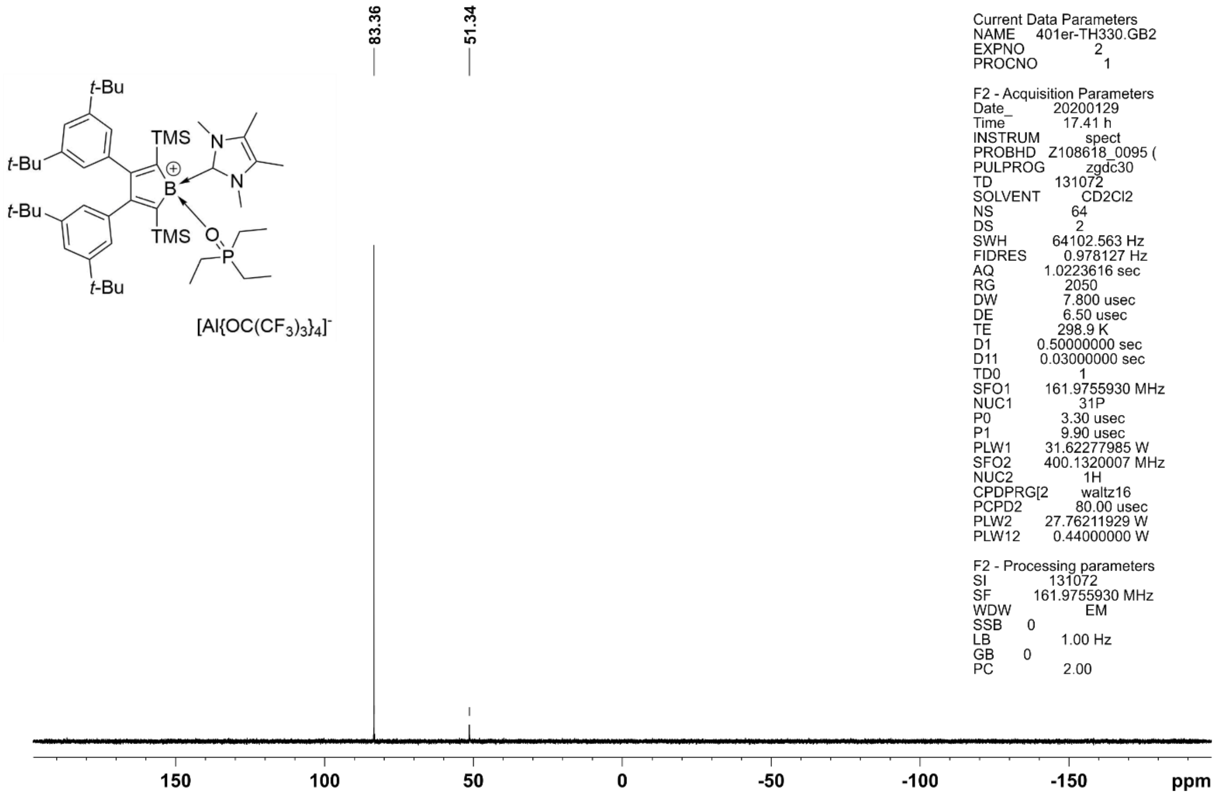

11B-NMR spectrum (background suppressed) of
1-[Me4NHC]-2,5-[SiMe3]-3,4-(3', 5'-tBu2Ph)-borole * Et3PO with [Al(OC(CF3)3)4] counteranion in CD2Cl2

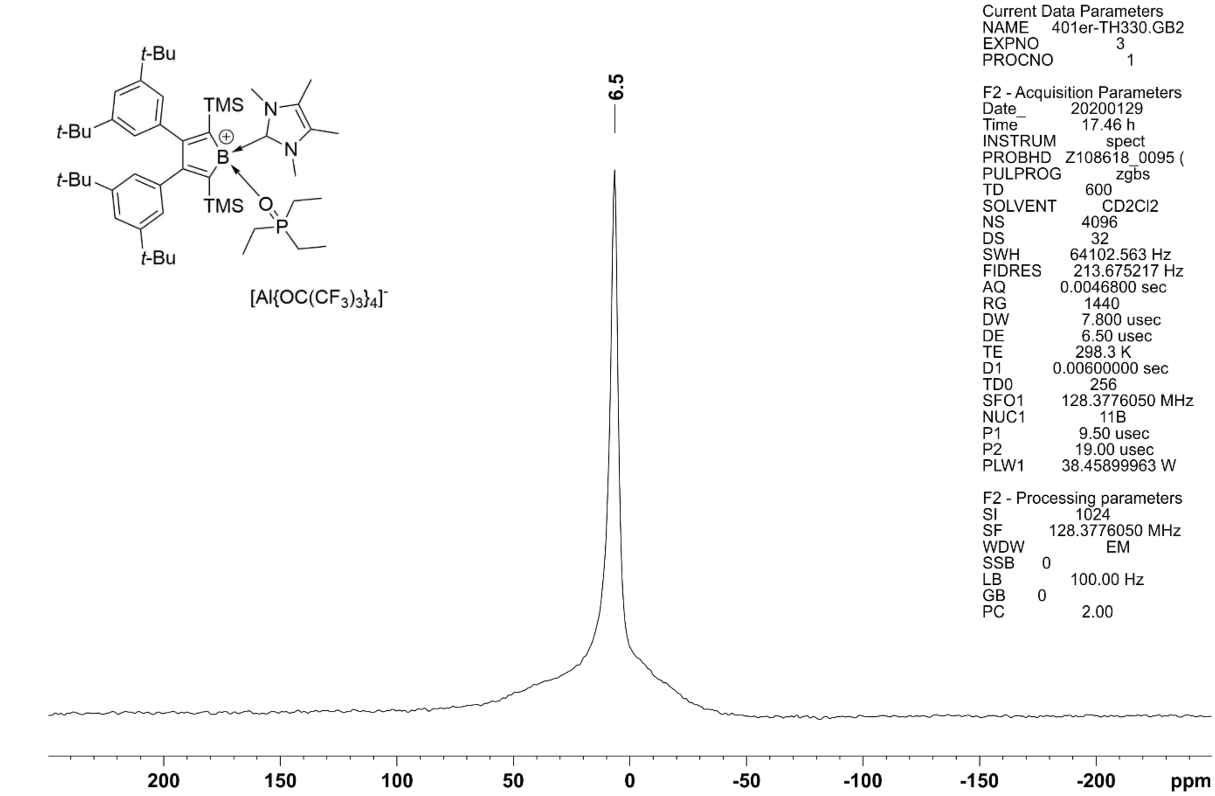

29Si-INEPT-NMR-spectrum of 1-[Me4NHC]-2,5-[SiMe3]-3,4-(3', 5'-tBu2Ph)-borole * Et3PO with [Al(OC(CF3)3)4] counteranion in CD2Cl2

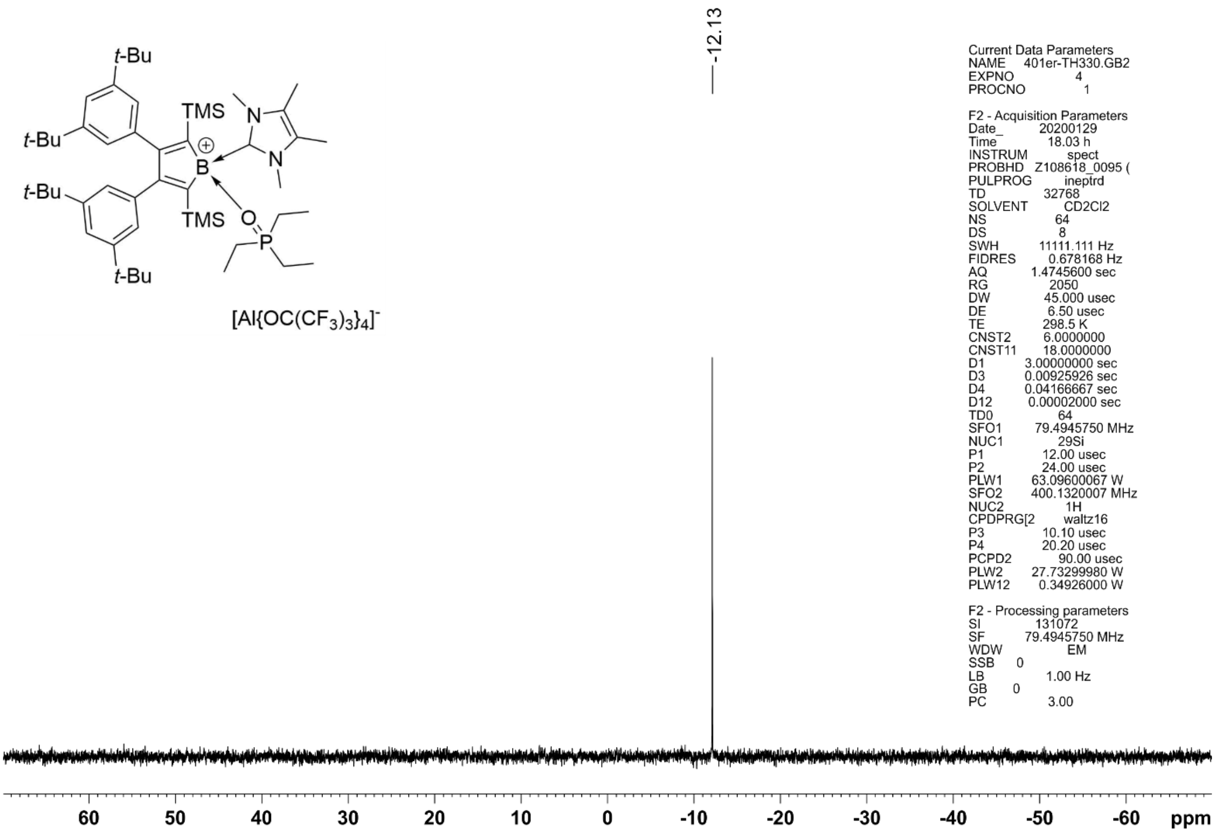

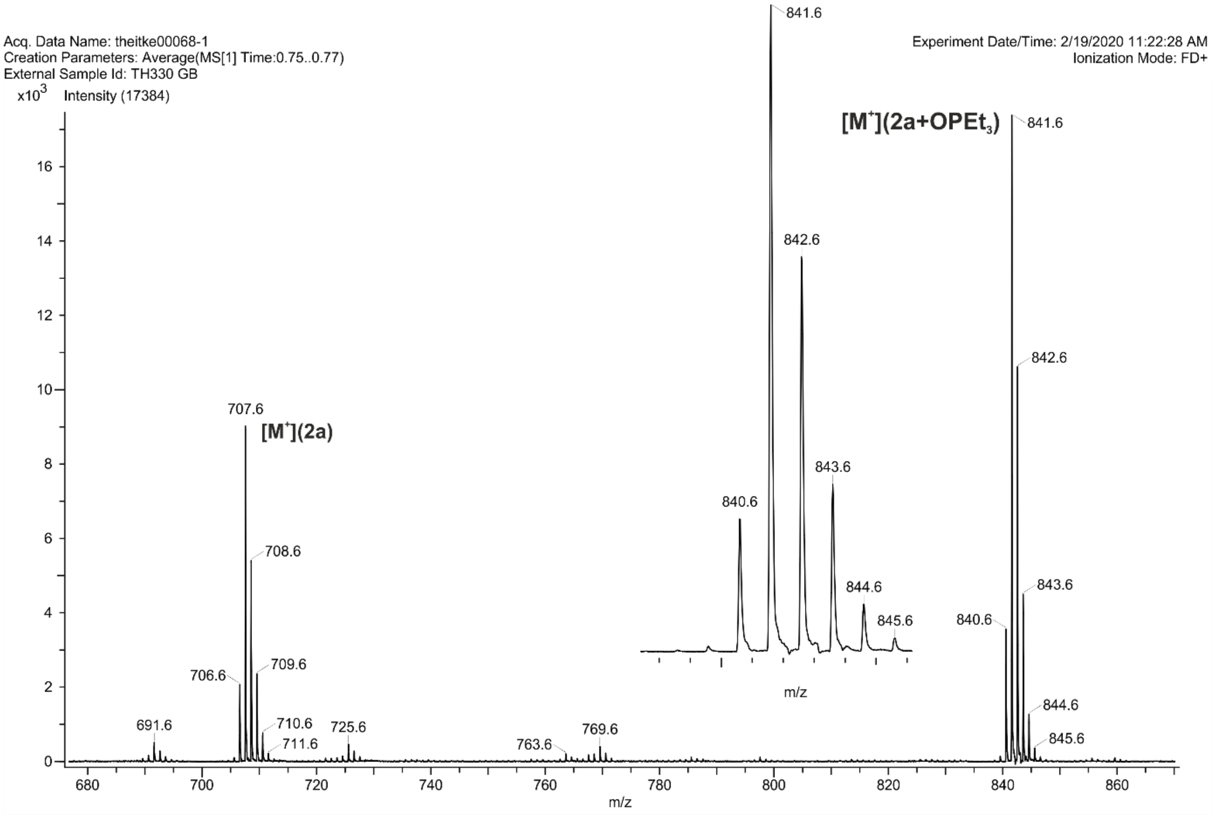

Compound [3][Al{OC(CF$_3$)$_3$}$_4$]

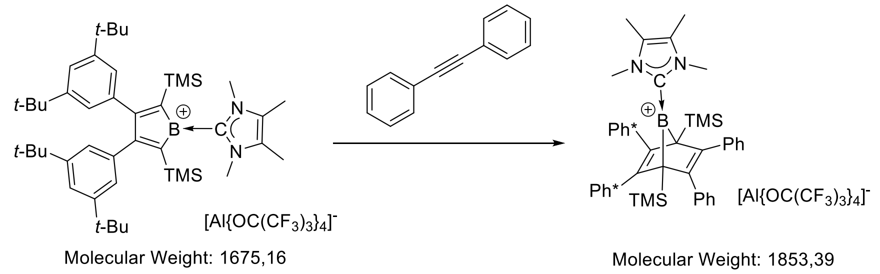

In a glovebox, to a solution of [**2a**][Al{OC(CF$_3$)$_3$}$_4$] (50 mg, 0.030 mmol, 1 eq) in dry, degassed CH_2Cl_2 (1 mL) was added a solution of diphenylacetylene (5.3 mg, 0.030 mmol, 1 eq) in CH_2Cl_2 (0.5 mL). The solution was stirred at ambient temperature overnight and was then carefully layered with pentane (6 mL) and stored at –40 °C overnight. The resulting colourless crystals were isolated by decanting off the mother liquor with a syringe and washing the crystals with cold pentane (2 x 0.5 mL). Compound [**3**][Al{OC(CF$_3$)$_3$}$_4$] (45.5 mg, 0.025 mmol, 82 %) was obtained as a colourless solid.

Note: Attempts were made to obtain a crystal structure data set for [**3**][Al{OC(CF$_3$)$_3$}$_4$]. However, despite large clear, plate-shaped crystals are obtained from the procedure described above, these always immediately turn opaque upon removal from the mother liquor. No suitably diffracting single crystal was obtained despite several attempts.

Note: A computational structure of the molecule suggests an interaction between the Lewis-acidic boron atom and the π-electrons from the adjacent double bonds. This is in line with comparable previously described cases as well as NMR spectroscopic data for the computationally predicted NMR spectrum (see below).

Analytical Data for Compound [4][Al{OC(CF$_3$)$_3$}$_4$]

NMR:

^{1}H (400.13 MHz, 298 K, CD_2Cl_2, $CHDCl_2$ at 5.32 ppm): 7.42 (t, $^4J_{HH}$ = 1.73 Hz, 2H, *p-H$_{Ph*}$*), 7.24–7.19 (m, 6H, *m-H$_{Ph}$* + *p-H$_{Ph}$*), 7.15–7.07 (br, 4H, *o-H$_{Ph}$*), 7.00 (d, $^4J_{HH}$ = 1.73 Hz, 4H, *o-H$_{Ph*}$*), 3.93 (s, 3H, NC*H$_3$*), 3.92 (s, 3H, NC*H$_3$*), 2.33 (s, 3H, C=CC*H$_3$*), 2.32 (s, 3H, C=CC*H$_3$*), 1.19 (s, 36H, C(*Me*)$_3$), –0.49 (s, 18H, Si(*Me*)$_3$).

^{13}C{^{1}H} (100.65 MHz, 298 K, CD_2Cl_2, solvent signal at 53.8 ppm): 152.2 (*m-C$_{Ph*}$*), 147.0 (*C$_{Carbene}$*, not observed in the ^{13}C{^{1}H}-spectrum, assigned via HMBC), 144.8 (*ipso-C$_{Ph}$*/*ipso-C$_{Ph*}$*/C=*C*-Ph/C=*C*-Ph*), 138.3 (*ipso-C$_{Ph}$*/*ipso-C$_{Ph*}$*/C=*C*-Ph/C=*C*-Ph*), 133.9 (*ipso-C$_{Ph}$*/*ipso-C$_{Ph*}$*/C=*C*-Ph/C=*C*-Ph*), 130.4 (C=*C$_{NHC}$*), 129.7 (br, *o-C$_{Ph}$*), 129.2 (C=*C$_{NHC}$*), 128.5 (*m-C$_{Ph}$*/*p-C$_{Ph}$*), 127.8 (*m-C$_{Ph}$*/*p-C$_{Ph}$*), 123.8 (*o-C$_{Ph*}$*), 123.7 (*p-C$_{Ph*}$*), 121.7 (q, $^4J_{CF}$ = 292.3 Hz, *C*F$_3$), 62.7 (*C$_{bridgehead}$*), 36.9 (N$_{NHC}$-*C*H$_3$), 35.9 (N$_{NHC}$-*C*H$_3$), 35.3 (*C*(CH$_3$)$_3$), 31.4 (C(*C*H$_3$)$_3$), 9.24 (C$_{NHC}$-*C*H$_3$), 9.21 (C$_{NHC}$-*C*H$_3$), 0.7 (Si(*C*H$_3$)$_3$).Signals for the quaternary carbon atom of [Al{OC(CF$_3$)$_3$}$_4$]$^-$ were not observed. The signals of *ipso-C$_{Ph}$*, *ipso-C$_{Ph*}$*, C=*C*-Ph or C=*C*-Ph* could not be unambiguously assigned by means of 2D-NMR-methods. One signal for either *ipso-C$_{Ph}$*, *ipso-C$_{Ph*}$*, C=*C*-Ph or C=*C*-Ph* was not observed.

^{11}B (128.38 MHz ,298 K, CD_2Cl_2): -11.4.

^{1}H-^{15}N-HMBC (40.56 MHz, 298 K, CD_2Cl_2): –197.4, –208.1.

^{19}F (376.45 MHz ,299 K, CD_2Cl_2): –75.7.

^{29}Si-INEPT (79.49 MHz, 300 K, CD_2Cl_2): –3.0.

Elemental Analysis: $C_{45}H_{72}BClN_2Si_2$ calcd C 48.60, H 4.46, N 1.51; observed C 49.97, H 4.55, N 1.30.

LIFDI-MS: calcd exact mass of the cation (**3**): 885.61 m/z; observed m/z: 885.6 [M]$^+$.

Spectra Plots for Compound [3][Al{OC(CF$_3$)$_3$}$_4$]

1H-NMR-spectrum of the Diels-Alder product of
[1-[Me4NHC]-2,5-[SiMe3]-3,4-(3', 5'-tBu2Ph)-borole][Al(OC(CF3)3)4] with diphenylacetylene in CD2Cl2

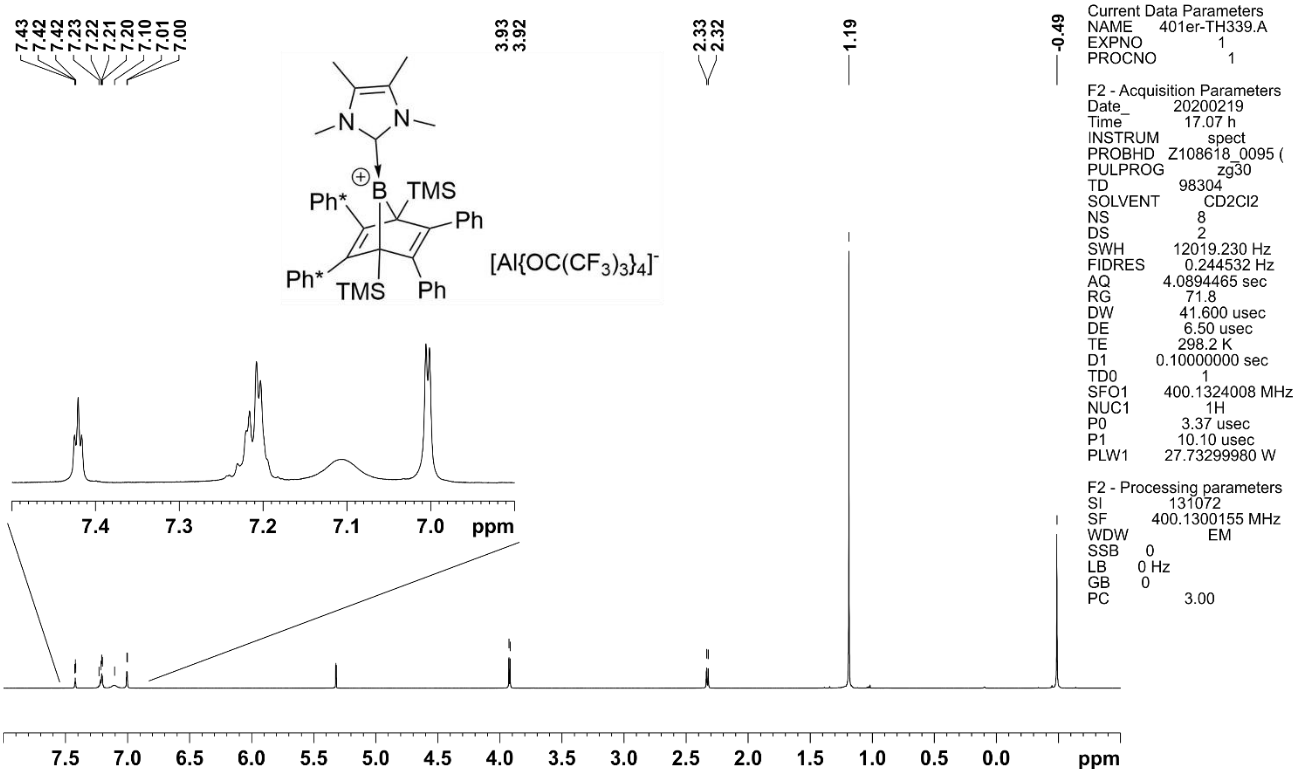

13C{1H}-NMR-spectrum of
[1-[Me4NHC]-2,5-[SiMe3]-3,4-(3', 5'-tBu2Ph)-borole][Al(OC(CF3)3)4] with diphenylacetylene in CD2Cl2

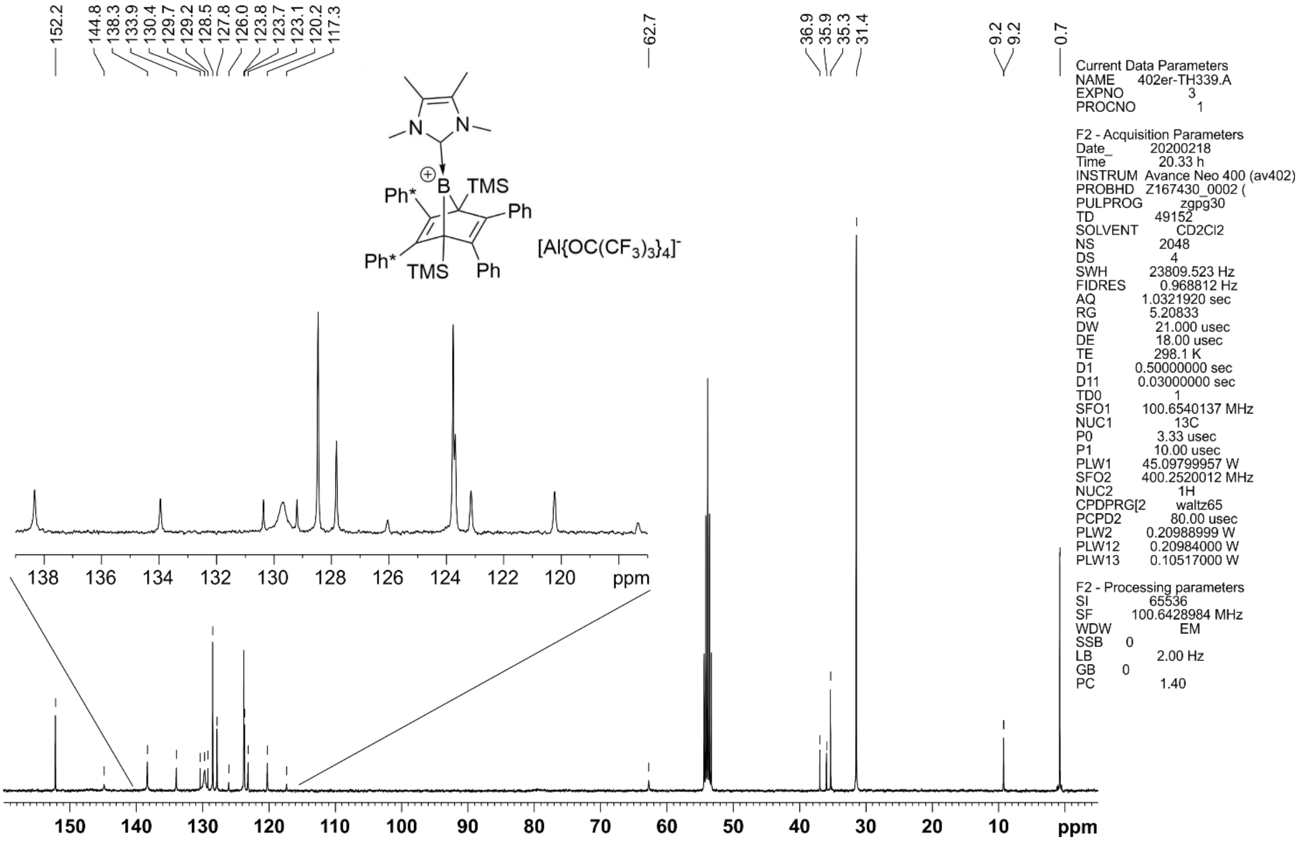

11B-NMR spectrum (background suppressed) of the Diels-Alder product of [1-[Me4NHC]-2,5-[SiMe3]-3,4-(3', 5'-tBu2Ph)-borole][Al(OC(CF3)3)4] with diphenylacetylene in CD2Cl2

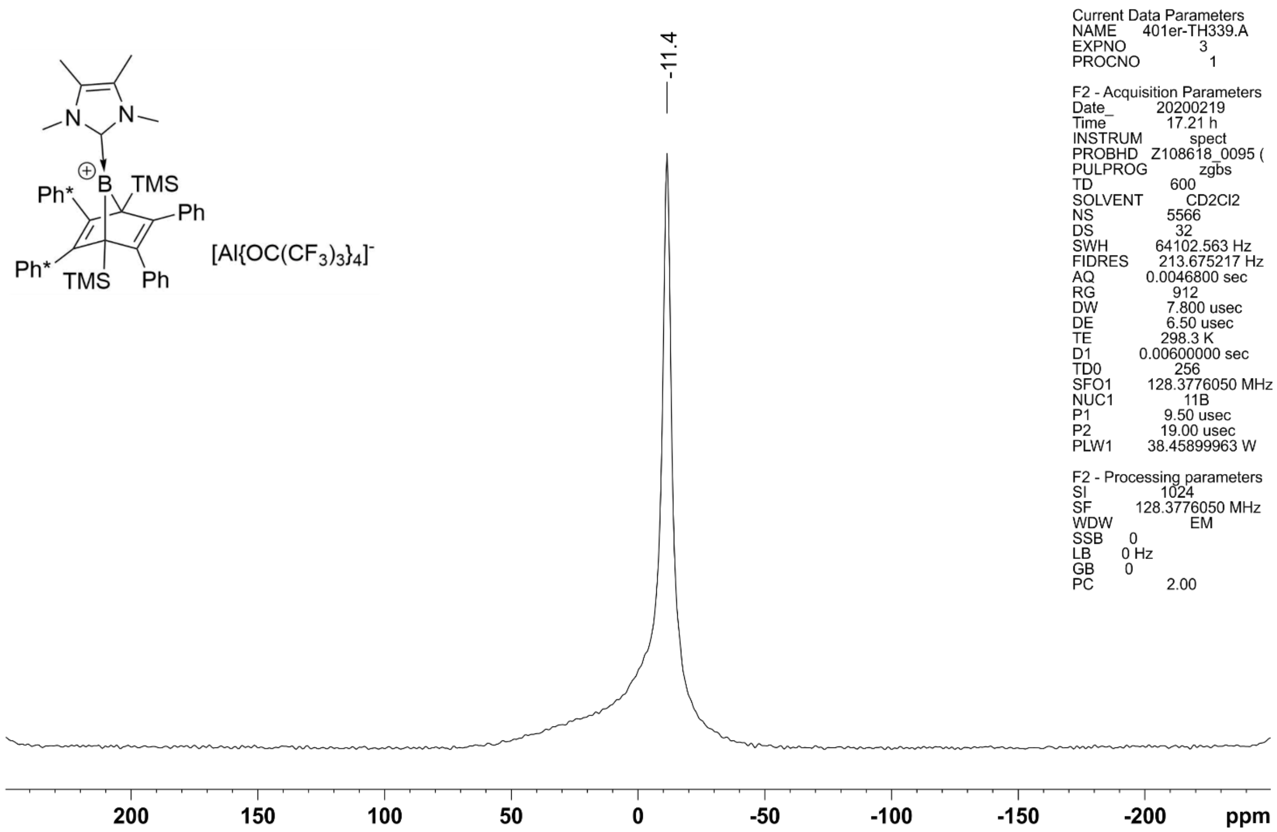

29Si-INEPT-NMR-spectrum of the Diels-Alder product of [1-[Me4NHC]-2,5-[SiMe3]-3,4-(3', 5'-tBu2Ph)-borole][Al(OC(CF3)3)4] with diphenylacetylene in CD2Cl2

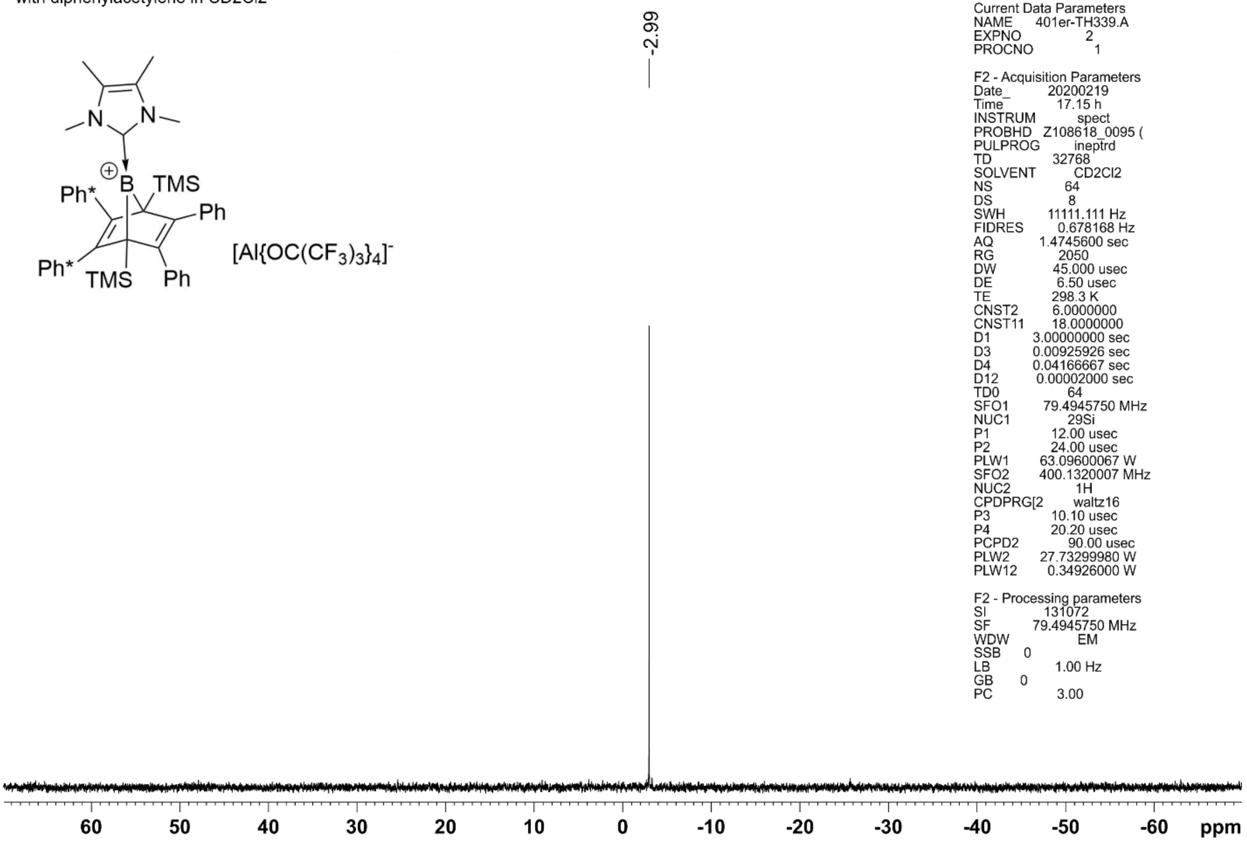

15N-HMBC NMR spectrum of the Diels-Alder product of
[1-[Me4NHC]-2,5-[SiMe3]-3,4-(3', 5'-tBu2Ph)-borole][Al(OC(CF3)3)4] with diphenylacetylene in CD2Cl2

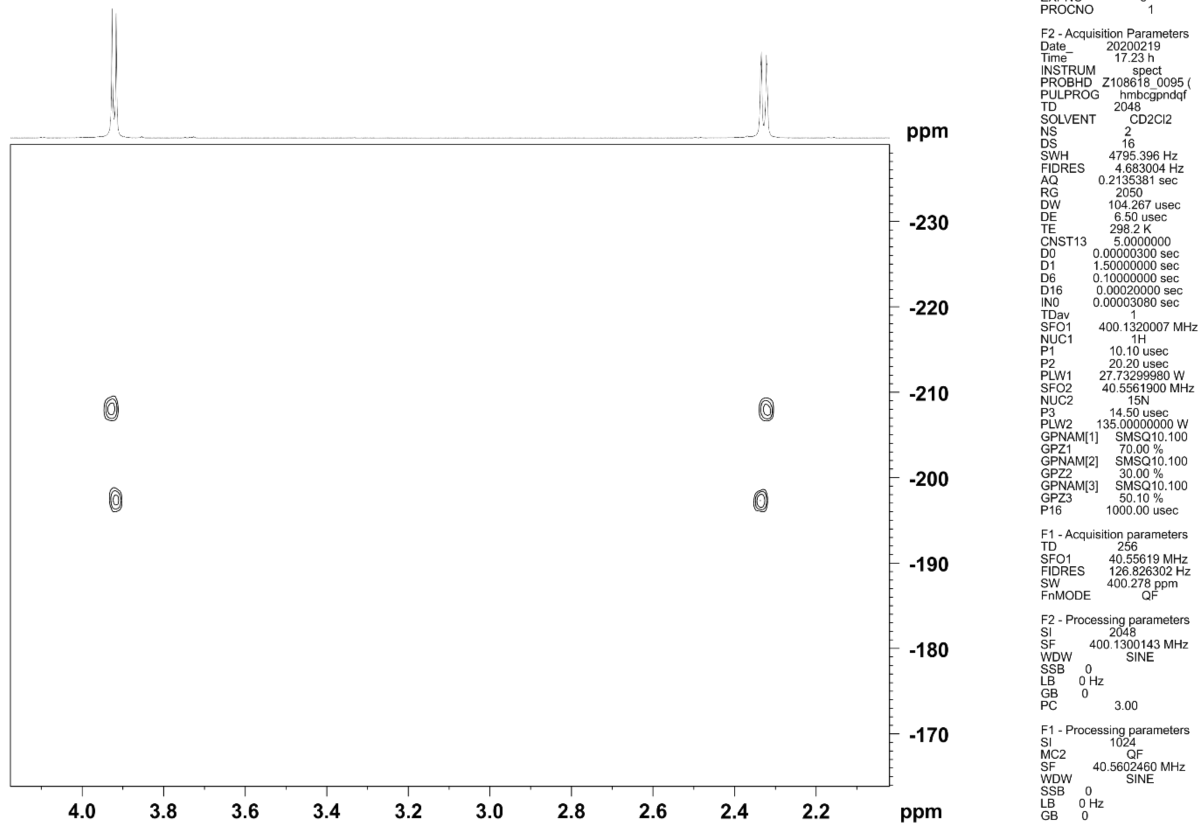

19F{1H}-NMR-spectrum of the Diels-Alder product of [1-[Me4NHC]-2,5-[SiMe3]-3,4-(3', 5'-tBu2Ph)-borole][Al(OC(CF3)3)4]
with diphenylacetylene in CD2Cl2

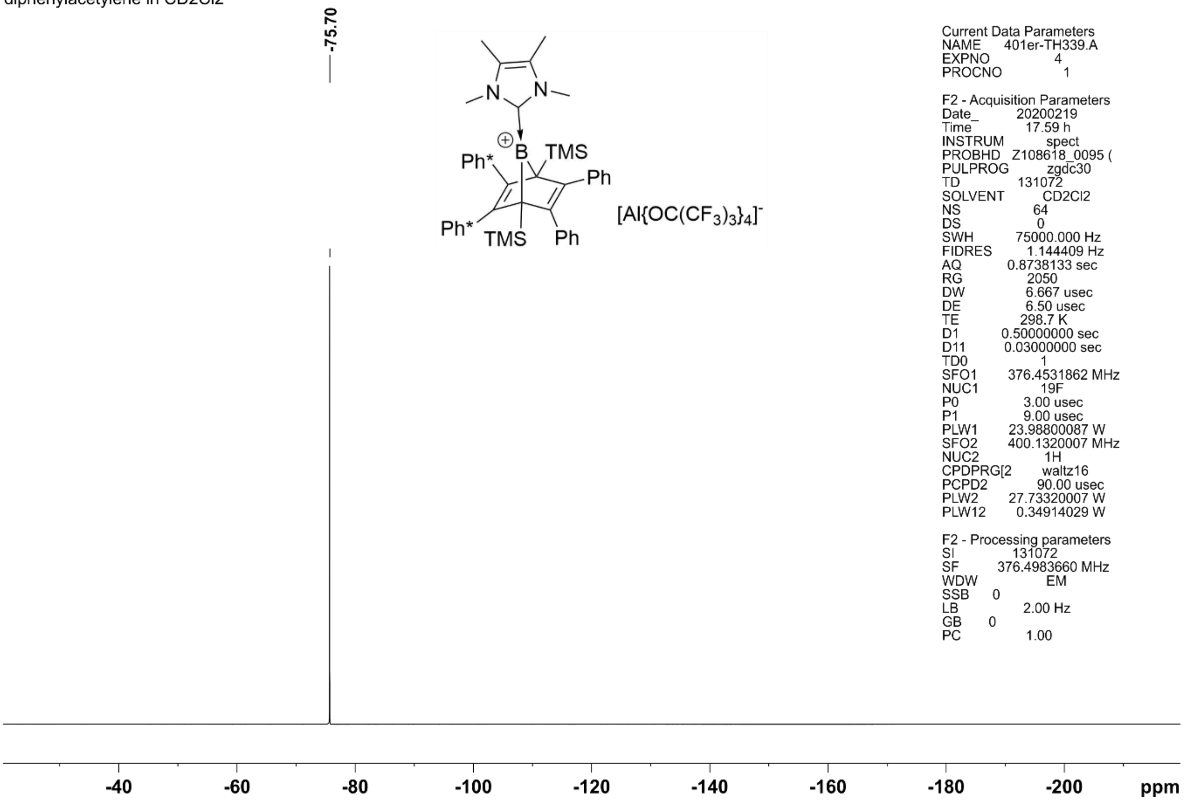

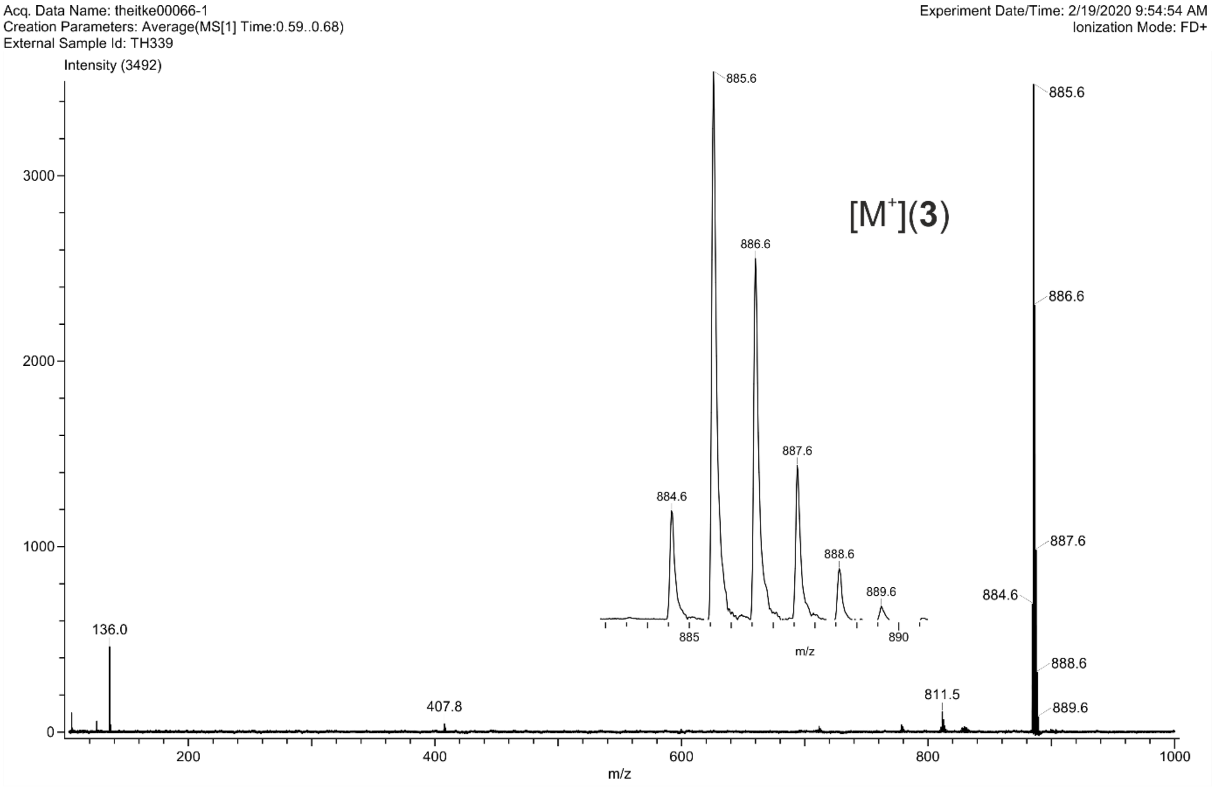
Acq. Data Name: theitke00066-1
Creation Parameters: Average(MS[1] Time:0.59..0.68)
External Sample Id: TH339
Experiment Date/Time: 2/19/2020 9:54:54 AM
Ionization Mode: FD+
Intensity (3492)
[M+](3)
885.6
886.6
887.6
884.6
888.6
889.6
136.0
407.8
811.5
m/z

Compound [4][Al{OC(CF$_3$)$_3$}$_4$]

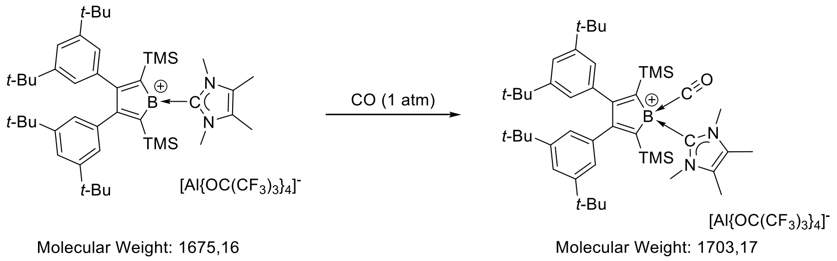

NMR scale reaction: [**2a**][Al{OC(CF$_3$)$_3$}$_4$] (25.3 mg, 0.015 mmol) was dissolved in CD_2Cl_2 in a Young NMR tube. The solution was degassed by one freeze-pump-thaw cycle and afterwards one atmosphere of CO was admitted to the Young tube from a reservoir that was kept at -78°C to freeze out contaminations of moisture. The solution was shaken several times, whereupon the solution rapidly turned from dark yellow to pale yellow. The solution was kept at room temperature and examined spectroscopically.

Preparative scale reaction: In a Young flask, [**2a**][Al{OC(CF$_3$)$_3$}$_4$] (129.2 mg, 0.077 mmol) was dissolved in dry, degassed CH_2Cl_2 (3 mL). The solution was degassed by one freeze-pump-thaw cycle and afterwards one atmosphere of CO was admitted to the Young flask from a reservoir that was kept at -78°C to freeze out contaminations of moisture. The solution was shaken several times, whereupon the solution turned from dark yellow to pale yellow.

Note: (1): When small samples of the solution in dichloromethane are allowed to evaporate to dryness in a glovebox (without reduced pressure), the solutions rapidly turn dark yellow again and NMR spectroscopic examination reveals quantitative reformation of [**2a**][Al{OC(CF$_3$)$_3$}$_4$]. (**2**): When samples of the solution in dichloromethane (0.5 mL) are diluted with excess of pentane (12 mL) and subsequent storage at –40°C, [**4**][Al{OC(CF$_3$)$_3$}$_4$] precipitates as a pale yellow solid. Samples of this solid stored open in a glovebox atmosphere turned dark yellow and IR examination after a few hours did not reveal the CO stretching band anymore. (**3**): Crystals suitable for X-ray analysis were grown by layering a sample (0.5 mL) of the reaction mixture with pentane (1.5 mL) and storage at –40°C. The crystals were harvested, picked and mounted at low temperature but loose crystallinity after a few minutes. A mediocrely diffracting crystal was examined. (**4**): The NMR-sample kept under CO atmosphere over a period of days slowly reveals increasing signals of decomposition.

Analytical Data for Compound [4][Al{OC(CF$_3$)$_3$}$_4$]

NMR:

^{1}H (400.13 MHz, 298 K, CD_2Cl_2, $CHDCl_2$ at 5.32 ppm): 7.26 (t, $^{4}J_{HH}$ = 1.79 Hz, 2H, *p-H$_{Ar}$*), 6.66 (d, $^{4}J_{HH}$ = 1.79 Hz, 4H, *o-H$_{Ar}$*), 3.98 (br, 3H, N*CH$_3$*), 3.31 (br, 3H, N*CH$_3$*), 2.34 (br, 3H, C=C*CH$_3$*), 2.26 (br, 3H, C=C*CH$_3$*), 1.15 (s, 36H, C(*Me*)$_3$), –0.19 (s, 18H, Si(*Me*)$_3$).

^{13}C{^{1}H} (100.65 MHz, 298 K, CD_2Cl_2, solvent signal at 53.8 ppm): 180.8 (borole-*C$_{3,4}$*), 169.5 (br, *C*O), 150.6 (*m-C$_{Ar}$*), 150.1 (borole-*C$_{2,5}$*), 148.5 (br, *C$_{carbene}$*, not observed in the ^{13}C{^{1}H}-spectrum, assigned via HMBC), 138.8 (*ipso-C$_{Ar}$*), 129.2 (C=*C$_{NHC}$*), 128.7 (C=*C$_{NHC}$*), 122.9 (*o-H$_{Ar}$*), 122.1 (*p-H$_{Ar}$*), 120.3 (q, $^{4}J_{CF}$ = 292.2 Hz, *C*F$_3$), 35.0 (*C*(CH$_3$)$_3$), 34.5 (br, N$_{NHC}$-*C*H$_3$), 31.5 (N$_{NHC}$-*C*H$_3$, superimposed by C(*C*H$_3$)$_3$-signal, assigned via HSQC), 31.5 (C(*C*H$_3$)$_3$), 9.5 (C$_{NHC}$-*C*H$_3$), 8.9 (C$_{NHC}$-*C*H$_3$), 0.3 (Si(*C*H$_3$)$_3$). Signals for the quaternary carbon atom of [Al{OC(CF$_3$)$_3$}$_4$]$^-$ were not observed.

^{11}B (128.38 MHz ,298 K, CD_2Cl_2): –18.8.

^{19}F (376.45 MHz ,299 K, CD_2Cl_2): –75.7.

^{29}Si-INEPT (79.49 MHz, 298 K, CD_2Cl_2): –8.2.

Elemental Analysis: $C_{62}H_{72}BN_2O_5F_{36}AlSi_2$ calcd C 43.72, H 4.26, N 1.64; observed C 44.63, H 4.52, N 1.55.

Crystal structure of Compound [4][Al{OC(CF$_3$)$_3$}$_4$]

[4][Al{OC(CF$_3$)$_3$}$_4$] crystallised from pentane layered dichloromethane solutions in a freezer (-40°C). Crystals are very sensitive and diffracted very weakly at resolutions higher than 0.9Å. Structure solutions and refinement were only possible in space group P1 with two independent molecules of **[4][Al{OC(CF$_3$)$_3$}$_4$]** in the asymmetric unit. Attempts to identify an inversion center using PLATON failed. An inversion center for the conversion of the cation fragments could be found, however this inversion failed to match the anions (heavy atom positions). The counteranions and lattice pentane and dichloromethane molecules were found to be disordered. The structure clearly confirms the identity of the CO-complex. (Details see below).

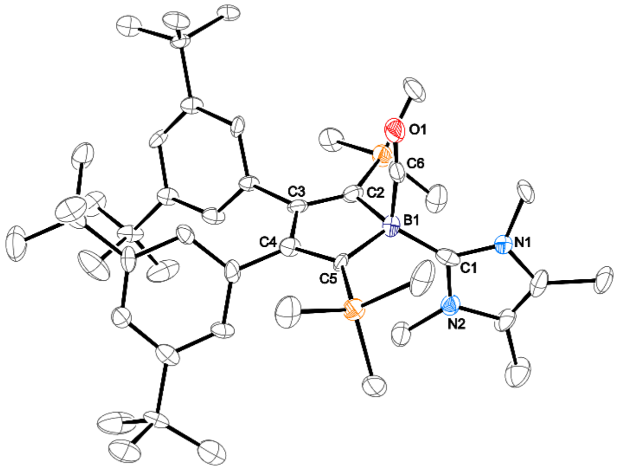

ORTEP plot of the molecular structure of one molecule of cation [**4**]$^+$. Atomic displacement parameters are drawn at 50% probability level. Hydrogen atoms, counteranions, lattice solvents and a second independent cation have been omitted for the sake of clarity. Selected bond lengths [Å] and angles [°] are given and the corresponding numbers for the second independent molecule are given in brackets[]: B1-C1 1.58(2) [1.60(2)], B1-C6 1.56(2) [1.58(2)], B1-C2 1.67(2) [1.62(2)], B1-C5 1.63(2) [1.62(2)], C2-C3 1.38(2) [1.34(2)], C4-C5 1.38(2) [1.37(2)], C3-C4 1.49(2) [1.48(2)], C6-O1 1.10(2) [1.14(2)]; O1-C6-B1 171.5[169.7].

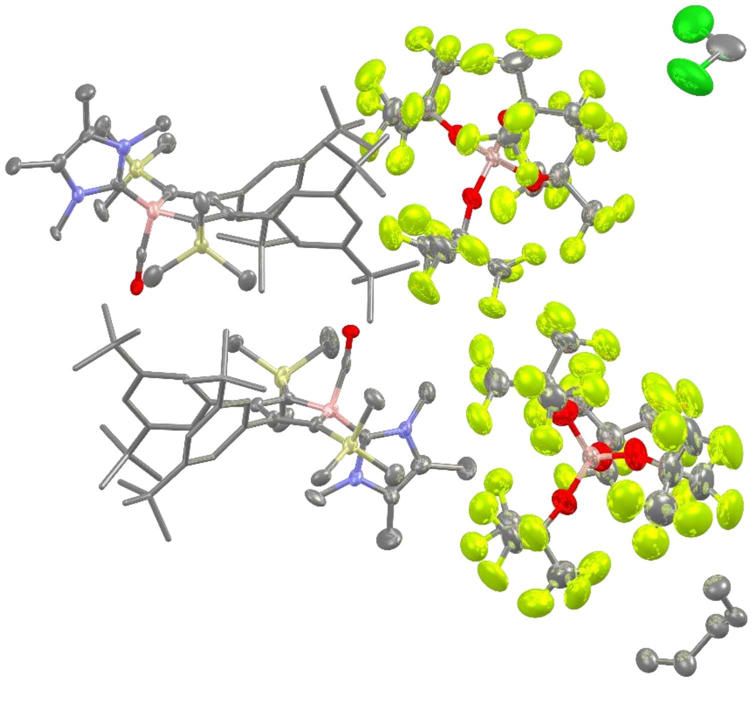

MERCURY POVRAY plot of the whole asymmetric unit of **[4][Al{OC(CF$_3$)$_3$}$_4$]** without disorder.

Spectra Plots for Compound [4][Al{OC(CF_3)$_3$}$_4$]

1H-NMR-spectrum of [1-[Me4NHC]-2,5-[SiMe3]-3,4-(3', 5'-tBu2Ph)-borole]*CO
with [Al(OC(CF3)3)4] counteranion in CD2Cl2 under CO atmosphere

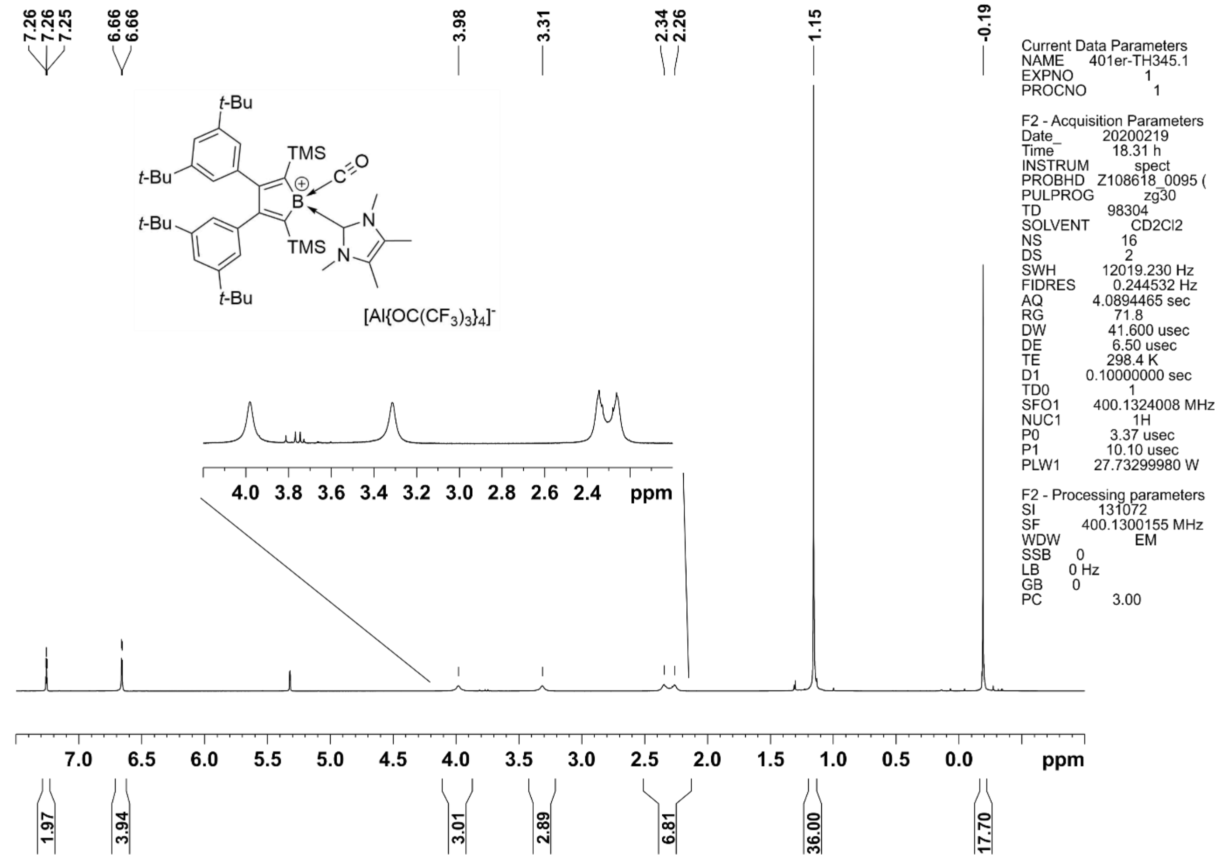

13C{1H}-NMR-spectrum of [1-[Me4NHC]-2,5-[SiMe3]-3,4-(3', 5'-tBu2Ph)-borole]*CO
with [Al(OC(CF3)3)4] counteranion in CD2Cl2 under CO atmosphere

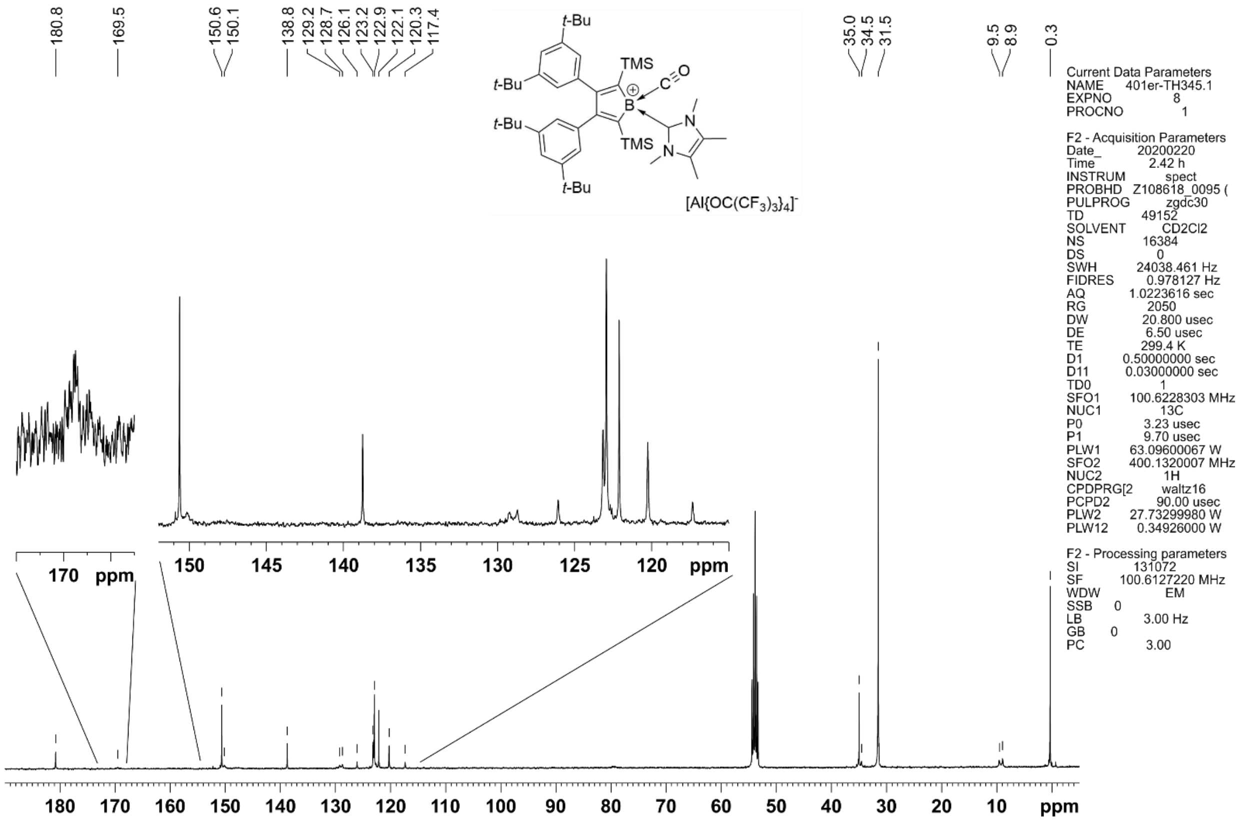

11B-NMR spectrum (background suppressed) of [1-[Me4NHC]-2,5-[SiMe3]-3,4-(3', 5'-tBu2Ph)-borole]*CO with [Al(OC(CF3)3)4] counteranion in CD2Cl2 under CO atmosphere

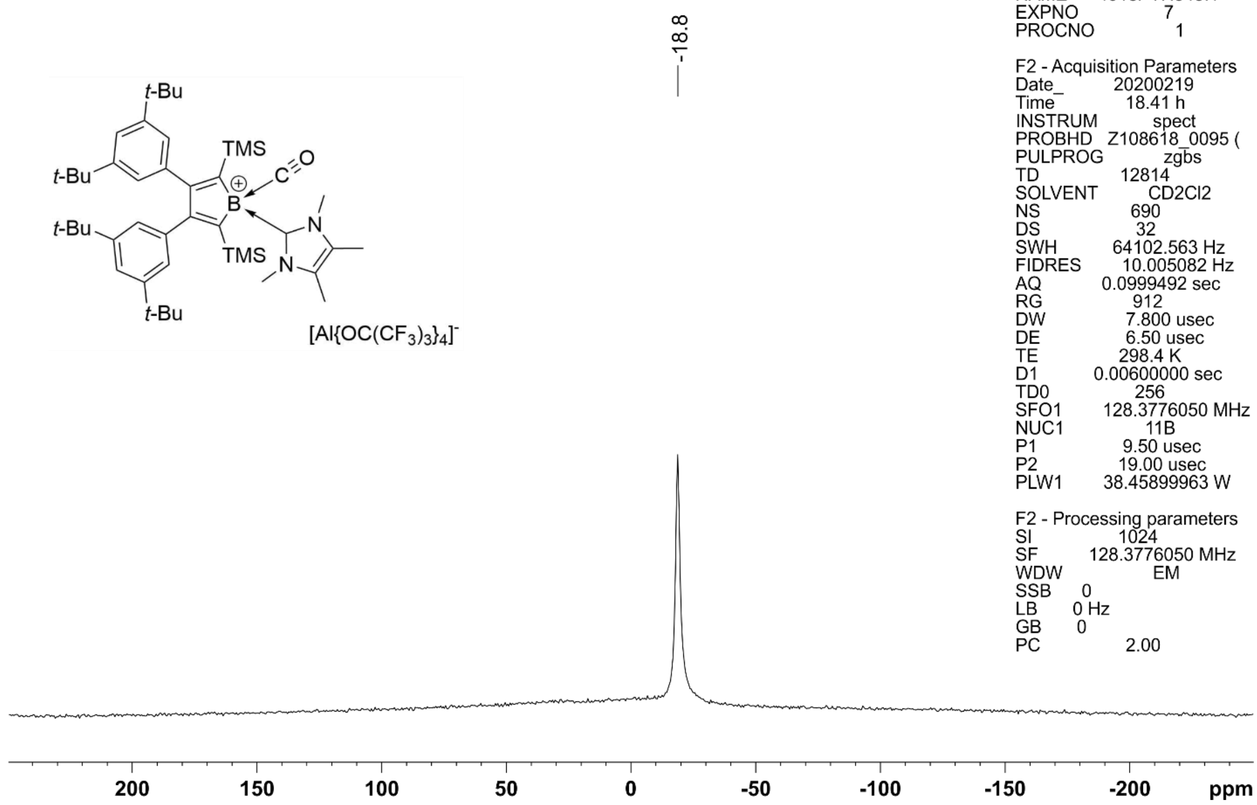

19F{1H}-NMR-spectrum of [1-[Me4NHC]-2,5-[SiMe3]-3,4-(3', 5'-tBu2Ph)-borole]*CO with [Al(OC(CF3)3)4] counteranion in CD2Cl2 under CO atmosphere

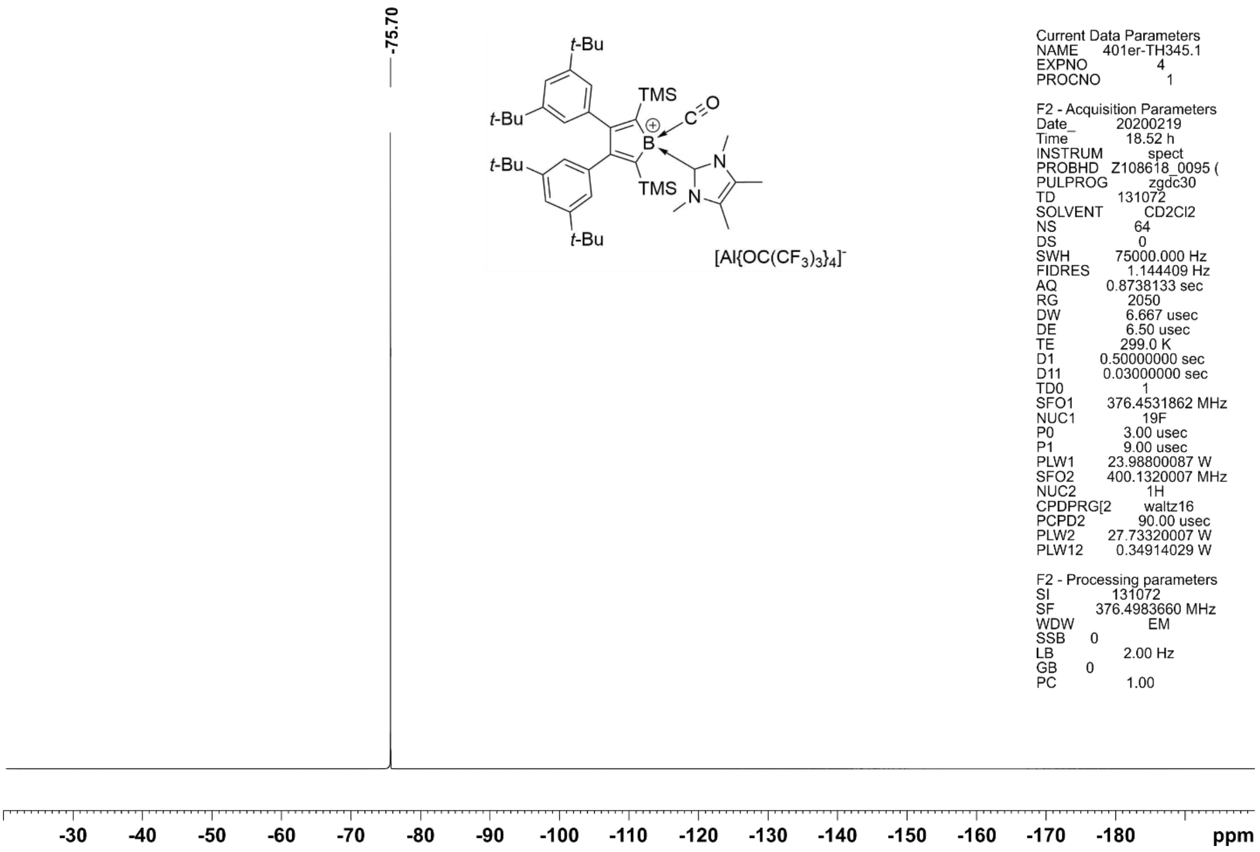

29Si-INEPT-NMR-spectrum of [1-[Me4NHC]-2,5-[SiMe3]-3,4-(3', 5'-tBu2Ph)-borole]*CO with [Al(OC(CF3)3)4] counteranion in CD2Cl2 under CO atmosphere

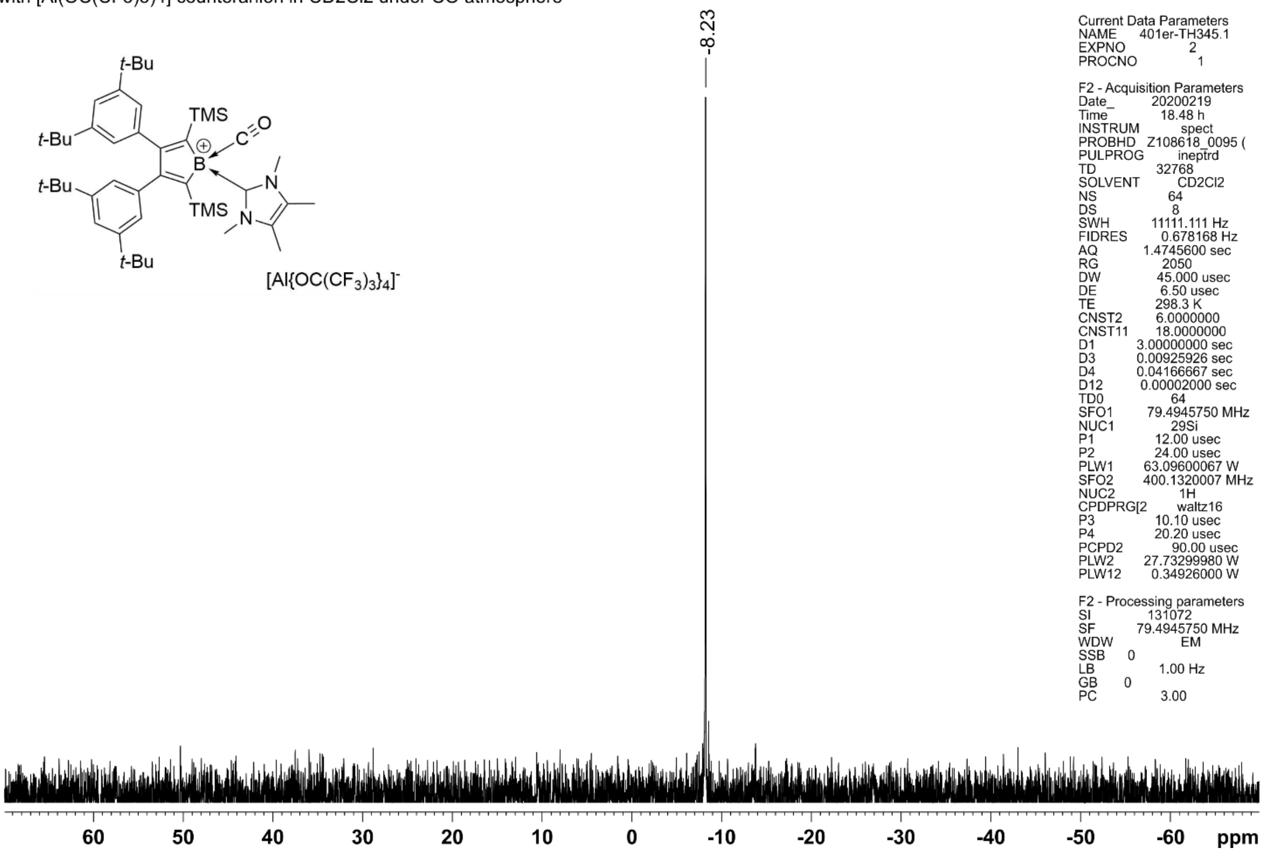

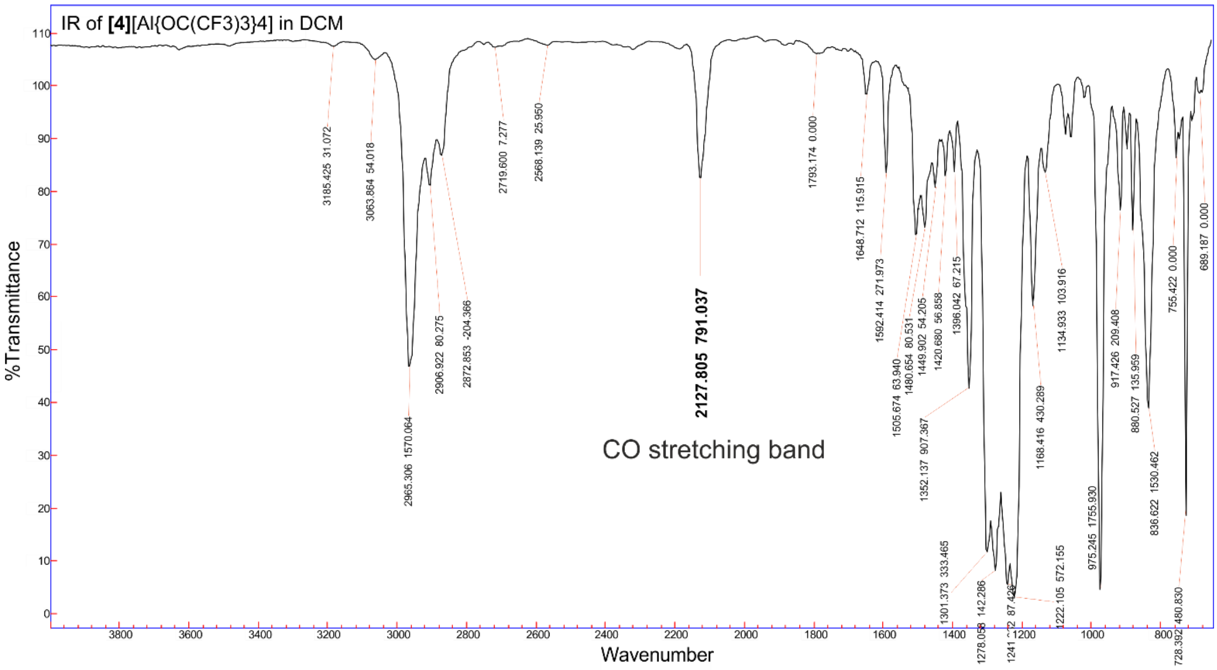

Crystallographic Details

General Data Acquisition and Processing

X-ray data for **1a** and **[2a][Al{OC(CF_3)$_3$}$_4$]** were collected on Bruker APEX II CCD diffractometers with either Mo Kα radiation. The data were integrated using SAINT implemented in Bruker's APEX3 programme suite.[7] SADABS was used for multi-scan absorption correction.[8] Structure solution was performed with SHELXT[9] and refined using SHELXL[10] along the graphical user interphase of ShelXle.[11] In some cases DSR has been applied to treat disordered solvent molecules.[12] All hydrogen atoms were placed with a riding model. Further details on the individual data sets are tabulated in the analytical section of each compound.

Crystallographic and Refinement Details 1

The crystals lost crystallinity upon cooling to 100K and 120K. Therefor data acquisition was performed at 140K. Two *t*-Bu groups and a lattice pentane molecule were found to be (severely) disordered over two positions and modelled using SAME, SIMU and RIGU commands.

Crystallographic and Refinement Details [2a][Al{OC(CF_3)$_3$}$_4$]

Crystals were extreme sensitive to air and moisture and were picked under a continuous stream of cold nitrogen (-100°C) using an XTEMP2 set-up. Even under these conditions the crystals decomposed after a few minutes. A small crystal was chosen for data collection which only diffracted weakly at higher resolutions.

One *t*Bu group of the cation and one OC(CF_3)$_3$ group of the aluminate anion were found disordered over two positions and and modelled accordingly using SAME, SIMU and RIGU commands. A residual electron density hints at further minor disorder in the counter anion, however attempts to model this disorder did not lead to improvement.

Crystallographic and Refinement Details [4][Al{OC(CF_3)$_3$}$_4$]

Crystals were extreme sensitive to air and moisture and were picked under a continuous stream of cold nitrogen (-100°C) using an XTEMP2 set-up. Even under these conditions the crystals lost crystallinity after a few minutes. A crystal was chosen for data collection which only diffracted very weakly at resolutions higher than 0.95 Å. Meaningful structure solution and refinement were only possible in space group P1, our attempts to identify an inversion center using PLATON failed. An inversion center for the cation fragments can be identified however this inversion failed to match the anions (central heavy atom positions). Due to limited amount of high angle data, only limited refinement of disorder was possible. Lattice dichloromethane molecule was refined with a fixed occupancy of 65% and residual electron density around the lattice pentane molecule indicates further possible disorder which was not modelled. Reflections 001 and 010 were likely affected by the beam stop and not observed. They were omitted upon refinement. Due to weak reflections at higher angles only a limited C-C bond precision could be obtained. A small fragment of a second crystal appears to be stuck to the crystal under investigation which likely corresponds to the starting material. Reflection caused by this very minor component may affect a small number of reflections.

Tabulated Crystallographic Details 1

	**1a**×(C_5H_{12})	**[2a][Al{OC(CF$_3$)$_3$}$_4$]** ×(CH_2Cl_2)	**[4][Al{OC(CF$_3$)$_3$}$_4$]** ×0.65(CH_2Cl_2) ×(C_5H_{12})
CCDC number	1982736	1982737	1986857
empirical formula	$C_{50}H_{84}Si_2N_2BCl$	$C_{62}H_{74}BN_2Si_2AlO_4F_{36}Cl_2$	$C_{129.65}H_{155.30}Al_2B_2Cl_{1.30}F_{72}N_4O_{10}Si_4$
formula weight	815.63	1760.10	3531.69
T / K	140(2)	100(2)	100(2)
λ / Å	0.71073, Mo K_α	0.71073, Mo K_α	0.71073, Mo K_α
crystal system	monoclinic	triclinic	triclinic
space group	*C2/c*	*P-1*	*P1*
a / Å	32.237(3)	11.4143(9)	11.3628(6)
b / Å	21.0955(16)	18.1532(14)	18.0477(10)
c / Å	19.1912(15)	20.9198(15)	20.6703(11)
α	90	110.574(4)	103.870(3)
β / °	125.1870(10)	96.616(5)	93.779(3)
γ	90	102.125(4)	101.201(3)
V / Å³	10666.4(14)	3882.2(5)	4008.7(4)
Z	8	2	1
ρ / Mg m^{-3}	1.016	1.506	1.463
μ / mm^{-1}	0.148	0.257	0.207
F(000)	3584	1792	1803
crystal size / mm^3	0.58/0.14/0.13	0.28/0.13/0.05	0.28/0.13/0.11
θ range / °	1.2 to 25.4	1.2 to 25.2	1.8 to 24.5
index ranges	$-38\leq h \leq 38$	$-13\leq h \leq 13$	$-13\leq h \leq 13$
	$-25\leq k \leq 25$	$-21\leq k \leq 21$	$-21\leq k \leq 21$
	$-23\leq l \leq 23$	$-24\leq l \leq 24$	$-24\leq l \leq 24$
refl. Collected	85636	60890	82071
indep. reflections/ R_{int}	9442 / 0.073	13816 / 0.078	26581 / 0.054
completeness to θ_{max}	96.3 %	98.3%	99.5%
data/restraints/ parameters	9442 /516/637	13816 / 1100 / 1143	26579/5982/2310
GooF	1.08	1.03	1.24
final R indices [I>2sigma(I)] R_1 / wR_2	0.056 / 0.142	0.075 / 0.177	0.112 / 0.292
R indices (all data) R_1 / wR_2	0.072 / 0.150	0.128 / 0.201	0.150 / 0.321
largest diff. peak and hole / eÅ^{-3}	0.50/ -0.28	1.21 / -0.87	1.11 / -0.78
absorption correction	multi-scan	multi-scan	multi-scan
Flack			0.42(5)

Cyclovoltammetry

General Data Acquisition and Processing

Cyclovoltammetry measurements were performed in a glovebox using a AUTOLAB PGSTAT12 potentiostat applying a glassy carbon working electrode, a Pt wire counter electrode and a silver wire quasi-reference electrode. Measurements were performed using 0.4M solutions of $[NBu_4][Al\{OC(CF_3)_3\}_4]$[5] in both CH_2Cl_2 and 1,2-difluorobenzene. All potentials are given versus internal Fc/Fc^+.

	1,2-difluorobenzene	CH_2Cl_2
**$E_{1/2}$ [2a]$^+$**	-1.03 V	-1.06V
**$E_{1/2}$ A-Mes**	-1.89 V	-1.95 V

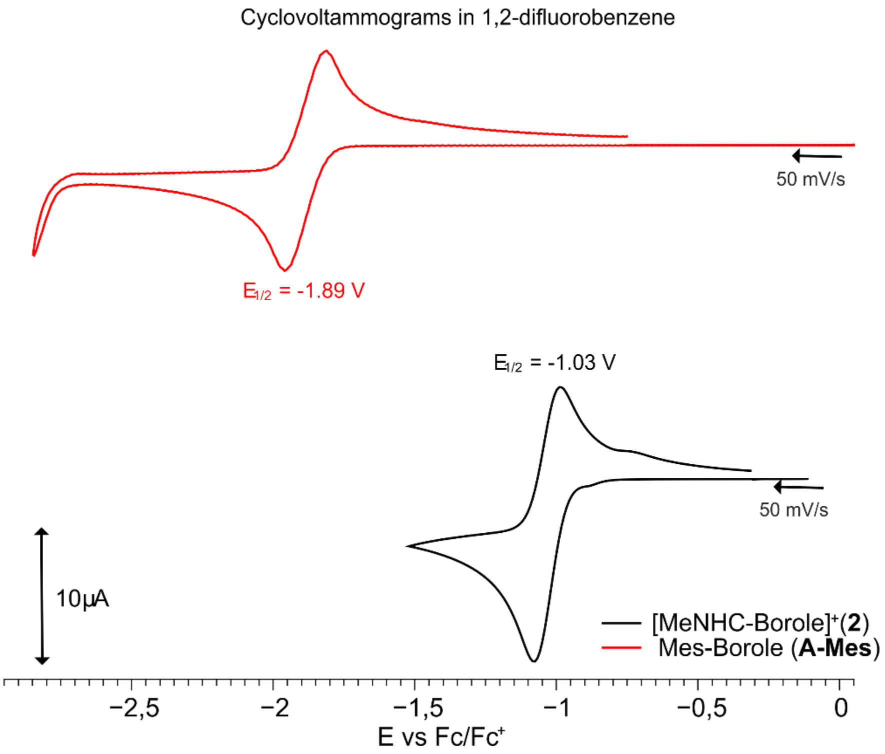

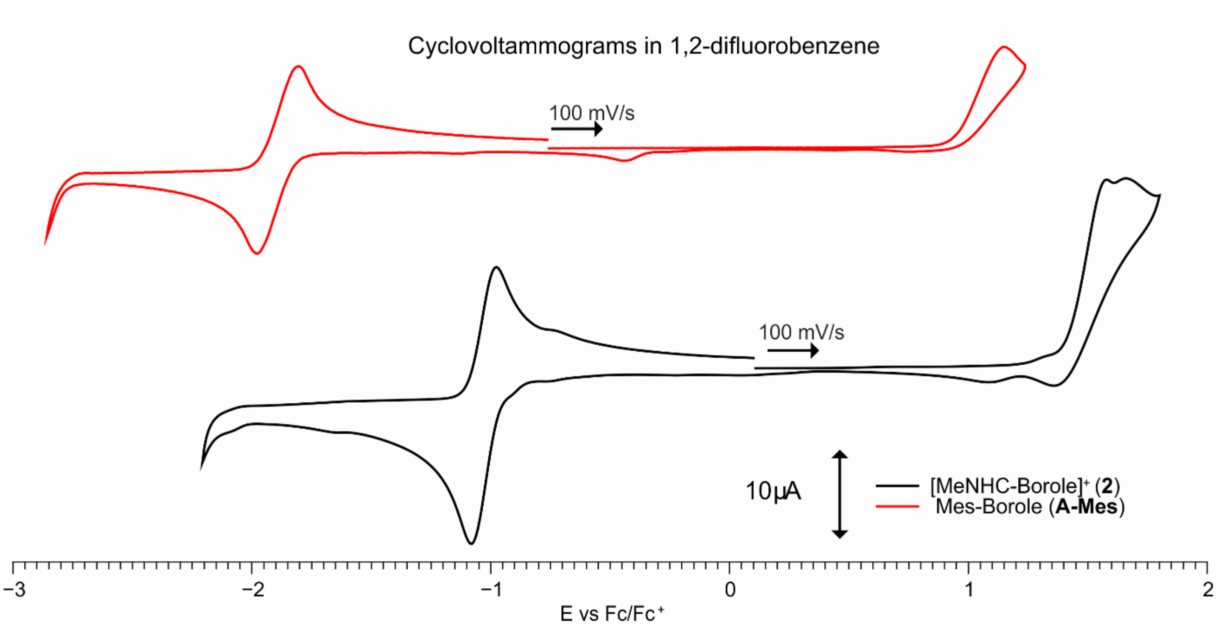

Cyclovoltammograms in 1,2-difluorobenzene
100 mV/s
100 mV/s
10μA
[MeNHC-Borole]⁺ (2)
Mes-Borole (A-Mes)
−3
−2
−1
0
1
2
E vs Fc/Fc⁺

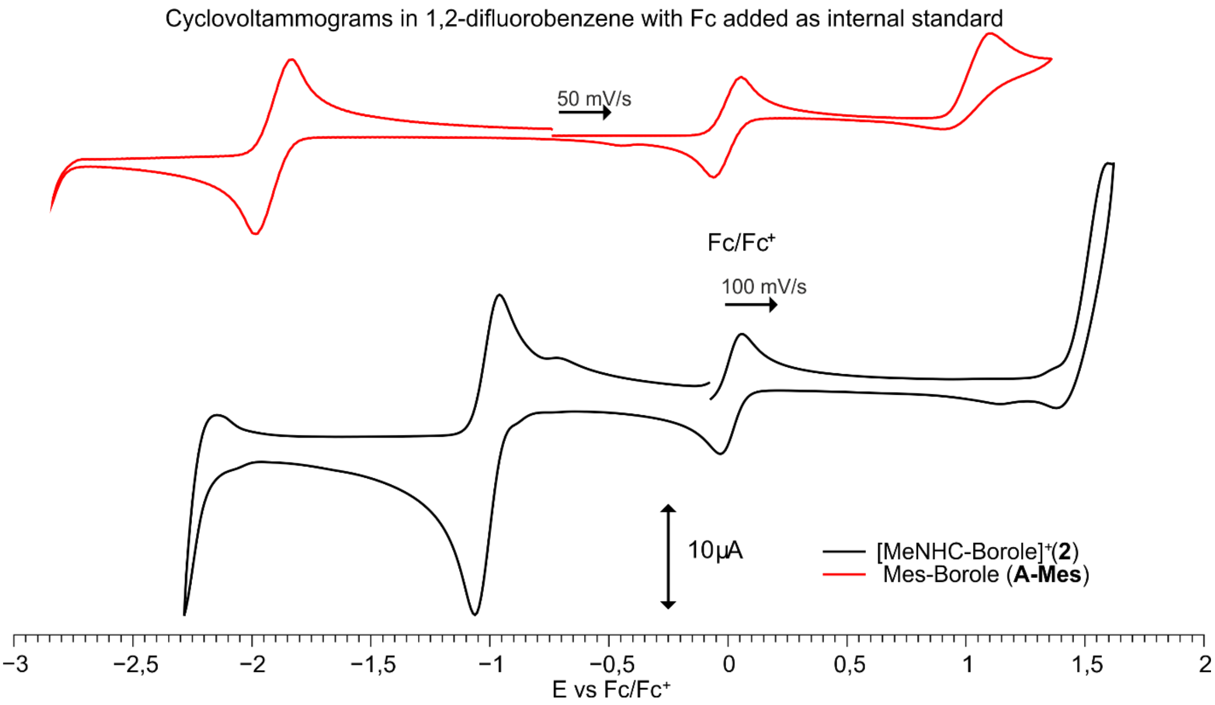

Cyclovoltammograms in 1,2-difluorobenzene with Fc added as internal standard
50 mV/s
Fc/Fc⁺
100 mV/s
10μA
[MeNHC-Borole]⁺(2)
Mes-Borole (A-Mes)
−3
−2,5
−2
−1,5
−1
−0,5
0
0,5
1
1,5
2
E vs Fc/Fc⁺

Computational Details

Structure Optimisation, Frequency Calculation and Thermochemical Approximations

Computational examination was performed using ORCA (version 4.1.).[13] All neutral structures were optimised starting from experimental X-Ray structures on RI-BP86-D3BJ[15] def2SVP/J model chemistry[16] in the gas phase followed by a frequency calculation on the same level of theory and when necessary, thermochemical corrections were taken from these frequency calculations. The computational structure of **A-Mes** was previously reported.[2b] For all compounds no imaginary frequencies were observed confirming true minima. All structures were then reoptimized using BP86-D3BJ-def2TZVP/J model chemistry and all energies reported as well as NBO analyses[14] refer to these gasphase structures. For the spectroscopic properties of cationic compounds, the structure was optimised applying CPCM solvation model for dichloromethane and TD-DFT spectroscopic properties were subsequently calculated based on these structures. Graphical depictions were created using ChemCraft.[17] NBO and NRT analyses were conducted using NBO7.0.[14] For NRT a model complex $(HC)_4B(^{Me}NHC)$ was optimised in RI-BP86/def2-TZVP with the dihedral between the two planes restricted to the experimentally observed value in [**2a**]$^+$. Resonance structures were searched with NRTE2 threshold at 3 kcal mol^{-1} in the local framework of the ring atoms.

Computational Structure of [3]$^+$

Gasphase structure of [4]$^+$ (using BP86-D3BJ-def2TZVP). Hydrogen atoms omitted for clarity. Distances given in Å.

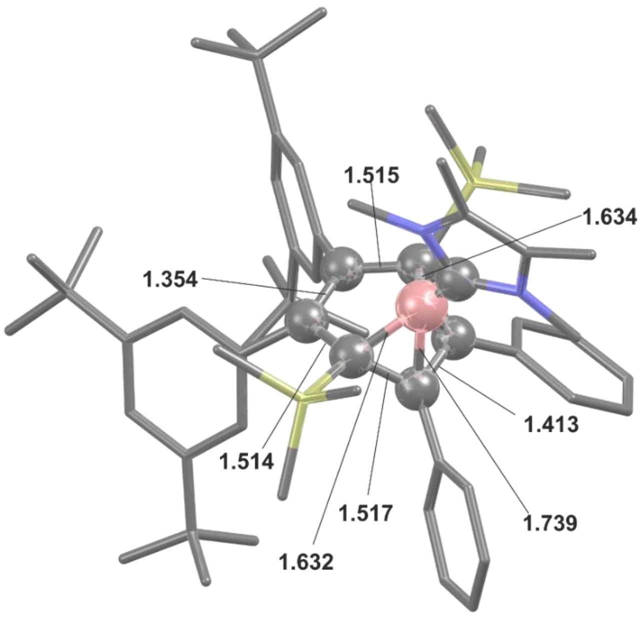

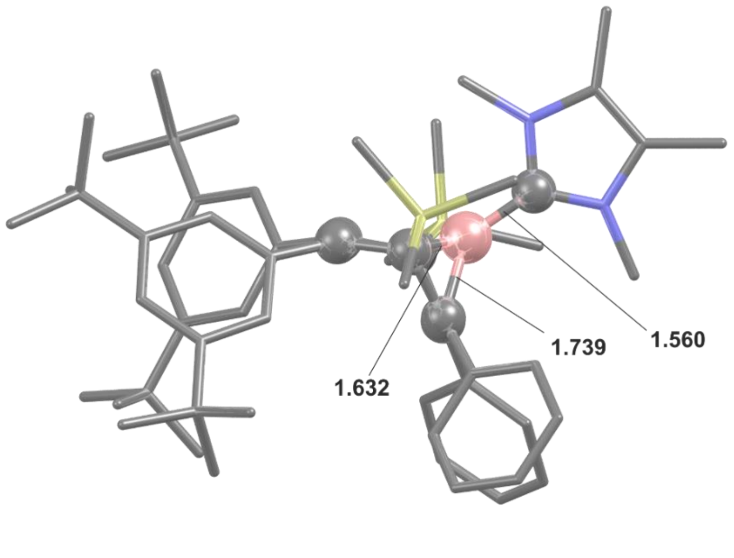

Computational Structure of [4]⁺

Gasphase structure of $[4]^+$ (using BP86-D3BJ-def2TZVP). Hydrogen atoms omitted for clarity. Distances given in Å.

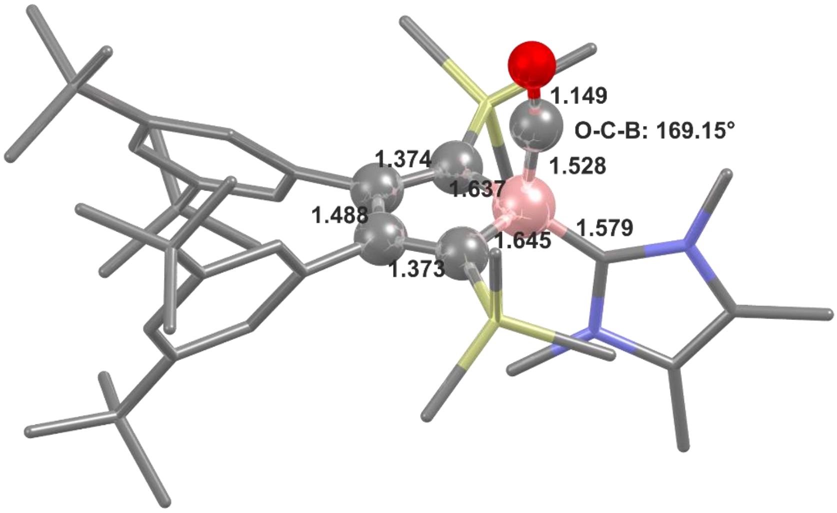

ETS-NOCV

ETS-NOCV analysis[18] was performed using ADF2019.102 programme suite with BP86-D3BJ[15-16]/TZ2P[19] model chemistry on the basis of the gas-phase optimised structure obtained from ORCA.[20] Fragments chosen were neutral CO and cationic [**2a**]$^+$. Depictions of deformation densities and SFO were prepared using ADF2019.102.

Computational assessment of NMR spectroscopic features

GIAO-NMR spectroscopic properties including NICS[21] values were calculated as implemented as the default in ORCA4.1 applying RIJK-PBE0[22] functional on gasphase structures previously optimised using the RI-BP86-D3BJ-def2TZVP/J model chemistry.[16] NICS scans were performed determining the centroid of the C_4B plane (in ChemCraft) (NICS0) and on a series of points along an orthogonal vector to C_4B starting from there.

Computational reproduction of NMR spectroscopic properties for cations [**2a**]$^+$, [**3**]$^+$ and [**4**]$^+$ was performed using GIAO method and RIJK-PBE0[22] functional and def2-TZVP basis set (C,H) and def2-TZVPP basis set (all hetero-atoms) applying a polarized continuum solvation model for dichloromethane (CPCM = CH2Cl2) on structures previously optimised using the RI-BP86-D3BJ-def2TZVP/J-(CPCM=CH2Cl2) model chemistry. For neutral molecules, gasphase structures were taken and no solvation model was applied. Calculated chemical shifts were obtained according to:

$$\delta_{calc} = \sigma_{ref} - \sigma_{calc}$$

with $\sigma_{ref}(^{13}C)$ = 186.9 ppm, $\sigma_{ref}(^{29}Si)$ = 332.6 ppm and $\sigma_{ref}(^{11}B)$ = 101.2 ppm.

For ^{13}C-NMR shifts, from comparison of experimental and calculated shifts for **A-Cl, 1a, 1b** and [**2a**]$^+$ an empirical correction was determined by linear regression to be:

$$\delta_{calc,corr} = (\delta_{calc} - 0.70)/1.0545$$

	[**2a**]$^+$ in CD_2Cl_2				[**3**]$^+$ in CD_2Cl_2				[**4**]$^+$ in CD_2Cl_2			
	σ_{calc}	δ_{calc}	$\delta_{calc,corr}$	δ_{exp}	σ_{calc}	δ_{calc}	$\delta_{calc,corr}$	δ_{exp}	σ_{calc}	δ_{calc}	$\delta_{calc,corr}$	δ_{exp}
Si***C***H_3	186,1	0,8	0,1	0,2	185,9	1,0	0,2	0,7	186,7	0,2	-0,5	0,3
$\boldsymbol{C_\alpha}$	45,1	141,8	133,8	135,5	117,8	69,1	64,9	62,7	22,8	164,1	154,9	150,1
$\boldsymbol{C_\beta}$	-15,2	202,1	190,9	190,3	29,7	157,2	148,4	144,8*	-5,1	192,0	181,4	181,4
Ph*-$\boldsymbol{C_{ipso}}$	42,7	144,2	136,0	137,4	40,9	146,0	137,8	138,3*	41,2	145,7	137,5	138,8
Ph*-$\boldsymbol{C_{ortho}}$					54,5	132,4	124,9	123,8	56,5	130,4	123,0	122,9
Ph*-$\boldsymbol{C_{meta}}$					28,9	158,0	149,2	152,2	29,2	157,7	148,9	150,6
Ph*-$\boldsymbol{C_{para}}$					62,1	124,8	117,7	123,7	55,8	131,1	123,7	123,1
Ph*-***C***Me_3					148,0	38,9	36,3	35,3	148,1	38,8	36,2	35,0
Ph*-C$\boldsymbol{Me}_3$					154,4	32,5	30,2	33,4		186,9	176,5	
B-***C***O									4,3	182,6	172,5	169,5
NHC-B-***C***	28,5	158,4	149,5	150,4	32,6	154,3	145,6	147,0	31,9	155,0	146,3	148,5
N***Me***	150,2	36,7	34,1	34,6	147,7	39,2	36,5	36,9	151,6	35,3	32,8	34,5
N***Me***	150,3	36,6	34,1	34,6	148,8	38,1	35,5	35,9	154,1	32,8	30,4	31,5
NHC-C***Me***					177,6	9,3	8,1	9,2	177,0	9,9	8,7	9,5
NHC-C***Me***					177,3	9,6	8,5	9,2	177,4	9,5	8,3	8,9
NHC-***C***=C	47,0	139,9	132,0	129,7	47,4	139,5	131,6	130,4	49,6	137,3	129,5	128,7
NHC-C=***C***	47,0	139,9	132,0	129,7	49,1	137,8	130,0	129,2	49,3	137,6	129,8	129,2
tolane-***C***					46,8	140,1	132,2	133,9*				
tolane-$\boldsymbol{C_{ipso}}$					45,9	141,0	133,0	n.o.				
tolane-$\boldsymbol{C_{ortho}}$					49,4	137,5	129,7	129,7				
tolane-$\boldsymbol{C_{meta}}$					51,0	135,9	128,1	128,5				
tolane-$\boldsymbol{C_{para}}$					51,8	135,1	127,5	127,8				

Note: * indicates experimental shifts that were not unambiguously assignable by means of experimental 2D-NMR spectra but have been assigned here based on the computational prediction

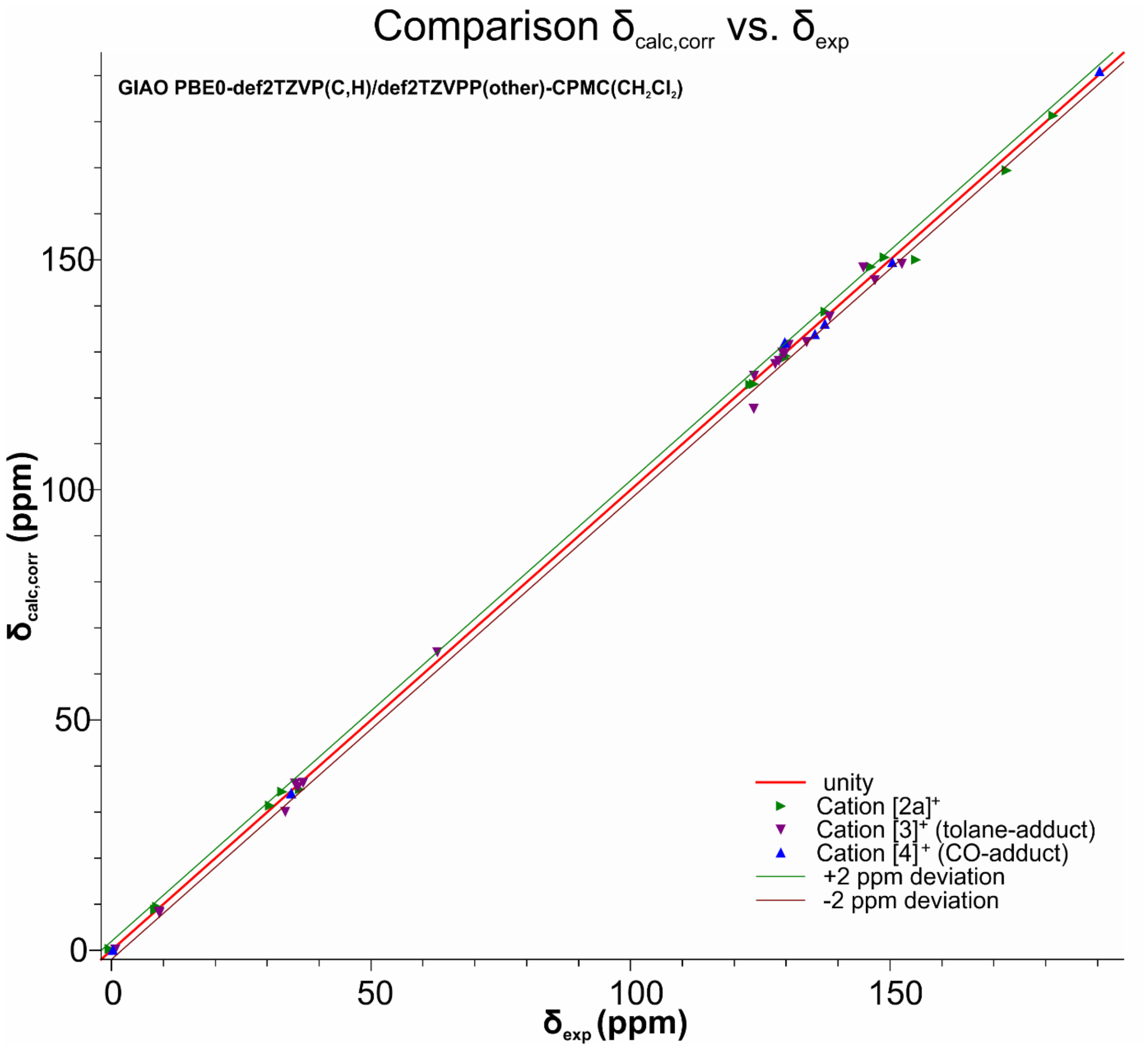

Computational assessment of UVVis spectroscopic features

Regarding UVVis-spectroscopic features, TD-DFT calculations for 10 states using RIJCOSX-approximation with CAM-B3LYP[23] functional and def2-SVP basis sets on all atoms were performed. In case of cationic **[2a]**$^+$ a CPCM model for dichloromethane was applied.

NICS Profiles

$(HC)_4BH$ and $(PhC)_4BPh$ **A-Mes** and **A-C_6F_5** given for comparison and reference.[2b]

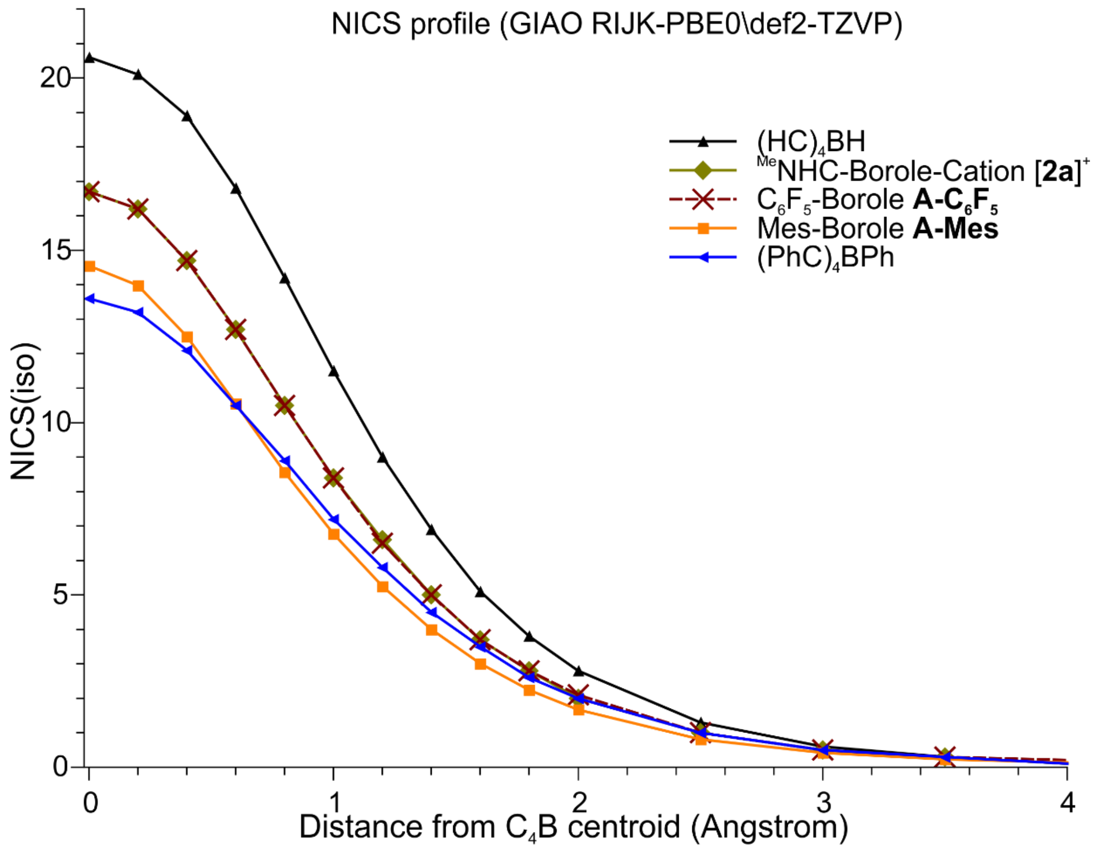
NICS profile (GIAO RIJK-PBE0\def2-TZVP)
(HC)4BH
MeNHC-Borole-Cation [2a]+
C6F5-Borole A-C6F5
Mes-Borole A-Mes
(PhC)4BPh
NICS(iso)
Distance from C4B centroid (Angstrom)
0
5
10
15
20
0
1
2
3
4

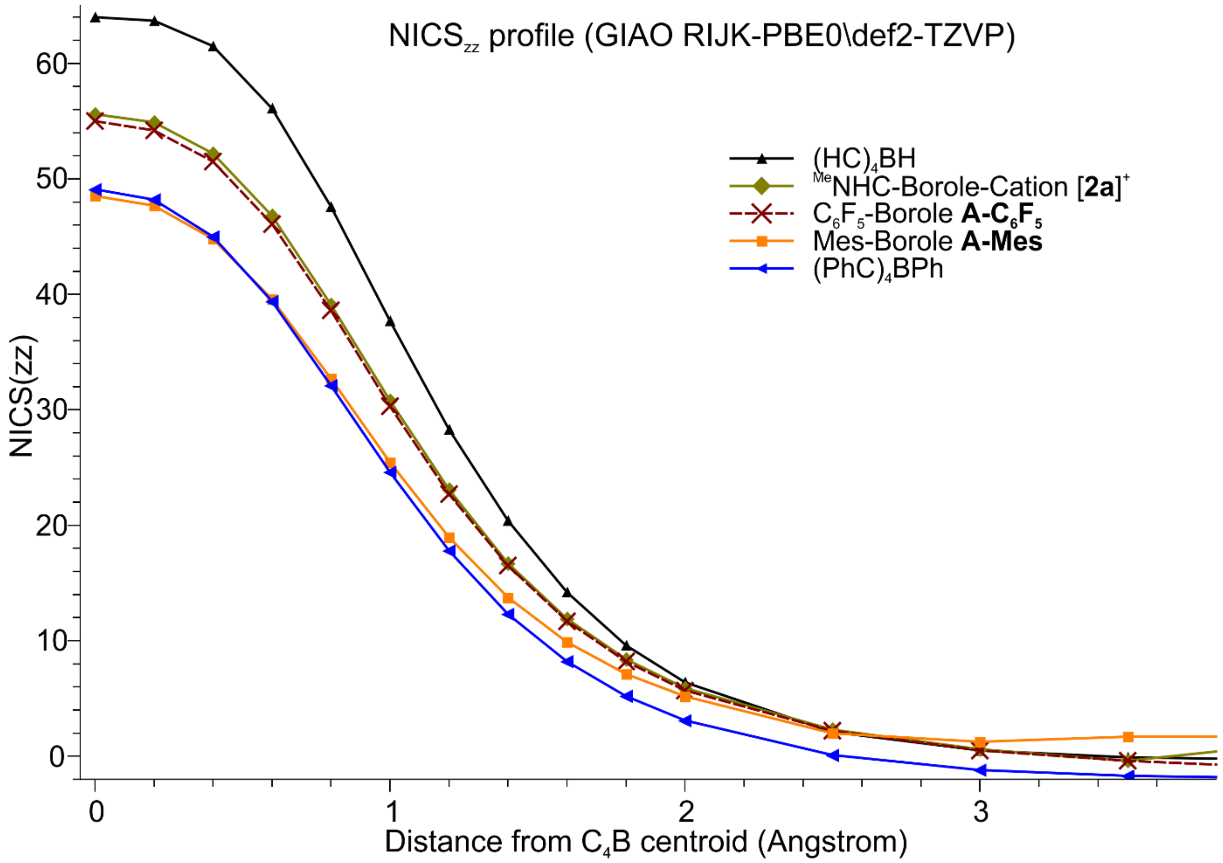
NICSzz profile (GIAO RIJK-PBE0\def2-TZVP)
(HC)4BH
MeNHC-Borole-Cation [2a]+
C6F5-Borole A-C6F5
Mes-Borole A-Mes
(PhC)4BPh
NICS(zz)
Distance from C4B centroid (Angstrom)
0
10
20
30
40
50
60
0
1
2
3

XYZ-coordinates of optimised structures

All structures optimised at RI-BP86-D3BJ\def2TZVP\J level of theory (see above).

Cation [2a]$^{+}$-gasphase (E = -2472.39460142 H)

N	13.185278714	9.382537936	14.092699101
B	12.693088341	11.735270225	13.063062117
Si	15.177887288	12.842630959	14.515691105
C	13.940785726	12.671891066	13.112836099
Si	10.306785515	11.199752013	11.171776580
N	11.460759929	10.380272646	14.928730930
C	13.683643593	13.583390346	12.113531429
C	12.371579402	13.253690994	11.364384393
C	11.717971946	12.182838531	11.926699020
C	12.450176727	10.515757735	14.015944380
C	13.243525445	7.181443469	15.329106884
H	13.247643104	6.546753329	14.430414428
H	12.658080000	6.665806712	16.098338955
H	14.279630766	7.257402368	15.691248678
C	14.320911152	9.090373113	13.225737463
H	14.054441498	8.314848031	12.495891750
H	15.171139666	8.749069978	13.827486641
H	14.596395090	10.010652234	12.697898716
C	11.570494593	9.162442360	15.596361381
C	12.658729540	8.518320278	15.050239719
C	10.636672428	8.756157572	16.677959584
H	9.601031806	8.679284055	16.314651566
H	10.647595962	9.473789073	17.511845738
H	10.923849147	7.777143322	17.077189590
C	10.475206022	11.415414790	15.218034327
H	10.532600677	12.175146212	14.430061127
H	10.683654278	11.879800757	16.190695521
H	9.469510433	10.979688239	15.229207969
C	10.541247223	9.385554137	11.666822476
H	9.795016937	8.763207995	11.150866095
H	10.418737540	9.211285664	12.745219400
H	11.534198631	9.015399019	11.370047970
C	8.628022229	11.764767415	11.824703465
H	8.431063984	12.818272354	11.582083947
H	8.548554029	11.655965456	12.915730783
H	7.823055233	11.164534624	11.374453809
C	10.320044361	11.267082895	9.291317526
H	9.615062239	10.523442948	8.890046146
H	11.319042417	11.032794010	8.896028925
H	10.033340042	12.252009031	8.902404692
C	14.416367776	12.042804301	16.055966451
H	13.434130132	12.480328785	16.290046773
H	15.067569511	12.223828820	16.924067197
H	14.294175111	10.954281489	15.964245264
C	15.522848140	14.641840081	14.946623201
H	16.113766835	15.156858604	14.179306090
H	16.077265560	14.693842141	15.895792643
H	14.584617551	15.201395969	15.072792236
C	16.795727770	11.942849716	14.144537876
H	16.642301655	10.866383520	13.982289242
H	17.493705065	12.052129416	14.988285838
H	17.292194520	12.344089805	13.250200684
C	14.438213021	14.776996664	11.762351268
C	15.838461259	14.734152366	11.626215833
H	16.342074309	13.781819479	11.779727504
C	16.557915412	15.868915696	11.249987271
C	18.689507419	14.483303713	11.363025178
H	18.291109046	13.701359504	10.699923930
H	18.510321528	14.182418035	12.405656936
H	19.777086560	14.521708527	11.212677891
C	18.712480344	16.895093643	12.009988907
H	19.804969315	16.903963500	11.885561757
H	18.488039850	16.653606720	13.058872195
H	18.344677620	17.910757967	11.811296182
C	18.404773263	16.229136517	9.595706811
H	17.967018096	15.499702972	8.899690431
H	19.493387638	16.242443548	9.441271929
H	18.015441646	17.221846998	9.331725196
C	15.840922718	17.058918445	11.035348181
H	16.393081181	17.950598751	10.735095306
C	14.448148899	17.148084659	11.173896171
C	14.291945936	19.560215212	11.845125804
H	15.367073428	19.718324656	11.683636671
H	14.145715818	19.282817977	12.898791655
H	13.782112313	20.518077800	11.667267028
C	13.956133086	18.883288337	9.437319192
H	13.518988615	18.137539082	8.758081732
H	15.024510076	18.978787078	9.199278682
H	13.477791411	19.852460837	9.234506685
C	12.212057333	18.358067850	11.136697025
H	11.734708617	19.325340475	10.927148974
H	11.974392866	18.087739469	12.175983024
H	11.759091465	17.610078556	10.469540243
C	13.758630890	15.988741456	11.523593509
H	12.678395227	16.011172629	11.635827765
C	12.002616819	14.013356510	10.177471265
C	13.714746366	15.234779692	6.964154102
C	13.831935706	13.913413576	6.171895466
H	12.873269279	13.650295245	5.702688168
H	14.126385369	13.083456581	6.830070476
H	14.589441312	14.008228015	5.379952003
C	15.083139625	15.570438434	7.592781251
H	15.823365140	15.746263724	6.799384604
H	15.463454387	14.754164737	8.221128466
H	15.027808404	16.471280596	8.217458321
C	13.335870538	16.365105789	5.994143967
H	14.142322244	16.507309040	5.261697085
H	13.188662976	17.319499706	6.520431702
H	12.420894183	16.136231580	5.429936123
C	12.656510250	15.034061912	8.055070262
C	11.357773159	15.545450324	7.953223676
H	11.101808710	16.159961455	7.091491445
C	8.962807921	15.898713053	8.724694627
C	8.010963762	15.527374547	9.870921794
H	7.024254745	15.975678254	9.691003145
H	8.373044465	15.899888805	10.840388301
H	7.870464465	14.438990485	9.944821489
C	8.372098523	15.355865264	7.405090033
H	8.989954088	15.629596268	6.539214968
H	7.365522185	15.767911087	7.243248002
H	8.294414389	14.259440204	7.432162037
C	9.065770081	17.436830513	8.650542307
H	9.719843807	17.762167819	7.830189017
H	9.467776236	17.848312516	9.587110624
H	8.071641680	17.875490328	8.482143221
C	10.697492628	14.522481351	10.024002173
H	9.973391786	14.329034318	10.812943964
C	18.077958527	15.858339527	11.058019929
C	13.726658990	18.473649876	10.907414894
C	10.361598370	15.304678571	8.921336404
C	12.967165168	14.283006711	9.192764712
H	13.969261237	13.878367930	9.319795196
Si	5.701121000	4.102174000	3.937392000

Cation [2a]$^{+}$-(CPCM = DCM) (E = -2472.45100372 H)

N	13.228082000	9.384741000	14.135677000
B	12.686446000	11.709388000	13.077569000
Si	15.176348000	12.833033000	14.524777000
C	13.937091000	12.654737000	13.124395000
Si	10.298158000	11.207520000	11.165128000
N	11.421548000	10.311245000	14.882498000
C	13.677009000	13.568600000	12.133144000
C	12.367650000	13.241826000	11.382490000

C	11.710699000	12.174506000	11.939068000
C	12.448846000	10.486419000	14.017382000
C	13.316499000	7.194137000	15.378350000
H	13.383063000	6.562263000	14.480092000
H	12.718110000	6.661479000	16.125698000
H	14.333813000	7.317605000	15.778111000
C	14.415457000	9.125398000	13.328105000
H	14.225486000	8.296603000	12.634989000
H	15.258626000	8.871435000	13.980334000
H	14.651121000	10.029130000	12.755964000
C	11.551226000	9.104783000	15.558813000
C	12.693159000	8.504611000	15.067921000
C	10.586749000	8.663781000	16.596839000
H	9.573302000	8.555585000	16.183139000
H	10.535495000	9.385867000	17.425367000
H	10.893584000	7.695377000	17.007060000
C	10.372152000	11.295775000	15.124783000
H	10.476501000	12.102806000	14.391755000
H	10.470076000	11.709318000	16.135960000
H	9.389266000	10.823488000	15.015965000
C	10.471991000	9.396120000	11.680356000
H	9.742603000	8.787957000	11.123886000
H	10.283250000	9.237641000	12.751157000
H	11.475623000	9.008951000	11.449051000
C	8.610847000	11.826436000	11.742236000
H	8.439402000	12.868044000	11.436342000
H	8.505708000	11.775561000	12.835066000
H	7.814628000	11.209397000	11.297978000
C	10.373659000	11.261072000	9.281528000
H	9.645069000	10.547744000	8.866263000
H	11.374181000	10.976375000	8.923867000
H	10.145573000	12.258122000	8.883139000
C	14.447151000	12.008023000	16.062892000
H	13.442089000	12.395880000	16.286668000
H	15.089438000	12.224052000	16.930307000
H	14.378914000	10.915374000	15.968523000
C	15.478425000	14.642987000	14.960801000
H	16.037290000	15.175928000	14.180984000
H	16.053485000	14.700397000	15.897855000
H	14.524585000	15.169889000	15.111726000
C	16.822042000	11.993440000	14.136298000
H	16.698431000	10.915804000	13.959364000
H	17.513329000	12.117926000	14.984081000
H	17.299294000	12.426673000	13.246248000
C	14.426552000	14.770639000	11.782180000
C	15.821192000	14.733426000	11.608564000
H	16.331669000	13.781591000	11.741139000
C	16.526514000	15.876074000	11.224052000
C	18.667169000	14.498315000	11.277571000
H	18.253291000	13.723044000	10.616311000
H	18.511766000	14.184894000	12.320329000
H	19.751228000	14.545801000	11.101802000
C	18.689934000	16.901466000	11.958314000
H	19.780500000	16.916647000	11.813225000
H	18.484775000	16.640976000	13.007088000
H	18.309683000	17.916448000	11.778152000
C	18.345572000	16.268850000	9.542102000
H	17.902970000	15.543892000	8.844344000
H	19.432781000	16.292218000	9.374650000
H	17.942401000	17.261833000	9.300051000
C	15.801533000	17.063768000	11.029173000
H	16.341005000	17.957139000	10.710953000
C	14.412173000	17.143770000	11.206647000
C	14.311003000	19.597275000	11.758007000
H	15.366898000	19.745260000	11.492707000
H	14.256260000	19.381697000	12.835225000
H	13.780599000	20.542063000	11.567561000
C	13.770949000	18.780502000	9.432803000
H	13.287409000	17.990216000	8.840274000
H	14.817393000	18.864049000	9.106957000
H	13.266317000	19.733471000	9.213863000
C	12.181905000	18.363352000	11.309886000
H	11.694006000	19.328432000	11.112479000
H	12.041612000	18.122889000	12.374031000
H	11.665209000	17.599205000	10.711273000
C	13.737720000	15.980340000	11.573623000
H	12.660239000	15.995561000	11.713120000
C	12.008029000	14.007574000	10.190990000
C	13.745086000	15.169532000	6.973799000
C	13.897913000	13.821485000	6.234642000
H	12.949861000	13.525614000	5.761940000
H	14.197259000	13.023616000	6.929331000
H	14.666199000	13.902245000	5.450720000
C	15.097225000	15.552512000	7.610801000
H	15.853566000	15.689970000	6.824152000
H	15.465165000	14.777697000	8.296045000
H	15.017638000	16.486454000	8.182276000
C	13.363333000	16.254537000	5.955065000
H	14.172853000	16.366503000	5.219797000
H	13.211097000	17.229466000	6.441308000
H	12.447530000	15.997263000	5.403867000
C	12.678322000	14.996755000	8.061087000
C	11.398523000	15.554453000	7.964681000
H	11.156702000	16.172961000	7.101546000
C	9.026551000	15.996799000	8.756551000
C	8.087499000	15.708314000	9.936760000
H	7.119167000	16.198680000	9.763058000
H	8.494399000	16.090766000	10.884267000
H	7.898873000	14.630721000	10.050032000
C	8.387101000	15.426796000	7.471313000
H	9.002233000	15.641121000	6.586370000
H	7.394368000	15.874045000	7.312658000
H	8.266754000	14.336160000	7.547904000
C	9.182115000	17.526110000	8.617523000
H	9.825311000	17.793319000	7.767975000
H	9.624193000	17.957371000	9.526935000
H	8.198132000	17.991694000	8.458321000
C	10.723816000	14.568146000	10.051024000
H	10.002826000	14.409343000	10.850568000
C	18.043347000	15.874453000	11.003536000
C	13.670056000	18.458186000	10.939337000
C	10.406136000	15.355719000	8.945091000
C	12.970762000	14.234806000	9.197114000
H	13.959648000	13.795576000	9.318374000

Cation $[3]^{+}$-gasphase (E = -3012.16901537 H)

Si	14.079602000	2.686922000	5.456212000
Si	9.805184000	2.572110000	9.699679000
C	14.759933000	-2.914003000	5.195516000
C	14.894141000	-1.962971000	6.393930000
C	11.879288000	-1.223649000	9.526787000
H	11.722605000	-1.496240000	8.482240000
C	12.619245000	-0.018316000	13.630147000
C	11.840655000	-2.200691000	10.526166000
C	8.213851000	1.151065000	5.733862000
H	8.573071000	1.780083000	4.921155000
C	12.075724000	-1.798563000	11.849054000
H	12.044320000	-2.545406000	12.637837000
C	12.168358000	0.111828000	9.827132000
C	9.025824000	0.900475000	6.853200000
C	14.130408000	0.031476000	7.578300000
C	14.994471000	-0.270239000	8.636184000
H	14.996666000	0.385327000	9.506719000
C	15.737902000	-2.240255000	7.479914000
H	16.352188000	-3.135978000	7.452346000
C	11.111958000	1.233490000	4.575953000
C	14.104186000	-0.808188000	6.454334000
H	13.409092000	-0.581918000	5.644710000
C	13.107814000	1.083921000	7.713136000
C	8.565927000	0.008821000	7.837790000
H	9.217530000	-0.236080000	8.675972000
C	12.418925000	0.475396000	11.152641000

H	12.665189000	1.513483000	11.376080000
C	10.968314000	1.891760000	8.349223000
C	12.200690000	1.090449000	8.717687000
C	11.360585000	1.523291000	6.000425000
C	11.583690000	-3.658426000	10.130394000
C	12.694109000	1.977424000	6.562452000
C	6.492355000	-0.295407000	6.633587000
H	5.505797000	-0.751939000	6.549774000
C	15.803657000	-1.409939000	8.608288000
C	12.368192000	-0.469234000	12.186076000
C	7.311182000	-0.587710000	7.726681000
H	6.975287000	-1.285080000	8.494534000
C	10.398378000	1.405802000	7.028132000
C	16.686092000	-1.727877000	9.820664000
C	6.950724000	0.570174000	5.635657000
H	6.325443000	0.783240000	4.768137000
C	13.645004000	4.427380000	4.843201000
H	14.411733000	4.733836000	4.115537000
H	13.647000000	5.176853000	5.645609000
H	12.676665000	4.487424000	4.329523000
C	15.780973000	-4.060660000	5.244599000
H	15.657749000	-4.701510000	4.360294000
H	15.643495000	-4.693800000	6.132558000
H	16.814012000	-3.684293000	5.244626000
C	13.340732000	-3.523822000	5.206952000
H	12.571616000	-2.739936000	5.157690000
H	13.171033000	-4.107940000	6.121934000
H	13.203073000	-4.190867000	4.342871000
C	11.020289000	2.226970000	3.594043000
H	11.093307000	3.275721000	3.878501000
C	11.535724000	-4.596303000	11.344878000
H	11.347317000	-5.625715000	11.009294000
H	10.731552000	-4.320157000	12.042282000
H	12.486681000	-4.596858000	11.896583000
C	14.496738000	1.632235000	3.960963000
H	13.630849000	1.419017000	3.321541000
H	14.937612000	0.678504000	4.275325000
H	15.244935000	2.159235000	3.349157000
C	15.779570000	-1.887551000	11.060704000
H	15.183488000	-0.984846000	11.247579000
H	15.075006000	-2.720199000	10.927143000
C	14.007326000	0.649226000	13.729108000
H	14.802538000	-0.059920000	13.460191000
H	14.189580000	0.995423000	14.756983000
H	14.090047000	1.519102000	13.062769000
C	11.022327000	-0.114968000	4.197328000
H	11.077676000	-0.889439000	4.963184000
C	15.613977000	2.819476000	6.537566000
H	16.383950000	3.414067000	6.023471000
H	16.033332000	1.825288000	6.741444000
H	15.413842000	3.292385000	7.508666000
C	12.724331000	-4.117357000	9.195469000
H	12.807201000	-3.471402000	8.311374000
H	12.549826000	-5.149419000	8.856690000
H	13.692482000	-4.084975000	9.714705000
C	12.574150000	-1.188510000	14.623595000
H	13.339288000	-1.944389000	14.395353000
H	11.591379000	-1.681285000	14.630785000
H	12.765770000	-0.816647000	15.639803000
C	17.492931000	-3.021026000	9.635833000
H	18.173986000	-2.958669000	8.774802000
H	16.837851000	-3.893180000	9.496303000
H	18.103939000	-3.205140000	10.530601000
C	10.781323000	0.536096000	1.879082000
H	10.660357000	0.265524000	0.830121000
C	10.236537000	-3.753077000	9.382277000
H	10.232794000	-3.136554000	8.472672000
H	9.410138000	-3.416464000	10.025143000
H	10.037906000	-4.792925000	9.084134000
C	17.670524000	-0.562042000	10.052651000
H	18.322337000	-0.420659000	9.178616000
H	18.306342000	-0.768177000	10.926256000
H	17.140524000	0.382711000	10.236470000
C	8.402425000	3.557717000	8.907256000
H	8.768491000	4.472527000	8.420571000
H	7.701451000	3.870761000	9.695813000
H	7.832496000	2.970605000	8.174821000
C	9.054539000	1.282317000	10.848523000
H	8.126108000	0.869510000	10.432782000
H	8.802935000	1.764835000	11.804966000
H	9.741496000	0.454845000	11.065795000
C	10.854209000	1.880349000	2.252121000
H	10.789653000	2.662302000	1.494781000
C	10.819384000	3.796396000	10.721450000
H	10.937798000	4.748799000	10.185475000
H	11.818018000	3.416883000	10.973782000
H	10.300252000	4.017144000	11.665272000
C	14.959031000	-2.139283000	3.877637000
H	15.944682000	-1.653042000	3.850191000
H	14.191312000	-1.365991000	3.739192000
H	14.894140000	-2.826773000	3.021515000
C	11.530176000	1.003480000	14.024263000
H	11.544984000	1.881846000	13.363823000
H	11.687420000	1.351583000	15.055792000
H	10.530184000	0.550912000	13.962284000
C	10.863286000	-0.460274000	2.856109000
H	10.803918000	-1.511542000	2.573381000
B	11.434415000	2.856457000	7.114929000
C	11.192839000	4.391937000	6.982458000
C	11.577646000	6.583667000	7.403969000
C	10.486035000	6.502485000	6.571526000
C	9.168756000	4.621028000	5.542499000
H	9.252170000	4.950974000	4.499514000
H	9.199397000	3.531612000	5.586111000
H	8.214245000	4.963559000	5.960274000
C	13.140481000	4.904317000	8.455252000
H	14.065508000	5.204860000	7.946936000
H	13.081508000	5.398025000	9.431371000
H	13.141387000	3.818502000	8.598566000
C	9.621501000	7.560441000	5.987752000
H	9.975748000	8.549836000	6.297469000
H	9.628840000	7.531371000	4.887991000
H	8.576678000	7.461129000	6.318355000
C	12.272671000	7.757559000	7.992389000
H	13.331345000	7.791931000	7.695500000
H	11.799717000	8.686119000	7.654197000
H	12.234136000	7.742767000	9.091894000
N	10.273565000	5.144979000	6.331697000
N	11.984512000	5.275029000	7.645240000
H	16.388183000	-2.090353000	11.954552000

Cation $[4]^+$-gasphase (E = -2585.80115849 H)

N	13.655516000	9.628304000	12.725662000
B	12.406795000	11.787577000	13.360425000
Si	14.847151000	13.019577000	14.839874000
C	13.589732000	12.916055000	13.451659000
Si	10.189395000	11.261649000	11.173265000
N	12.407997000	9.349832000	14.469962000
C	13.405887000	13.728010000	12.358027000
C	12.248719000	13.315717000	11.517727000
C	11.604050000	12.197337000	11.984590000
C	12.804545000	10.272437000	13.558779000
C	14.654374000	7.337676000	12.348744000
H	14.375683000	7.275999000	11.286614000
H	14.564141000	6.333122000	12.776592000
H	15.714693000	7.627530000	12.402348000
C	14.287959000	10.249643000	11.566968000
H	13.676253000	10.093406000	10.668908000
H	15.280688000	9.813006000	11.417930000
H	14.387726000	11.323429000	11.758788000
C	12.992360000	8.108760000	14.197259000
C	13.785137000	8.288507000	13.089749000

C	12.739166000	6.895863000	15.018620000
H	11.671032000	6.635023000	15.044767000
H	13.084648000	7.021378000	16.055989000
H	13.278398000	6.041203000	14.595058000
C	11.445579000	9.590865000	15.538592000
H	11.633565000	10.568227000	15.994471000
H	11.567209000	8.825488000	16.310470000
H	10.418038000	9.548895000	15.152351000
C	10.207959000	9.487309000	11.827076000
H	9.411978000	8.906790000	11.337641000
H	10.013325000	9.446756000	12.908780000
H	11.157859000	8.969440000	11.630265000
C	8.531655000	12.023461000	11.636558000
H	8.425861000	13.032726000	11.216696000
H	8.407121000	12.099050000	12.726695000
H	7.704790000	11.410388000	11.247810000
C	10.421167000	11.221667000	9.308256000
H	9.673081000	10.555276000	8.853123000
H	11.417570000	10.846295000	9.033616000
H	10.309611000	12.218851000	8.863726000
C	14.041747000	12.279174000	16.380729000
H	13.125353000	12.812845000	16.672619000
H	14.734743000	12.339007000	17.232885000
H	13.802336000	11.214997000	16.238028000
C	15.388166000	14.777375000	15.208338000
H	15.945898000	15.222455000	14.374924000
H	16.030198000	14.793451000	16.101742000
H	14.517615000	15.419291000	15.405825000
C	16.312571000	11.915339000	14.392945000
H	15.987162000	10.876072000	14.233743000
H	17.052496000	11.908422000	15.207269000
H	16.825434000	12.254261000	13.482292000
C	14.236192000	14.875803000	11.955793000
C	15.621617000	14.742444000	11.796125000
H	16.080633000	13.777549000	12.004926000
C	16.399211000	15.815527000	11.347294000
C	18.450540000	14.318835000	11.535048000
H	18.015057000	13.539174000	10.892272000
H	18.244198000	14.062486000	12.584768000
H	19.540170000	14.291416000	11.396903000
C	18.593462000	16.745454000	12.108268000
H	19.687161000	16.694960000	12.004248000
H	18.338297000	16.549206000	13.159665000
H	18.278918000	17.770740000	11.871566000
C	18.296366000	16.019876000	9.711899000
H	17.838450000	15.290700000	9.029006000
H	19.387571000	15.974737000	9.583176000
H	17.963484000	17.020940000	9.406746000
C	15.743553000	17.020034000	11.053034000
H	16.335046000	17.857599000	10.681126000
C	14.359439000	17.189633000	11.205066000
C	14.410075000	19.693342000	11.493170000
H	15.463894000	19.761078000	11.191291000
H	14.376461000	19.596652000	12.587723000
H	13.924911000	20.640566000	11.216953000
C	13.774448000	18.659991000	9.279090000
H	13.246424000	17.831862000	8.784239000
H	14.817124000	18.660081000	8.931395000
H	13.310386000	19.604989000	8.960452000
C	12.207358000	18.545007000	11.217956000
H	11.771405000	19.513338000	10.935438000
H	12.077935000	18.422715000	12.303252000
H	11.631386000	17.759500000	10.707568000
C	13.618497000	16.099144000	11.662348000
H	12.541102000	16.181813000	11.782210000
C	11.944865000	14.030942000	10.263477000
C	13.801942000	14.871298000	7.022441000
C	13.954037000	13.457466000	6.420137000
H	13.016281000	13.128275000	5.950237000
H	14.221619000	12.723028000	7.193331000
H	14.744474000	13.450379000	5.655240000
C	15.136663000	15.296629000	7.670290000
H	15.928476000	15.337444000	6.908348000
H	15.458252000	14.594130000	8.450755000
H	15.053312000	16.287207000	8.136040000
C	13.474515000	15.858746000	5.892649000
H	14.302075000	15.879156000	5.170104000
H	13.339308000	16.881006000	6.274687000
H	12.566541000	15.570124000	5.344169000
C	12.703605000	14.826504000	8.089994000
C	11.471167000	15.471846000	7.943139000
H	11.283104000	16.044178000	7.036372000
C	9.133225000	16.141636000	8.678077000
C	8.204139000	16.075228000	9.899095000
H	7.275476000	16.622343000	9.686323000
H	8.664670000	16.531947000	10.787370000
H	7.924702000	15.040292000	10.145131000
C	8.425183000	15.467137000	7.482538000
H	9.036736000	15.519580000	6.571389000
H	7.466177000	15.964700000	7.276807000
H	8.224301000	14.406968000	7.694553000
C	9.395008000	17.626446000	8.348783000
H	10.023414000	17.744739000	7.456115000
H	9.897134000	18.132356000	9.185084000
H	8.443433000	18.143152000	8.157839000
C	10.716655000	14.686353000	10.085241000
H	9.982068000	14.642382000	10.887196000
C	17.919415000	15.714876000	11.176705000
C	13.689548000	18.511680000	10.814019000
C	10.463276000	15.420026000	8.923431000
C	12.923296000	14.107179000	9.270522000
H	13.878357000	13.608812000	9.436309000
C	11.410395000	12.466643000	14.298227000
O	10.679786000	13.146619000	14.868473000

Model complex $(HC)_4B(^{Mr}NHC)$

N	4.069110970	4.196663080	15.986517720
B	4.363106102	6.259645539	14.408023273
C	3.104864777	6.666158267	13.558977898
N	4.699773745	6.011291999	16.986960317
C	3.603120239	7.343341586	12.501731565
C	5.622873942	6.789605467	13.630586552
C	5.124632015	7.381174017	12.523366445
C	4.365991812	5.500356401	15.779246607
C	3.678200571	3.265761448	14.928858648
H	3.457524175	3.838368081	14.020091718
H	2.780183593	2.715969878	15.232721229
H	4.490538973	2.557366603	14.723004844
C	5.074491776	7.406482569	17.212418075
H	5.268454701	7.879597977	16.242281685
H	5.984709365	7.451017150	17.821024613
H	4.264688046	7.943175846	17.722639801
C	4.217251217	3.872331619	17.333067283
C	4.602216498	5.031992183	17.972788121
C	3.983727885	2.501441217	17.855884021
H	2.941408811	2.181375797	17.709220832
H	4.195848322	2.466155602	18.930046184
H	4.634559082	1.764364014	17.362490628
C	4.876933627	5.309688631	19.406177431
H	5.919943785	5.619718832	19.568682521
H	4.698805979	4.410219425	20.005590266
H	4.224843289	6.105463792	19.795801596
H	2.041830452	6.534942300	13.751475304
H	3.025505209	7.832130650	11.714698032
H	5.701685222	7.832561446	11.713924185
H	6.685765478	6.687930706	13.841016585

Literature

[1] R. K. Harris, E. D. Becker, S. M. Cabral de Menezes, R. Goodfellow, P. Granger, *Pure Appl. Chem.* **2001**, *73*, 1795-1818.

[2] a) S. Yruegas, J. J. Martinez, C. D. Martin, *Chem. Commun.* **2018**, *54*, 6808-6811; b) T. Heitkemper, L. Naß, C. P. Sindlinger, *Dalton Trans.* **2020**, *49*, 2706 - 2714.

[3] a) U. Mayer, V. Gutmann, W. Gerger, *Montash. Chem.* **1975**, *106*, 1235-1257; b) M. A. Beckett, G. C. Strickland, J. R. Holland, K. Sukumar Varma, *Polymer* **1996**, *37*, 4629-4631; c) I. B. Sivaev, V. I. Bregadze, *Coord. Chem. Rev.* **2014**, *270-271*, 75-88.

[4] I. Krossing, *Chem. Eur. J.* **2001**, *7*, 490-502.

[5] a) I. Raabe, K. Wagner, K. Guttsche, M. Wang, M. Grätzel, G. Santiso-Quiñones, I. Krossing, *Chem. Eur. J.* **2009**, *15*, 1966-1976; b) M. P. Stewart, L. M. Paradee, I. Raabe, N. Trapp, J. S. Slattery, I. Krossing, W. E. Geiger, *J. Fluor. Chem.* **2010**, *131*, 1091-1095.

[6] N. Kuhn, T. Kratz, *Synthesis* **1993**, 561-562.

[7] SAINTv8.30C, Bruker AXS, WI, USA, Madison, **2013**.

[8] G. M. Sheldrick, SADABS, **2008** University of Göttingen, Göttingen, Germany.

[9] G. M. Sheldrick, *Acta Crystallogr.* **2015**, *A71*, 3.

[10] G. M. Sheldrick, *Acta Crystallogr.* **2015**, *C71*, 3.

[11] C. B. Hübschle, G. M. Sheldrick, B. Dittrich, *J. Appl. Crystallogr.* **2011**, *44*, 1281-1284.

[12] D. Kratzert, I. Krossing, *J. Appl. Crystallogr.* **2018**, *51*, 928-934.

[13] a) F. Neese, *Wiley Interdiscip. Rev. Comput. Mol. Sci.* **2012**, *2*, 73-78; b) F. Neese, *Wiley Interdiscip. Rev. Comput. Mol. Sci.* **2018**, *8*, e1327.

[14] a) E. D. Glendening, C. R. Landis, F. Weinhold, *Wiley Interdiscip. Rev. Comput. Mol. Sci.* **2012**, *2*, 1-42; b) E. D. Glendening, J. K. Badenhoop, A. E. Reed, J. E. Carpenter, J. A. Bohmann, C. M. Morales, P. Karafiloglou, C. R. Landis, F. Weinhold, Theoretical Chemical Institute, University of Wisconsin Madison, **2018**.

[15] S. Grimme, S. Ehrlich, L. Goerigk, *J. Comput. Chem.* **2011**, *32*, 1456-1465.

[16] a) A. D. Becke, *Phys. Rev. A* **1988**, *38*, 3098-3100; b) J. P. Perdew, W. Yue, *Phys. Rev. B* **1986**, *33*, 8800-8802; c) A. Schäfer, C. Huber, R. Ahlrichs, *J. Chem. Phys.* **1994**, *100*, 5829-5835; d) F. Weigend, R. Ahlrichs, *Phys. Chem. Chem. Phys.* **2005**, *7*, 3297-3305; e) K. Eichkorn, F. Weigend, O. Treutler, R. Ahlrichs, *Theor. Chem. Acc.* **1997**, *97*, 119-124.

[17] G. A. Zhurko, ChemCraft, **2014,** https://www.chemcraftprog.com/.

[18] M. P. Mitoraj, A. Michalak, T. Ziegler, *J. Chem. Theory Comput.* **2009**, *5*, 962-975.

[19] E. Van Lenthe, E. J. Baerends, *J. Comput. Chem.* **2003**, *24*, 1142-1156.

[20] a) G. te Velde, F. M. Bickelhaupt, E. J. Baerends, C. Fonseca Guerra, S. J. A. van Gisbergen, J. G. Snijders, T. Ziegler, *J. Comput. Chem.* **2001**, *22*, 931-967; b) SCM, SCM, Theoretical Chemistry, Vrije Universiteit, Amsterdam, The Netherlands, **2019**.

[21] a) P. v. R. Schleyer, C. Maerker, A. Dransfeld, H. Jiao, N. J. R. van Eikema Hommes, *J. Am. Chem. Soc.* **1996**, *118*, 6317-6318; b) Z. Chen, C. S. Wannere, C. Corminboeuf, R. Puchta, P. v. R. Schleyer, *Chem. Rev.* **2005**, *105*, 3842-3888.

[22] J. P. Perdew, K. Burke, M. Ernzerhof, *Phys. Rev. Lett.* **1996**, *77*, 3865-3868.

[23] T. Yanai, D. P. Tew, N. C. Handy, *Chem. Phys. Lett.* **2004**, *393*, 51-57.
